# High-Resolution Relaxometry for Fragment Screening

**DOI:** 10.1021/acs.jmedchem.6c01773

**Published:** 2026-08-12

**Authors:** Giulia Licciardi, Linda Cerofolini, Ulric Le Paige, Adam Kubrak, Letizia Fiorucci, Luis Padilla-Cortés, Enrico Ravera, Marco Fragai, Claudio Luchinat, Fabien Ferrage, Giacomo Parigi

**Affiliations:** † Department of Chemistry “Ugo Schiff” And Magnetic Resonance Center (CERM), 9300 University of Florence, Sesto Fiorentino 50019, Italy; ‡ Consorzio Interuniversitario Risonanze Magnetiche MetalloProteine (CIRMMP), Via Sacconi 6, Sesto Fiorentino 50019, Italy; § Chimie Physique et Chimie du Vivant, CPCV, Département de Chimie, École Normale supérieure, PSL University, Sorbonne Université, CNRS, Paris 75005, France

## Abstract

Characterizing weak molecular interactions remains a major challenge in drug discovery. Here, we demonstrate the power of high-resolution relaxometry (HRR), using a fast sample shuttle system, as a highly sensitive ligand-based screening method. By measuring longitudinal relaxation rates over a wide magnetic field range (2–600 MHz), we obtained site-specific Nuclear Magnetic Resonance Dispersion (NMRD) profiles for three ligands interacting with the catalytic domain of Human Matrix Metalloproteinase-12 (MMP-12). HRR unambiguously detects weak binding (*K*_*D*_ ≈ 10^–6^–10^–4^) at protein concentrations as low as 2 μM. The analysis of the NMRD profiles provides direct, quantitative assessment of the complex dynamics, yielding a rotational correlation time in excellent agreement with the protein hydrodynamic properties. This enables discrimination between genuine protein–ligand binding and alternative processes like ligand aggregation, often observed in pan-assay interference compounds (PAINS). HRR therefore provides a robust, low-sample-consumption, physicochemical approach for fragment screening and characterization of protein–ligand complex dynamics.

## Graphical abstract

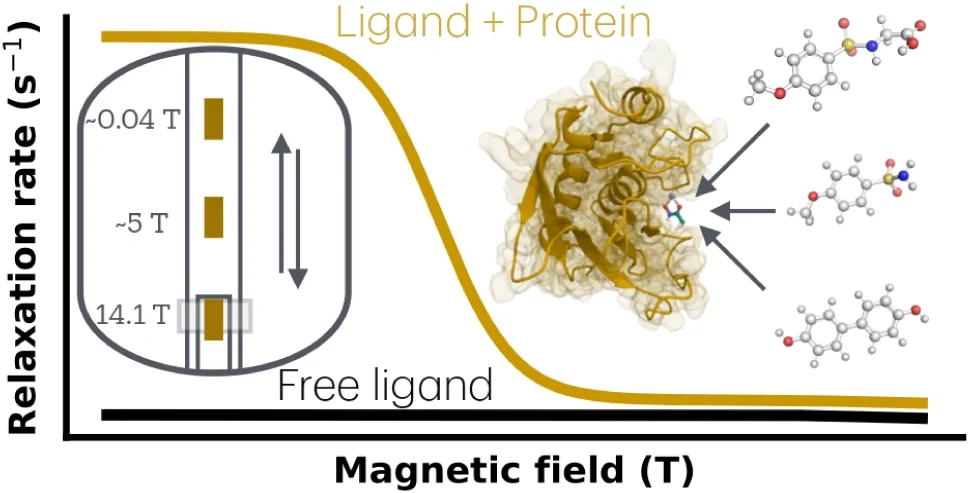

## Introduction

Fragment screening is of paramount importance in drug design and pharmaceutical research.^1^ Several fragment- or ligand-based NMR experiments have been developed to monitor binding to proteins.^2–4^ These experiments, based on the detection of the small molecule, monitor changes in the NMR signals upon binding to a protein, with the advantage of requiring a much smaller amount of protein than protein-based experiments, where protein signals are detected. Their sensitivity is determined by the attainable concentration of the small molecule, which is usually in the millimolar/high micromolar range.

False positives in NMR fragment screening experiments, such as STD, WaterLOGSY, and T2-Edited experiments, present significant challenges in the early stages of the drug discovery pipeline.^5^ A major source of these false positives is fragment aggregation,^6^ which commonly occurs during fragment-based drug discovery (FBDD) due to the high concentrations required to detect binding.^7,8^ In fact, the aggregation of small molecules can lead to false positive responses in these experiments because the aggregates behave similarly to large macromolecules, often simulating protein–ligand interactions.^5^ Although NMR strategies for the characterization of the aggregation state have been developed,^9^ an all-in-one experiment represents a significant advancement in the field of FBDD. Furthermore, the requirement for relatively high concentrations can severely restrict the detection of interactions involving poorly soluble compounds.

NMR relaxation is a very sensitive property that depends on molecular dynamics and, as a consequence, on its size and rigidity. The measurement of nuclear magnetic relaxation dispersion (NMRD) profiles, i.e., of the magnetic-field dependence of the relaxation rates over several orders of magnitude, can be used to determine the reorientation time of the molecule and therefore infer its size. Fast field-cycling (FFC) relaxometers can measure the longitudinal relaxation rates from few hundreds μT to few T, thus providing an excellent sensitivity to dynamics occurring with correlation times ranging from nanoseconds to microseconds.^10–12^ FFC relaxometry, however, does not provide any spectral resolution, so that only the collective relaxation of all nuclei present in the sample can be probed, thus preventing the observation of specific ligands. This drawback can be solved through high-resolution relaxometry (HRR). This technique combines the high-resolution of high-field spectrometers together with the possibility to measure relaxation rates over a wide range of magnetic fields by exploiting the stray field of an NMR magnet.^13–20^

A very recent breakthrough has been provided by the implementation of a fast sample shuttle system (FSS), able to mechanically shuttling the sample within the stray field of a 600 MHz spectrometer in 61 ms, to measure relaxation rates down to fields as low as 36 mT (proton resonance frequency 1.5 MHz), ensuring coverage of more than 2 orders of magnitude field strength as well as high resolution through high field detection.^16^ Fast and reproducible transfer between high and low fields is required to minimize polarization losses due to longitudinal relaxation. The FSS is a hybrid pneumatic–mechanical sample shuttle system allowing the sample to move very fast (up to 100 km/h of maximum speed) forth and back from the high magnetic field center of the magnet and any selected low field position. This hybrid approach combines the mechanical upward force applied to a 6 mm diameter sample container (that includes a 5 mm glass tube) by a cord, pulled by a motor and winch wheel, with the downward force applied by continuous gas flow high pressure (4.5 bar) that pushes the container to the direction of high field detection position. The magnetic field varies by ±10% over the active sample volume across the entire field range.

Human matrix metalloproteinases (MMPs) are a well-known class of proteolytic enzymes that drive the metabolic turnover of extracellular matrix proteins.^21–27^ These multidomain enzymes are crucial in a number of physiological processes,^28–31^ which makes their overexpression implicated in a wide range of pathologies. The development of MMP inhibitors thus represents an important therapeutic research target.^32–47^ The three small molecule fragments shown in Figure 1, 4,4-biphenol (BPN), 4-methoxybenzenesulfonamide glycine (MLC) and 4-methoxybenzenesulfonamide (MBS) are known to bind MMP-12 with large dissociation constants.^48–50^ The protein also binds acetohydroxamic acid (AHA), which is a common additive in protein solutions to protect the enzyme against self-proteolysis. AHA binds to the catalytic metal and is not a competitor of the BPN, MLC and MBS fragments. These fragments bind in the S_1_′ hydrophobic pocket of the protein, and not at the metal binding site. MLC, possessing a carboxylate group, potentially competes with AHA, but in its presence, it can still bind in the S_1_′ pocket.^51^

**Figure 1:**
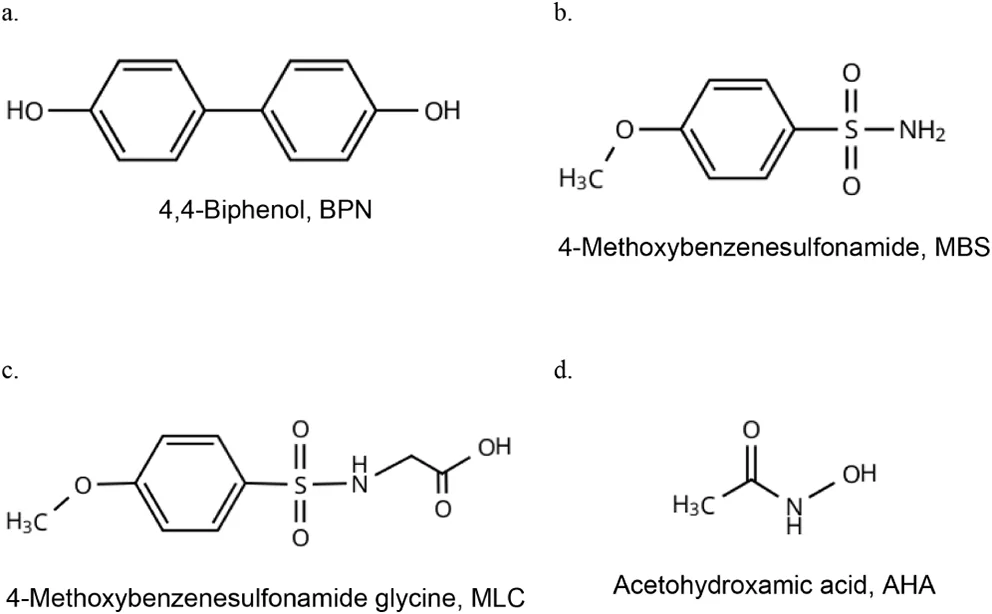
Chemical structures of the considered fragments: a. 4,4-biphenol (BPN); b. 4-methoxybenzenesulfonamide (MBS); c. 4-methoxybenzenesulfonamide glycine (MLC); d. hydroxamic acid (AHA).

High-resolution relaxometry is here applied to monitor the occurrence of interactions between the catalytic domain of MMP-12 (17 kDa molecular weight) and the BPN, MLC and MBS fragments, in the presence of AHA (Figure 1), through the measurement of the field dependence of the relaxation rates of fragment nuclei. We demonstrate that HRR can readily reveal these weak interactions and, more importantly, determine the reorientation time of the resulting complexes, thereby providing independent evidence that the observed binding occurs with the intended target.

## Theoretical Background

Proton relaxation rates due to proton–proton dipole–dipole interactions modulated by stochastic fluctuations occurring with a correlation time *τ_c_* are described by the Solomon equation^52^

1 $$R_{1}=\frac{3}{10}{\left(\frac{\mu_{0}}{4\pi }\frac{\hbar \gamma_{I}^{2}}{r^{3}}\right)}^{2}\left[\frac{\tau_{c}}{1+\omega_{I}^{2}\tau_{c}^{2}}+\frac{4\tau_{c}}{1+4\omega_{I}^{2}\tau_{c}^{2}}\right]$$

where ω_*I*_ is 2π times the proton Larmor frequency, equal to the product between the applied magnetic field and the nuclear magnetogyric ratio, *r* is the internuclear distance, *τ_c_* is the correlation time for rotational diffusion of the molecule and the other symbols have the usual meaning. This model provides relaxation rate values which are constant at low fields, until a dispersion appears with increasing field. The frequency at which the dispersion occurs depends on the value of the correlation time: in case of *τ_c_* of nanoseconds or longer the dispersion is positioned at proton Larmor frequencies smaller than 100 MHz, and thus it can be observed only through low-field relaxation measurements.

The correlation time of nonexchangeable ligand nuclei is given by the molecular reorientation time: for unbound ligands, it can thus range from few tens of picoseconds to hundred picoseconds,^14,53^ whereas if the ligand is bound to macromolecules, like proteins, it can increase to nanoseconds or longer.^54^ The relaxation rates of the observed ligand nuclei thus depend on the reorientation times of the unbound and bound ligand and on their respective populations, which is determined by the ligand (*c*_ligand_) and protein (*c*_protein_) concentrations and by their dissociation constant *K*_*D*_:

2 $$R_{1}^{\mathrm{ligand}}=\frac{c_{\mathrm{free}}}{c_{\mathrm{ligand}}}R_{1}^{\mathrm{free}}+\frac{c_{\mathrm{bound}}}{c_{\mathrm{ligand}}}{\left(1/R_{1}^{\mathrm{bound}}+\tau_{M}\right)}^{-1}$$

with

3 $$c_{\mathrm{bound}}=\left[\left(c_{\mathrm{ligand}}+c_{\mathrm{protein}}+K_{D}\right)-\sqrt{{\left(c_{\mathrm{ligand}}+c_{\mathrm{protein}}+K_{D}\right)}^{2}-4c_{\mathrm{ligand}}c_{\mathrm{protein}}}\right]/2$$

when the ligand can exchange between the bound and unbound state, with lifetime *τ_M_* in the bound form. In eq 2, *c*_free_ and *c*_bound_ are the concentrations of the unbound and bound forms of the ligand, respectively, and *c*_ligand_ = *c*_free_ + *c*_bound_. $R_{1}^{\mathrm{free}}$ and $R_{1}^{\mathrm{bound}}$ can be calculated from eq 1, using the appropriate values for the correlation times *τ_c_* for the unbound and bound ligand. The very small correlation time of the free ligand provides $R_{1}^{\mathrm{free}}$ values much smaller than $R_{1}^{\mathrm{bound}}$ at low fields and basically constant in the whole range of investigated frequencies.

Figure 2 shows the simulated field dependences of $R_{1}^{\mathrm{ligand}}$ for protons in “isolated” methylene groups (proton–proton distance of 1.75 Å), when the correlation time *τ_c_* for the ligand in the bound form is 10 ns and $R_{1}^{\mathrm{free}}$ is 0.5 s^–1^. We have here assumed a protein concentration as low as 2 μM, a ligand concentration of 200 μM, and different values for the dissociation constant. The ligand is assumed in fast exchange $\left(\tau_{M}\ll 1/R_{1}^{\mathrm{bound}}\right)$ . In these conditions, with a protein concentration 100 times smaller than the ligand concentration, the dispersion is clearly visible up to dissociation constants as large as 10^–3^. The dotted line in the figure shows the expected field dependence of $R_{1}^{\mathrm{bound}}$ , which at low field is hundreds of s^–1^, as experimentally observed.^54,55^ This implies that only a small percent of bound ligand must be present in the solution to allow for accurate measurements. In the present conditions, the amount of bound protein is 99.5%, 66.5%, 16.6% and 2.0% for dissociation constants of 10^–6^, 10^–4^, 10^–3^ and 10^–2^, respectively, while the amount of bound ligand (*c*_bound_) varies from 0.995% to 0.020%.

**Figure 2:**
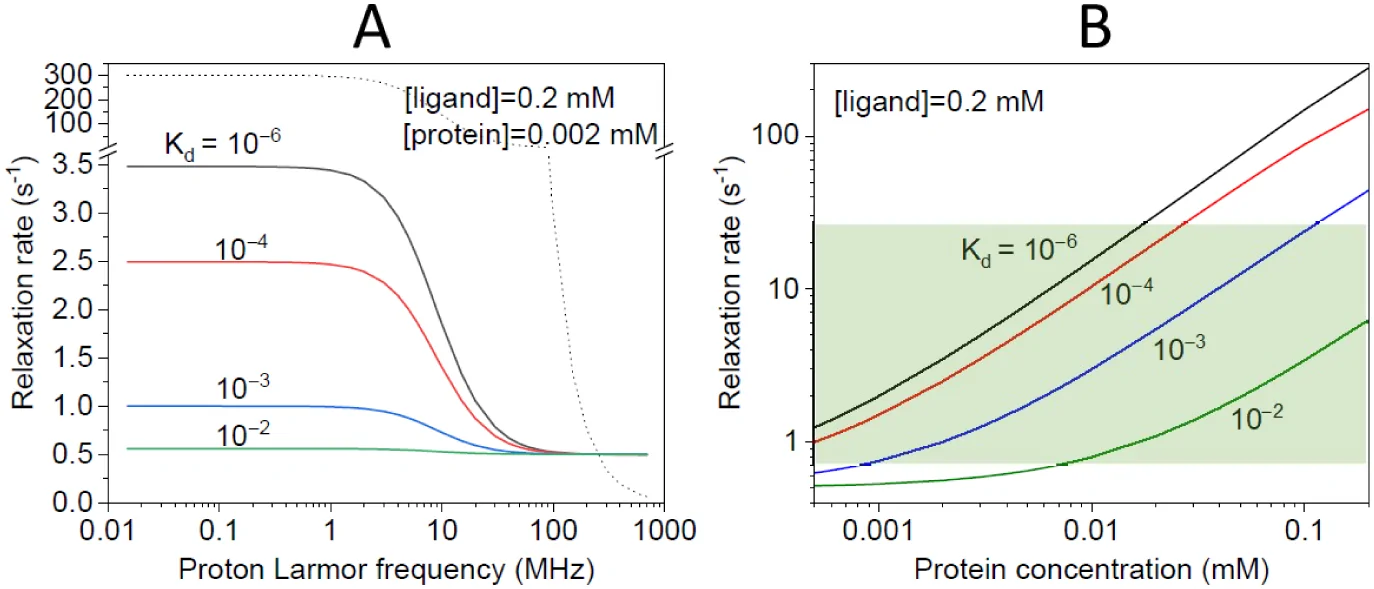
Field dependence of the relaxation rate of ligand protons calculated using eqs 1–3 (*c*_protein_ = 2 μM) for different values of the dissociation constant; the dotted line indicates the $R_{1}^{\mathrm{bound}}$ values (A). Low-field relaxation rate of ligand protons as a function of the protein concentration, *c*_protein_, for different values of the dissociation constant ( $R_{1}^{\mathrm{free}}$ = 0.5 s^–1^); the green zone indicates the $R_{1}^{\mathrm{ligand}}$ values detectable with the available shuttle system and significantly larger than $R_{1}^{\mathrm{free}}$ (B). Conditions: *τ_c_* = 10 ns, *c*_ligand_ = 0.2 mM, *r* = 1.75 Å, and assuming fast exchange between the free and bound forms of the ligand.

Figure 2 also shows the ligand proton relaxation rates at low fields (below 1 MHz proton Larmor frequency) as a function of the protein concentration, all other conditions being the same. The figure indicates the range of protein concentrations, and its variability depending on the dissociation constant, providing $R_{1}^{\mathrm{ligand}}$ values detectable with the available shuttle system (up to few tens of s^–1^) and significantly larger than $R_{1}^{\mathrm{free}}$ at low magnetic fields, so that the occurrence of binding can be appreciated. Note that smaller correlation times for the bound ligand (i.e., smaller proteins) and/or larger proton–proton distances cause a decrease in $R_{1}^{\mathrm{bound}}$ which results in a decrease of the $R_{1}^{\mathrm{ligand}}$ values. This moves toward higher values the range of protein concentrations providing detectable $R_{1}^{\mathrm{ligand}}$ . Conversely, larger proteins require a smaller concentration.

Finally, Figure 3 shows the simulated ligand proton relaxation rates at low fields (0.01 MHz) as a function of its overall reorientation time and lifetime in the bound position, calculated assuming 1% of the ligand in bound state, and *r* = 1.75 Å (as in “isolated” methylene groups, left panel) or 2.50 Å (as in the aromatic rings of the considered fragments, right panel). The figure indicates that in these conditions the available shuttle system can allow for the detection of ligand binding to macromolecules with reorientation times in the range from 10 to 100 ns in the first case and up to 1000 ns in the second case.

**Figure 3:**
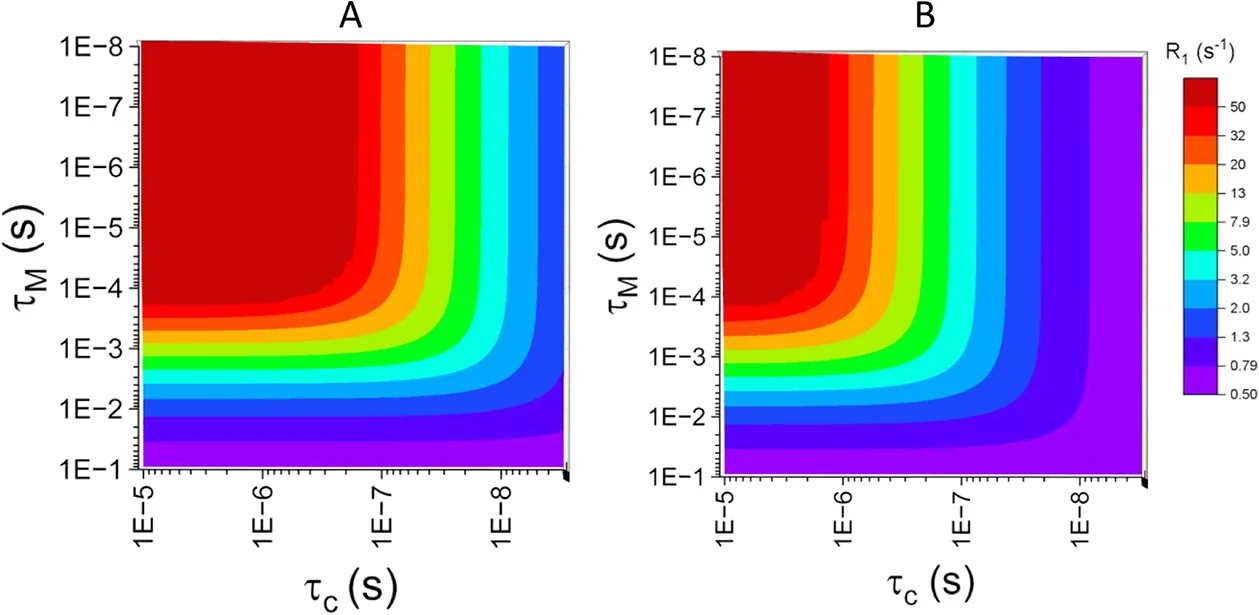
Low-field (0.01 MHz) relaxation rate of ligand protons as a function of the reorientation time *τ_c_* and lifetime *τ_M_*, for an interproton distance *r* of 1.75 Å (A) or 2.50 Å (B), assuming 1% of the ligand in the bound state.

## Results and Discussion

High-resolution relaxometry measurements were performed for solutions of the catalytic domain of MMP-12 with AHA and, as ligands, each of the fragments shown in Figure 1. The concentrations of all species are reported in Table 1. As described above, the fragment concentrations were chosen in large excess with respect to the protein. Although the vast majority of the molecules is thus in the unbound form, the small fraction of the fragments bound to the protein $\left(\frac{c_{\mathrm{bound}}}{c_{\mathrm{ligand}}}\right)$ and in exchange with the free form can act as a reporter of their interaction.

**Table 1:** Experimental Conditions and Best Fit Parameters of the Field-Dependent Relaxation Rates of Fragment Protons for Solutions of the Catalytic Domain of MMP-12 with the Different Fragments

Fragment	*c*_ligand_ (mM)	*c*_protein_ (mM)	*τ_c_* (ns)	*a* $\left[\approx R_{1}^{\mathrm{free}}\right]$ (s^–1^)	*b* (s^–2^)
BPN	0.2	0.002	7.9 ± 0.4	0.75 ± 0.02	(2.5 ± 0.1) × 10^8^
MLC	0.2	0.01	7.3 ± 0.7	0.58 ± 0.02	(1.1 ± 0.1) × 10^8^
MBS	0.8	0.02	7.8 ± 0.5	0.48 ± 0.02	(1.6 ± 0.1) × 10^8^

The experiments were acquired at 10 relaxation magnetic fields (from 40 mT to 14.1 T, with proton Larmor frequencies ranging from approximately 2 to 600 MHz) using six to eight relaxation delays. The processed spectra showed a progressive decrease in signal intensity as a function of the relaxation delay at low field (Figure 4). Analogous experiments were performed for the fragments in the same buffer, in the absence of the protein. The spectra clearly show a more pronounced loss of signal intensity in the presence of the protein, indicating the occurrence of protein-fragment interactions.

**Figure 4:**
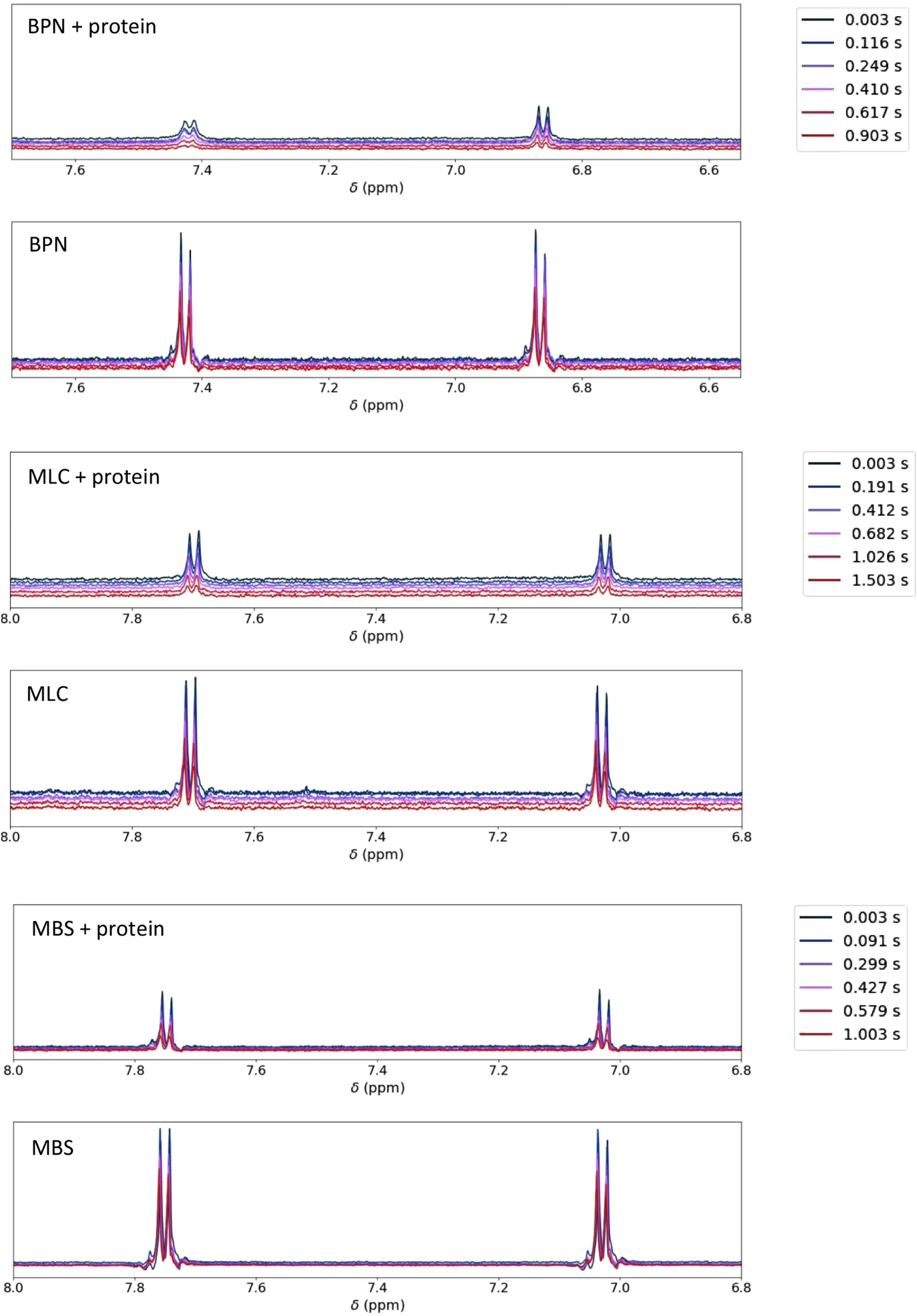
Signal decays of the aromatic protons of the fragments in the presence and absence of the protein, recorded at 600 MHz after the six indicated relaxation delays at 10 MHz. The faster decay observed in the presence of the protein reflects enhanced relaxation due to binding. The plot was generated using KLASSEZ.^61,62^

At each magnetic field, the signal intensities of the fragment protons as a function of the relaxation delay were fitted with a monoexponential decay function in order to obtain the longitudinal relaxation rates. The fits were of high quality, and the averaged relaxation rates obtained for the four peaks of each fragment are shown in Figure 5. As expected, the free fragments exhibited nearly field-independent NMRD profiles, whereas the presence of the protein induced a clear low-field dispersion. By extending relaxation measurements into this low-field regime, where the difference between the free and bound forms is greatest, HRR provides a remarkable gain in sensitivity for detecting, even weak, binding interactions.

**Figure 5:**
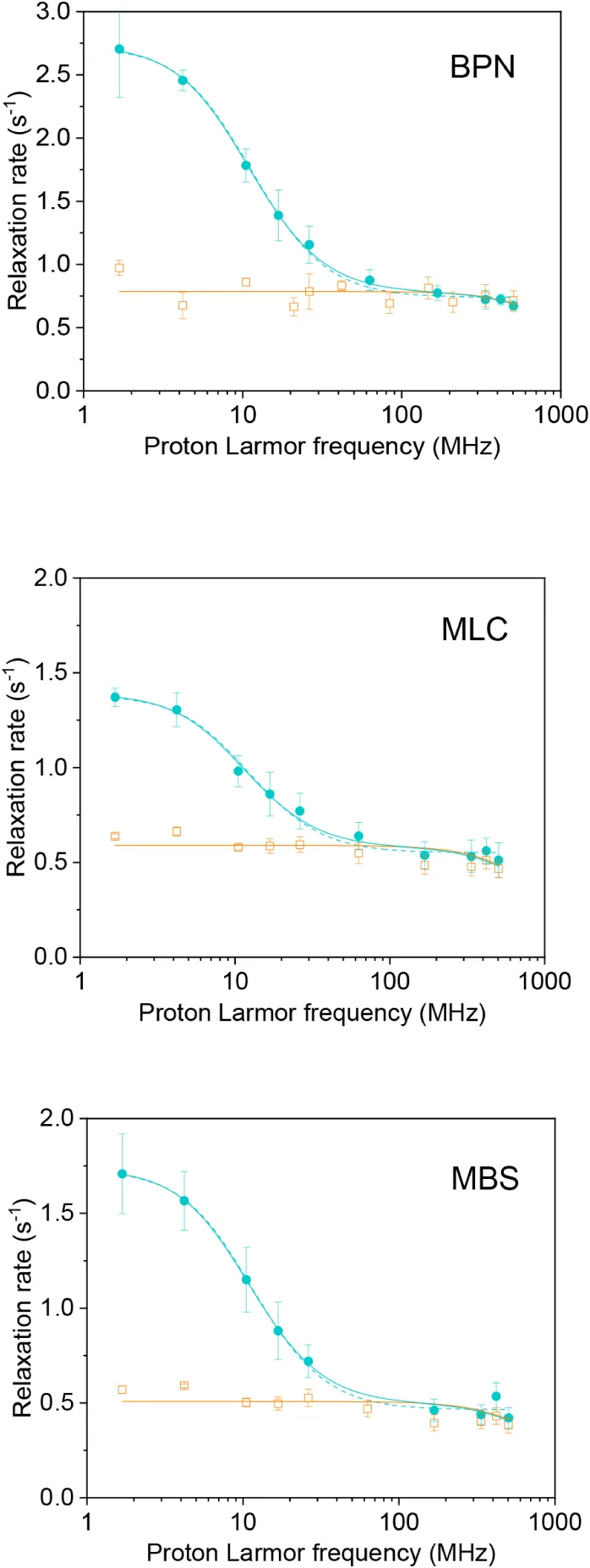
Relaxation profiles of the aromatic protons of the fragments in the presence (blue) and in the absence (orange) of the catalytic domain of MMP-12. Best fit profiles of the relaxation rates obtained with eq 5 are also shown as solid line, whereas dashed lines represent the fits obtained with eq 4.

The relaxation profiles obtained for the fragment protons in the presence of the protein were fitted to eq 4, resulting from eq 1 and eq 2 in the assumption of fast exchange between bound and free ligand conditions,

4 $$R_{1}^{\mathrm{ligand}}=a+b\left[\frac{0.2\tau_{c}}{1+\omega_{I}^{2}\tau_{c}^{2}}+\frac{0.8\tau_{c}}{1+4\omega_{I}^{2}\tau_{c}^{2}}\right]$$

The fit parameters were *a*, *b* and the correlation time *τ_c_*. The best fit values are reported in Table 1. The best fit profiles are shown in Figure 5 as dashed lines. Notably, a correlation time of ca. 8 ns is obtained from all profiles, in nice agreement with the reorientation time of the catalytic domain of MMP-12 as can be calculated with HydroNMR^56^ (equal to 8.9 ns), where a completely rigid structure is assumed.

Under the approximation that the concentration of the protein-bound fragment is equal to the protein concentration (the protein is expected to be saturated as long as the ligand concentration is much larger than the dissociation constant of the complex), the value of the parameter *a* corresponds to $\frac{c_{\mathrm{free}}}{c_{\mathrm{ligand}}}R_{1}^{\mathrm{free}}\approx R_{1}^{\mathrm{free}}$ (since the protein concentration is much smaller than the ligand concentration), and the value of the parameter *b* corresponds to $\frac{c_{\mathrm{protein}}}{c_{\mathrm{ligand}}}\frac{3}{2}{\left(\frac{\mu_{0}}{4\pi }\frac{\hbar \gamma_{I}^{2}}{r^{3}}\right)}^{2}$ . Using a simplified model that does not explicitly include intermolecular interactions, the best fit values of *b* were used to calculate the “effective” distances *r* between each ligand proton and the dipolarly coupled proton(s), resulting in 1.8, 2.7, and 2.3 Å for BPN, MLC and MBS, respectively. Some of these values are significantly shorter than the interproton distance of 2.5 Å expected for the two closest protons in the aromatic rings of the fragments. The origin of this discrepancy is most likely ascribed to the proximity of protein protons and/or additional intramolecular dipolar interactions, as will be detailed below.

In order to better refine the data analysis, we experimentally determined the dissociation constant of MMP-12 with each ligand/fragment by protein-detected NMR titration in the presence of 0.2 M AHA. Dissociation constants were measured using a 500 MHz Bruker UltraShield spectrometer at 25 °C. The resulting values, reported in Table 2, were used to determine the protein-bound fraction of the ligand to be used in the fit.

**Table 2:** Conditional Dissociation Constants, and Best Fit Parameters of the Field-Dependent Relaxation Rates of Fragment Protons for Solutions of the Catalytic Domain of MMP-12 with the Different Fragments

Fragment	*K*_*D*_ ^a^	$\frac{c_{\mathrm{bound}}}{c_{\mathrm{ligand}}}$	$\frac{c_{\mathrm{bound}}}{c_{\mathrm{protein}}}$	τ*_f_* (ps)	β_*free*_ (s^–2^)	*τ_c_* (ns)	β (s^–2^)
BPN	(2.0 ± 1.0) × 10^–6^	0.99%	99.0%	64 ± 20	(1.2 ± 0.3) × 10^10^	8.2 ± 0.6	(2.4 ± 0.3) × 10^10^
MLC	(4.3 ± 0.6) × 10^–4^	1.57%	31.4%	80 ± 14	(7.3 ± 0.8) × 10^9^	7.8 ± 0.8	(6.6 ± 0.8) × 10^9^
MBS	(3.3 ± 0.2) × 10^–4^	1.76%	70.4%	84 ± 18	(6.1 ± 1.2) × 10^9^	8.2 ± 0.7	(8.6 ± 1.2) × 10^9^

a The dissociation constants are termed conditional to account for the fact that the binding is always in the presence of AHA, whose role could range from noncompetitive to partially competitive.

The relaxation profiles of the fragment without and with the protein in solution were then fitted jointly to eq 5

5 $$R_{1}^{\mathrm{ligand}}=\frac{c_{\mathrm{free}}}{c_{\mathrm{ligand}}}\beta_{free}\left[\frac{0.2\tau_{f}}{1+\omega_{I}^{2}\tau_{f}^{2}}+\frac{0.8\tau_{f}}{1+4\omega_{I}^{2}\tau_{f}^{2}}\right]+\frac{c_{\mathrm{bound}}}{c_{\mathrm{ligand}}}\beta \left[\frac{0.2\tau_{c}}{1+\omega_{I}^{2}\tau_{c}^{2}}+\frac{0.8\tau_{c}}{1+4\omega_{I}^{2}\tau_{c}^{2}}\right]$$

where *β_free_* and *β* are the squared dipolar interaction constants (corresponding to $\frac{3}{2}{\left(\frac{\mu_{0}}{4\pi }\frac{\hbar \gamma_{I}^{2}}{r^{3}}\right)}^{2}$ ) of the ligand protons in the free and bound forms, respectively, *c*_bound_ is equal to zero in the absence of the protein and equal to the value obtained from eq 3 in the presence of the protein, and *τ_f_* corresponds to the correlation time of the free fragment. The best-fit profiles are shown as solid lines in Figure 5. The quality of the fits is very good, similar to those previously obtained (in terms of the residual sum of squares), and the optimized values of the fit parameters, *β_free_*, *τ_f_*, *β* and *τ_c_*, are reported in Table 2. Crucially, *τ_c_* values of 7.8–8.2 ns are obtained, consistently matching the reorientation time of the protein previously determined experimentally (8.7 ns),^57^ somewhat smaller than the value of 8.9 ns calculated under the assumption of a fully rigid structure. This close agreement thereby provides robust evidence for the formation of the protein-fragment complex.

The reorientation times for the free fragments, ranging from 60 to 90 ps, are consistent with the values expected based on their sizes, notwithstanding the large covariance between *β*_*free*_ and *τ_f_*. The values of *β* and *β_free_* expected for the interproton distance of 2.5 Å, as that of the two closest protons in the aromatic rings of the fragments, is 3.5 × 10^9^ s^–2^. Consequently, the expected relaxation rates for the free fragments should amount to approximately 0.25–0.30 s^–1^, which is significantly lower than the experimental values. The additional contribution to the relaxation rates (ranging from 0.4 for BPN to 0.2 for MBS) can be reasonably attributed to the presence of dissolved oxygen.^58^

While the experimental values of *β* and *β*_*free*_ are similar for MLC and MBS, in the case of BPN *β* is twice the value of *β*_*free*_. Values of *β* higher than expected suggest that upon binding to the protein, additional dipolar contributions arise for the fragment protons due to their interactions with protein protons. For the MLC and MBS, these values suggest the presence of an additional protein proton at a distance of 2.3–2.5 Å. In the case of BPN, a larger contribution to *β* could arise from a higher inter-ring proton–proton contribution, which occurs when the two rings adopt a nearly coplanar conformation, as observed in a structure with a similar biphenyl moiety (PDB 1ROS).

In the present analysis, cross-relaxation effects, arising from either autocorrelated dipole–dipole interactions or cross-correlated mechanisms, have been neglected.^59^ Advanced analysis of HRR data in proteins has shown that cross-relaxation can introduce small discrepancies between the rates obtained from the observed magnetization decays and the intrinsic longitudinal relaxation rates. Indeed, signal decays recorded with HRR can be affected by cross-relaxation pathways because radiofrequency pulses cannot be applied while the sample is outside the probe for low-field relaxation and during the transfers between high- and low-field positions.^60^ In the present system, as with the previously reported case of olive oil,^17^ such deviations are expected to be minimal, representing only a small fraction of the nuclear relaxation rates, and thus not able to substantially alter the overall results. Furthermore, since cross-relaxation leads to a decrease in the observed rates of the magnetization decays, neglecting these effects should result in slightly overestimating internuclear distances. Therefore, cross-relaxation cannot account for the shorter interproton distances obtained in the current analysis.

The methodology presented here requires access to a new class of instruments, currently available in about half a dozen laboratories worldwide, such as the newly developed fast sample shuttle system (FSS). With the current prototypes, the method is limited to targets with molecular weights below approximately 20 kDa. However, a next-generation FSS system, currently under development, will extend this range to biological targets with molecular weights up to 2 MDa. While STD-NMR and WaterLOGSY are widely used to detect binding events through magnetization transfer, they are inherently prone to specific artifacts that high-resolution relaxometry intrinsically overcomes. In contrast to these methods, HRR indeed directly provides a highly specific physical descriptor of the interaction: the rotational correlation time of the bound species. This parameter enables unequivocal verification that the fragment tumbles at the same rate as the macromolecule in the complex, thereby providing strong evidence for a true binding event and effectively ruling out false positives arising from nonspecific fragment aggregation. A further important advantage of HRR lies in the ability to operate over a wide dynamic range of concentrations. Traditional screening techniques often struggle with poorly soluble fragments, as lowering ligand concentrations dampens the signal-to-noise ratio or suppresses the magnetization transfer efficiency. In contrast, low-field operating HRR exploit a region where the difference between the relaxation rates of the free and bound forms is maximized, resulting in a massively enhanced sensitivity. Consequently, successful binding detection can be achieved even at very low ligand concentrations, offering a powerful alternative for screening low-solubility fragments. Realistically, although this approach is expected to have lower throughput compared to other NMR-based techniques, its greatest potential and future utility lies in its use as a secondary screening tool, particularly for challenging fragment libraries containing sparingly soluble fragments.

## Conclusions

The ^1^H longitudinal relaxation dispersion profiles measured for three small fragments demonstrate that protein-fragment binding can be detected for a 17 kDa target protein, the catalytic domain of MMP-12, using protein concentrations as low as 2–20 μM and fragment concentrations only 20–100-fold higher, for dissociation constants in the 10^–6^–10^–4^ range. The NMRD profiles were acquired over nearly 3 orders of magnitude of magnetic fields (0.04–14 T), corresponding to the range between 2 and 600 MHz proton Larmor frequency, using a fast sample shuttle system installed on a standard 600 MHz spectrometer.

The observed dispersion provides a reorientation time of ca. 8 ns for the protein-fragment complex, in agreement with the value expected for a protein of this size. Beyond revealing fragment relaxation changes arising from interaction with the macromolecular system, HRR enables a direct assessment of genuine target engagement through the reorientation correlation time as a physical descriptor. When the expected rotational correlation time of the target protein is known, this parameter offers an intrinsic validation criterion to distinguish specific binding from artifactual relaxation increases caused by nonspecific interactions and fragment aggregation, providing an additional level of confidence in target engagement and potentially eliminating the need for additional experiments. Moreover, the ability to operate at low ligand concentrations makes this approach particularly valuable for detecting interactions with poorly soluble compounds. Overall, high-resolution relaxometry emerges as a powerful and efficient approach for fragment screening or hit validation in drug discovery and pharmaceutical research, combining minimal sample consumption with detailed information on the complex reorientation dynamics.

## Experimental Section

### Materials

All fragments were purchased from Sigma-Aldrich (purity >95%). The ^15^N labeled MMP12 (F171D) catalytic domain used in HHR measurements was purchased from GIOTTO BIOTECH (CAT # G04MP12Cm). The experimental buffer was 20 mM Tris pH 7.2, 10 mM CaCl_2_, 0.1 mM ZnCl_2_, 0.3 M NaCl, 0.2 M AHA. This research did not involve human or animal participants.

### High-Resolution Relaxometry

High-resolution relaxometry measurements were performed at 25 °C with a Bruker Avance III spectrometer operating at 600 MHz (∼14.1 T), equipped with a 5 mm PH TXI 600S3 H-C/N-D-05 BTO probe with XYZ gradients, and with the prototype of the fast sample shuttle (FSS) system.^16^ The sample was contained in a purposely designed 5 mm tube with sample volume of 650 μL. The FSS consists of a drive unit that is connected through a cord with the sample container and moves it into the stray field of the magnet. A continuous high pressure (4.5 bar) air flow pushed the sample container tube inside the magnet to the high-field detection position and counteracted the action of the cord. For an exhaustive description of the hardware, the reader is referred to reference.^16^

The employed pulse sequence is depicted and described in Figure 6.

**Figure 6:**
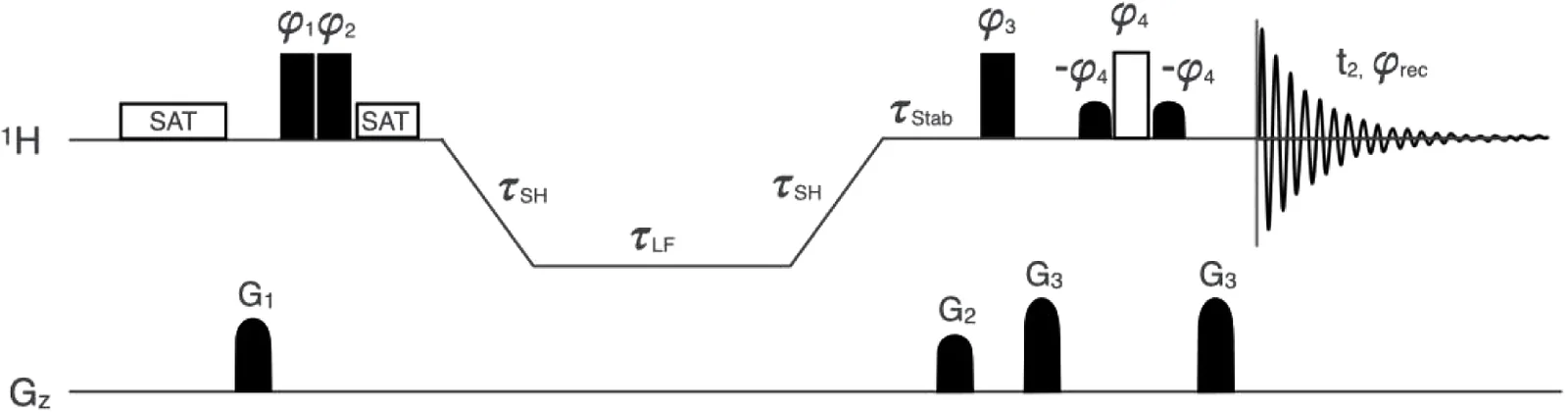
Graphical representation of the employed pulse sequence. Black and white rectangles represent 90° and 180° pulses, respectively. Phase cycles are as follows: φ_1_ = 8*­(x, −x), φ_2_ = 8*­(x), 8*­(−x), φ_3_ = 2*­(x, x, −x, −x, y, y, −y, −y), φ_4_ = 2*­(y, y, −y, −y, −x, −x, x, x), φ_rec_ = x, −x, −x, x, y, −y, −y, y, −x, x, x, −x, −y, y, y, −y. Before and after the first two 90° pulses, a continuous wave pulse for water saturation (SAT) is applied for 1 s and 10 ms, respectively. The travel time between high and low field, τ_SH_, was set to be equal to 61 ms in both directions with symmetrical constant acceleration and deceleration, regardless the relaxation field. τ_LF_ represents a series of a variable number (between 6 and 12) of relaxation delays at low field. The longest delay of each experiment was chosen to correspond to a maximum of 70% signal loss compared to the one with zero delay, and the increments were logarithmically spaced between these two values. A stabilization delay of 150 ms, τ_Stab_, after shuttling back to high field was employed to reduce vibration artifacts. Gradient pulses are indicated as black bell shapes on the gradient channel, G. The smallest gradient pulse after shuttling back to high field was kept for similarity with the original static sequence. All gradients were smoothed squared (SMSQ10.100), applied for 1 ms on the *z* axis, with the following powers with amplitudes of 33.5, −6.7, 22.78 G/cm, for G_1_, G_2_, G_3_ respectively. A WATERGATE block after the last 90° pulse was added to ensure water suppression.^64^

The spectra were processed using the Bruker TOPSPIN 4.0 software package. The employed window function was a squared cosine, a zero-order and first-order phase correction was applied manually on the pseudo 2D spectrum, and an automatic baseline correction was applied from 5 to 10 ppm subtracting a polynomial function of degree 5.

To obtain the longitudinal relaxation times of each doublet of the aromatic protons of the fragments, the series of pseudo 2D spectra recorded at each field was analyzed performing: (i) alignment of the 1D spectra taking the TRIS peak between around 3.7 and 3.5 ppm as calibration peak; (ii) fits of each doublet using Voigt functions; (iii) monoexponential fits of the decay of the amplitude of each doublet. Biexponential fits were also performed, which provided larger or similar reduced χ^2^ values. They were thus excluded because they introduced additional parameters without significantly improving the quality of the fit. All employed functions and a tutorial on how to use them are available in ref 63. Examples of these fits are shown in Figure 7 and Figures S1–S18.

**Figure 7:**
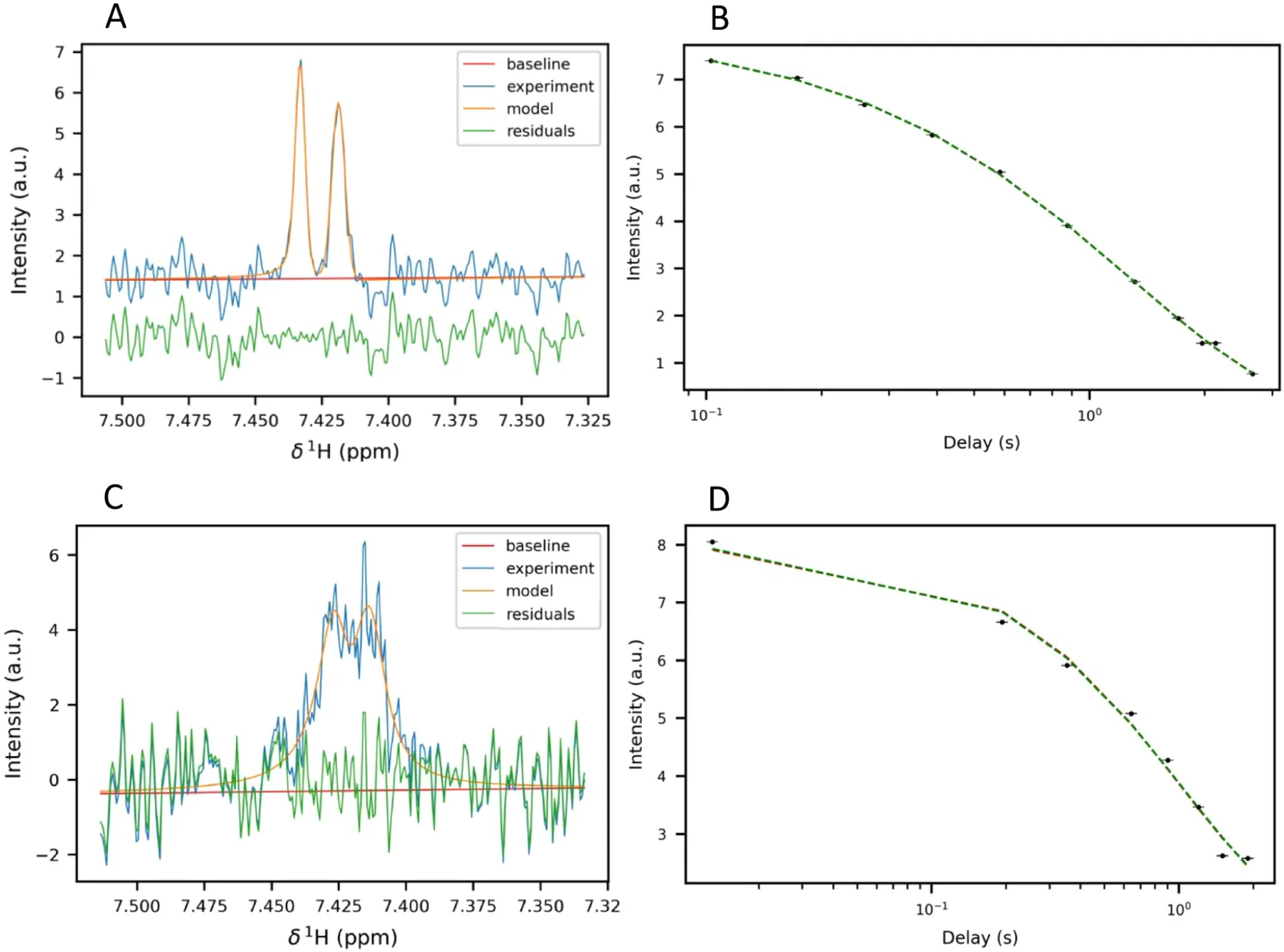
Experimental and fitted NMR doublets of the BPN sample in the absence (A) and presence (C) of the MMP-12 catalytic domain, acquired with the longest relaxation delay at 250 mT/10 MHz. Panels (B) and (D) report the derived complete fitted amplitude decays as a function of the relaxation delay.

### Dissociation Constants Measurements

Dissociation constants were measured via protein-detected NMR titration using a 500 MHz Bruker UltraShield AVANCE NEO spectrometer, equipped with a quadrupole resonance QCI H/P/C/N Cryoprobe, at 25 °C. The effect of fragments was evaluated on ^15^N-isotopically enriched MMP-12 protein at concentrations ranging between 0.1 and 0.2 mM in the following buffer conditions: 20 mM Tris (pH 7.2), 10 mM CaCl_2_, 0.1 mM ZnCl_2_, 0.3 M NaCl, 0.2 M AHA. During the NMR titrations, increasing amounts of the fragments, solubilized in DMSO-d6, were added to the protein solution and 2D ^1^H–^15^N HSQC spectra were recorded. The effect of DMSO-d6 on the catalytic domain of MMP-12 was previously observed to be negligible at the employed concentrations. The protein concentration and the fragment aliquots were adjusted according to the known literature values of *K*_*D*_ for the selected fragments (final concentrations reached in solution were 0.05, 0.1, 0.2, 0.4, 0.8 and 1.6 mM for MBS and MLC, with 0.2 mM protein concentration, and 0.0125, 0.025, 0.050, 0.1, 0.2, and 0.4 mM for BPN, with 0.1 mM protein concentration). Spectra were processed using the Bruker TOPSPIN 4.0 software package and analyzed with CARA (ETH Zurich). Signal assignment relied on previously reported literature data for MMP12 (BMRB code: 6444^22^). Chemical shift perturbations (CSP) were evaluated using the formula^65^ $\Delta \delta =\frac{1}{2}\sqrt{\Delta \delta_{H}^{2}+{\left(\Delta \delta_{N}/5\right)}^{2}}$ and the *K*_*D*_ was obtained by fitting the largest CSP against fragment concentration, using the formula^66^

$$\Delta \delta_{obs}=\Delta \delta_{max}\left\{\left(c_{\mathrm{protein}}+c_{\mathrm{ligand}}+K_{D}\right)-{[{\left(c_{\mathrm{protein}}+c_{\mathrm{ligand}}+K_{D}\right)}^{2}-4c_{\mathrm{protein}}c_{\mathrm{ligand}}]}^{1/2}\right\}/\left(2c_{\mathrm{protein}}\right)$$

in OriginPro 9 software.

## Supplementary Material





## Notes

Molecular formula strings data (CSV)
Figures S1–S18: experimental and fitted NMR doublets and derived amplitude decays as a function of the relaxation delay (PDF)

## Abbreviations:

AHA: acetohydroxamic acid
BPN: biphenol
FBDD: fragment-based drug discovery
FFC: fast field-cycling
FSS: fast sample shuttle
HRR: high-resolution relaxometry
MBS: 4-methoxybenzenesulfonamide
MLC: 4-methoxybenzenesulfonamide glycine
MMP: matrix metalloproteinase
NMR: nuclear magnetic resonance
NMRD: nuclear magnetic resonance dispersion
PAINS: pan-assay interference compounds
STD: saturation transfer difference
WaterLOGSY: water-ligand observed via gradient spectroscopy
