# Supplementary material for: High-Resolution Relaxometry for Fragment Screening
Source: J Med Chem. 2026 Aug 12;69(16):19971–81. doi: 10.1021/acs.jmedchem.6c01773 (PMC13528194; doi:10.1021/acs.jmedchem.6c01773)
Supplement: Supplementary file 1 [file jm6c01773_si_002.pdf]

SUPPORTING INFORMATION

High-Resolution Relaxometry for Fragment Screening

Giulia Licciardi,[1,2,3] Linda Cerofolini,[1,2] Ulric Le Paige,[3] Adam Kubrak,[1,2] Letizia Fiorucci,[1,2] Luis Padilla-Cortés,[1,2] Enrico Ravera,[1,2] Marco Fragai,[1,2] Claudio Luchinat,[1,2] Fabien Ferrage,[3] Giacomo Parigi[1,2*]

[1]Department of Chemistry "Ugo Schiff" and Magnetic Resonance Center (CERM), University of Florence, Sesto Fiorentino, 50019 Italy

[2]Consorzio Interuniversitario Risonanze Magnetiche MetalloProteine (CIRMMP), via Sacconi 6, Sesto Fiorentino, 50019 Italy

[3]Laboratoire des Biomolécules, LBM, Département de chimie, École normale supérieure, PSL University, Sorbonne Université, CNRS, 75005 Paris, France

*giacomo.parigi@unifi.it

Contents:

Figures S1-S6: Experimental and fitted NMR doublets of the BPN ligand…………………..S2

Figures S7-S12: Experimental and fitted NMR doublets of the MBS ligand…………..……..S15

Figures S13-S18: Experimental and fitted NMR doublets of the MCL ligand……………….S27

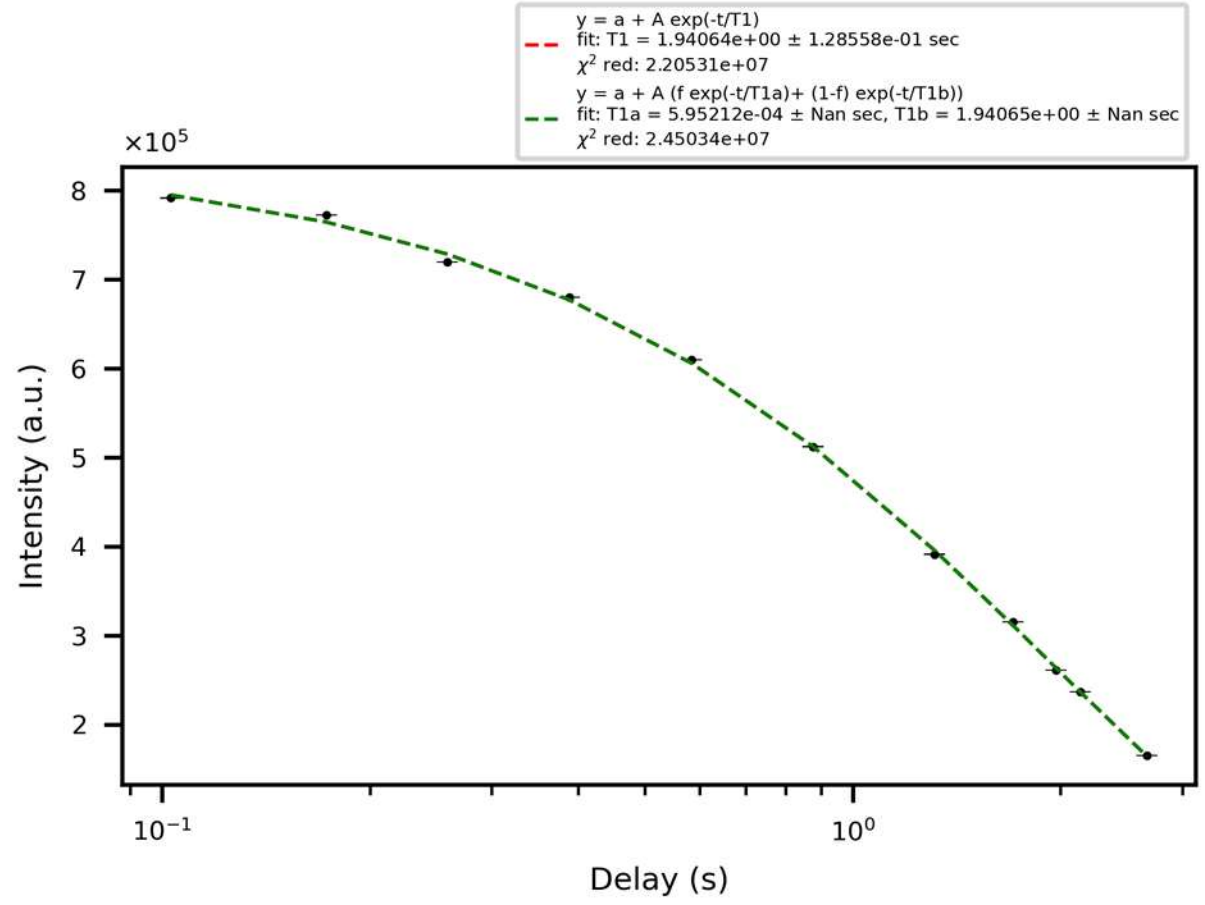
y = a + A exp(-t/T1)
fit: T1 = 1.94064e+00 ± 1.28558e-01 sec
χ² red: 2.20531e+07
y = a + A (f exp(-t/T1a)+ (1-f) exp(-t/T1b))
fit: T1a = 5.95212e-04 ± Nan sec, T1b = 1.94065e+00 ± Nan sec
χ² red: 2.45034e+07
×10⁵
Intensity (a.u.)
Delay (s)

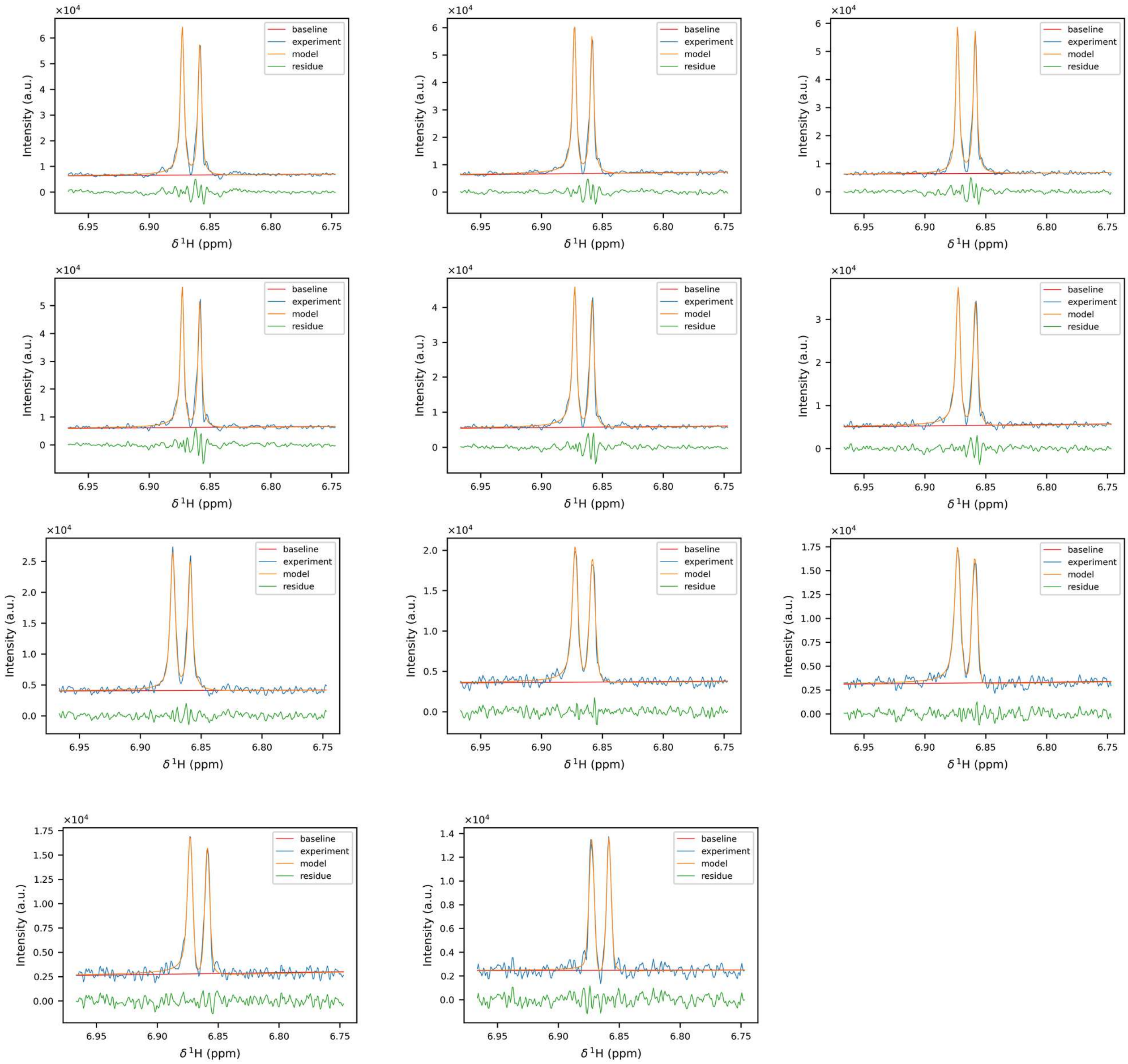
baseline
experiment
model
residue
Intensity (a.u.)
δ ¹H (ppm)

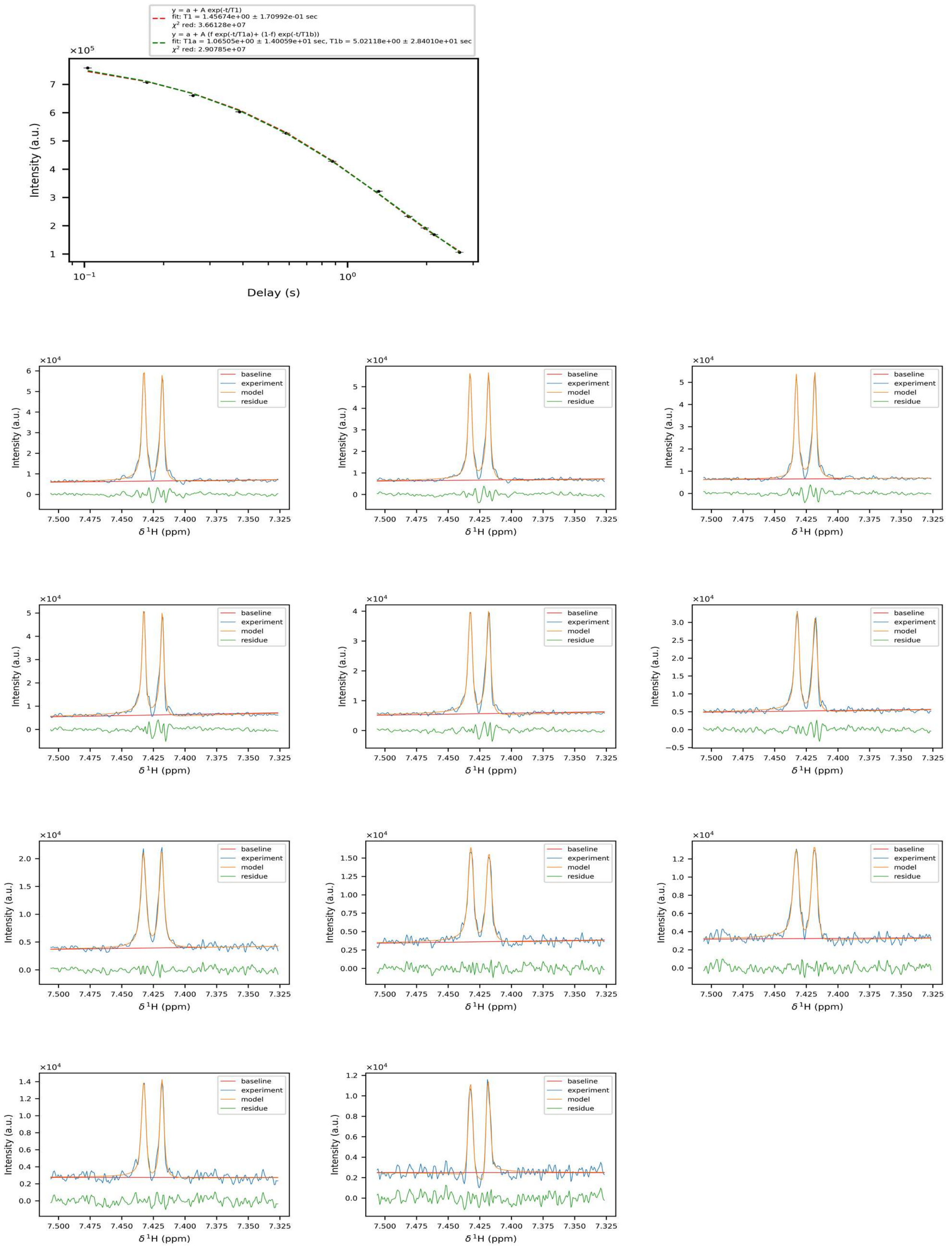

Figure S1: Experimental and fitted NMR doublets of the BPN ligand in the absence of the MMP-12 catalytic domain, acquired at 11 interleaved relaxation delays (0.1, 1.967, 1.311, 0.170, 0.873, 0.256, 0.581, 1.705, 2.131, 0.386, 2.664 s, here displayed from shortest to longest) at 8000 mT/336 MHz. The first panels report the derived complete fitted amplitude decays as a function of the relaxation delay.

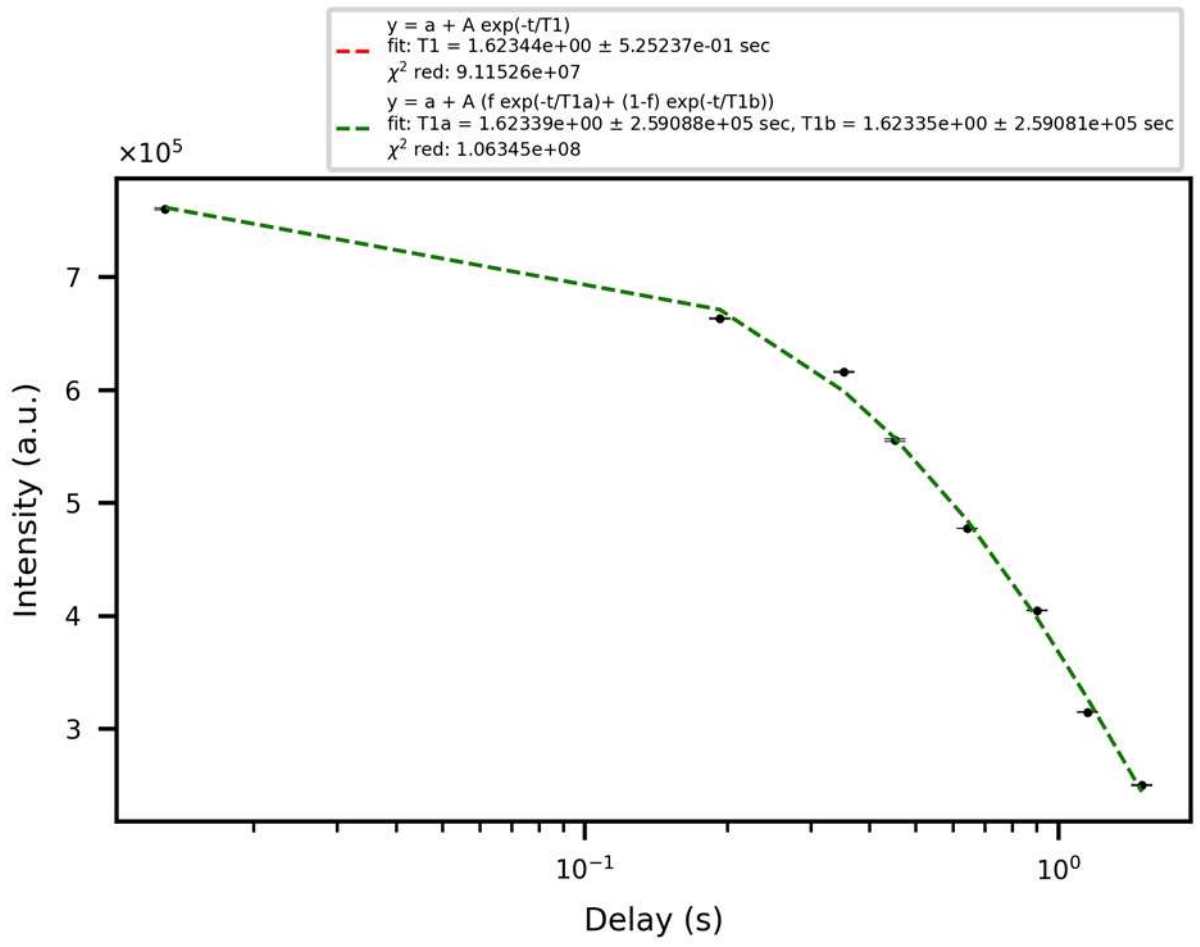

y = a + A exp(-t/T1)
fit: T1 = 1.62344e+00 ± 5.25237e-01 sec
χ² red: 9.11526e+07
y = a + A (f exp(-t/T1a)+ (1-f) exp(-t/T1b))
fit: T1a = 1.62339e+00 ± 2.59088e+05 sec, T1b = 1.62335e+00 ± 2.59081e+05 sec
χ² red: 1.06345e+08
×10⁵
Intensity (a.u.)
Delay (s)

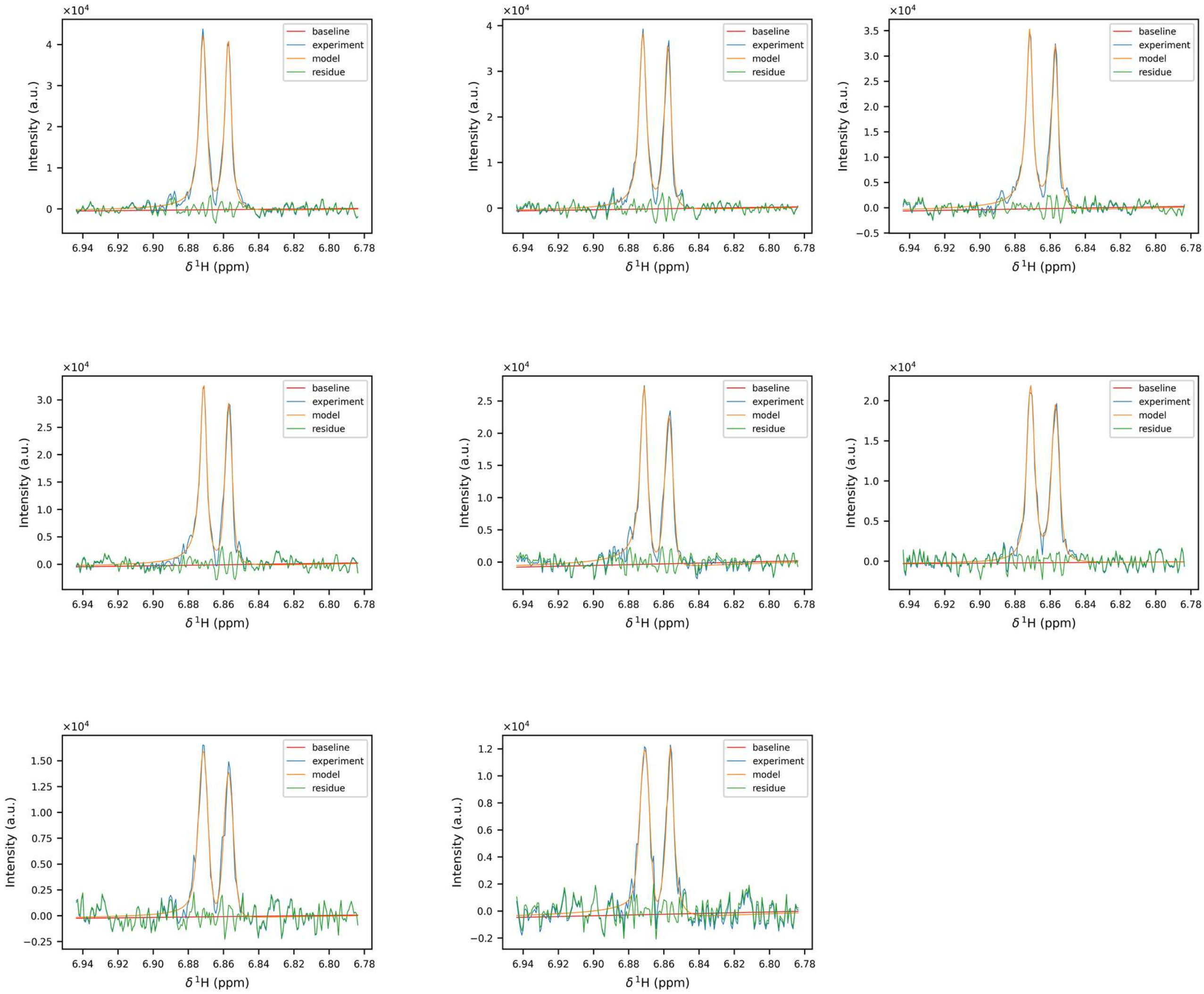

baseline
experiment
model
residue
Intensity (a.u.)
δ ¹H (ppm)

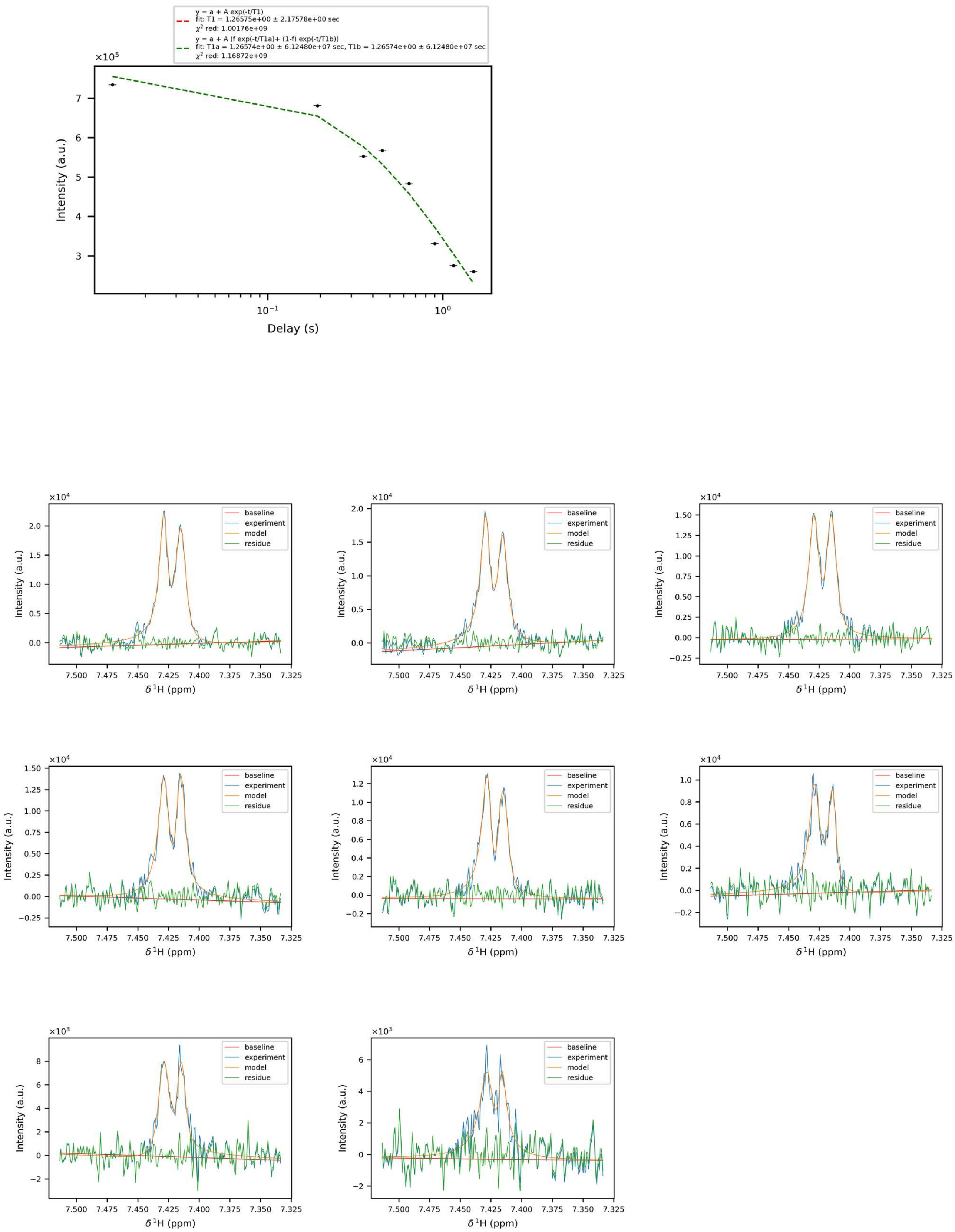

Figure S2: Experimental and fitted NMR doublets of the BPN ligand in the presence of the MMP-12 catalytic domain, acquired at 8 interleaved relaxation delays (0.01, 1.5, 0.64, 1.15, 0.19, 0.35, 0.9, 0.45 s, here displayed from shortest to longest) at 8000 mT/336 MHz. The first panels report the derived complete fitted amplitude decays as a function of the relaxation delay.

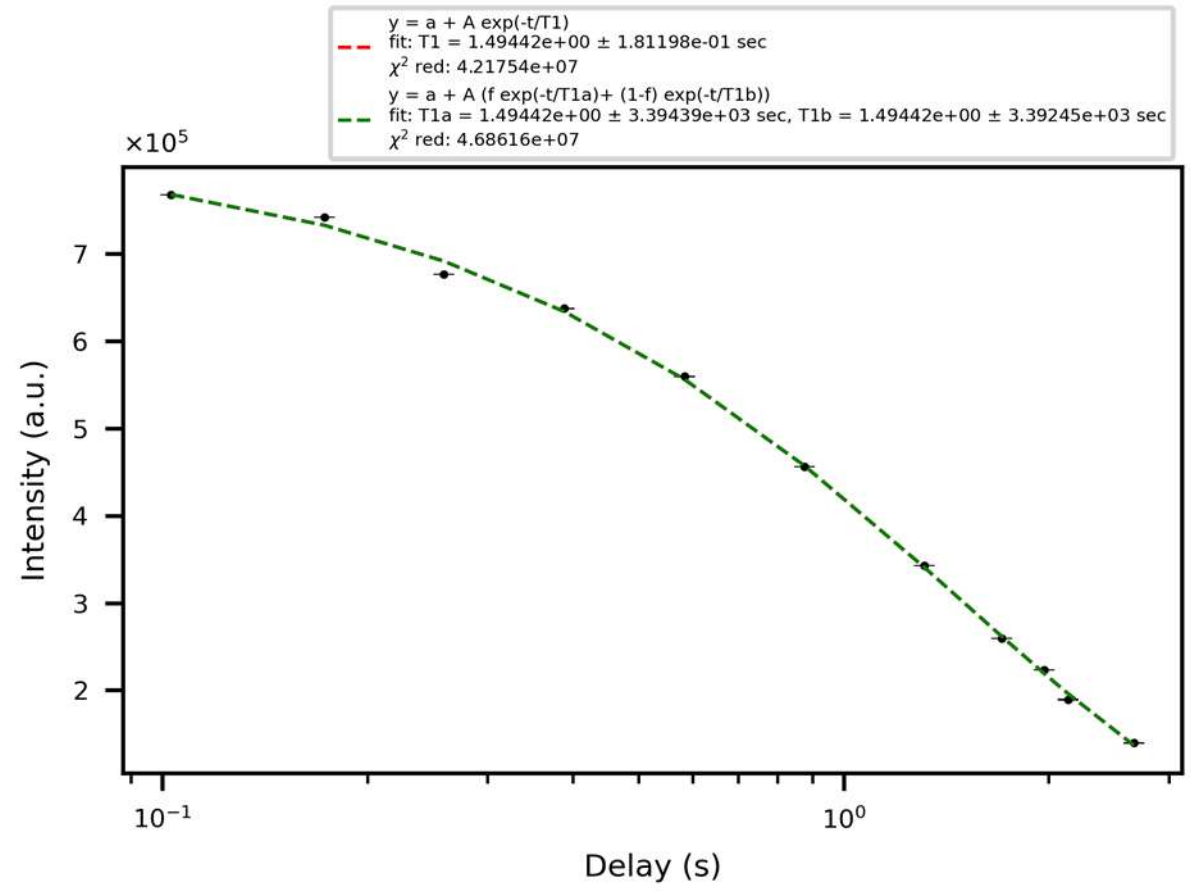
y = a + A exp(-t/T1)
fit: T1 = 1.49442e+00 ± 1.81198e-01 sec
χ² red: 4.21754e+07
y = a + A (f exp(-t/T1a)+ (1-f) exp(-t/T1b))
fit: T1a = 1.49442e+00 ± 3.39439e+03 sec, T1b = 1.49442e+00 ± 3.39245e+03 sec
χ² red: 4.68616e+07
×10⁵
Intensity (a.u.)
Delay (s)

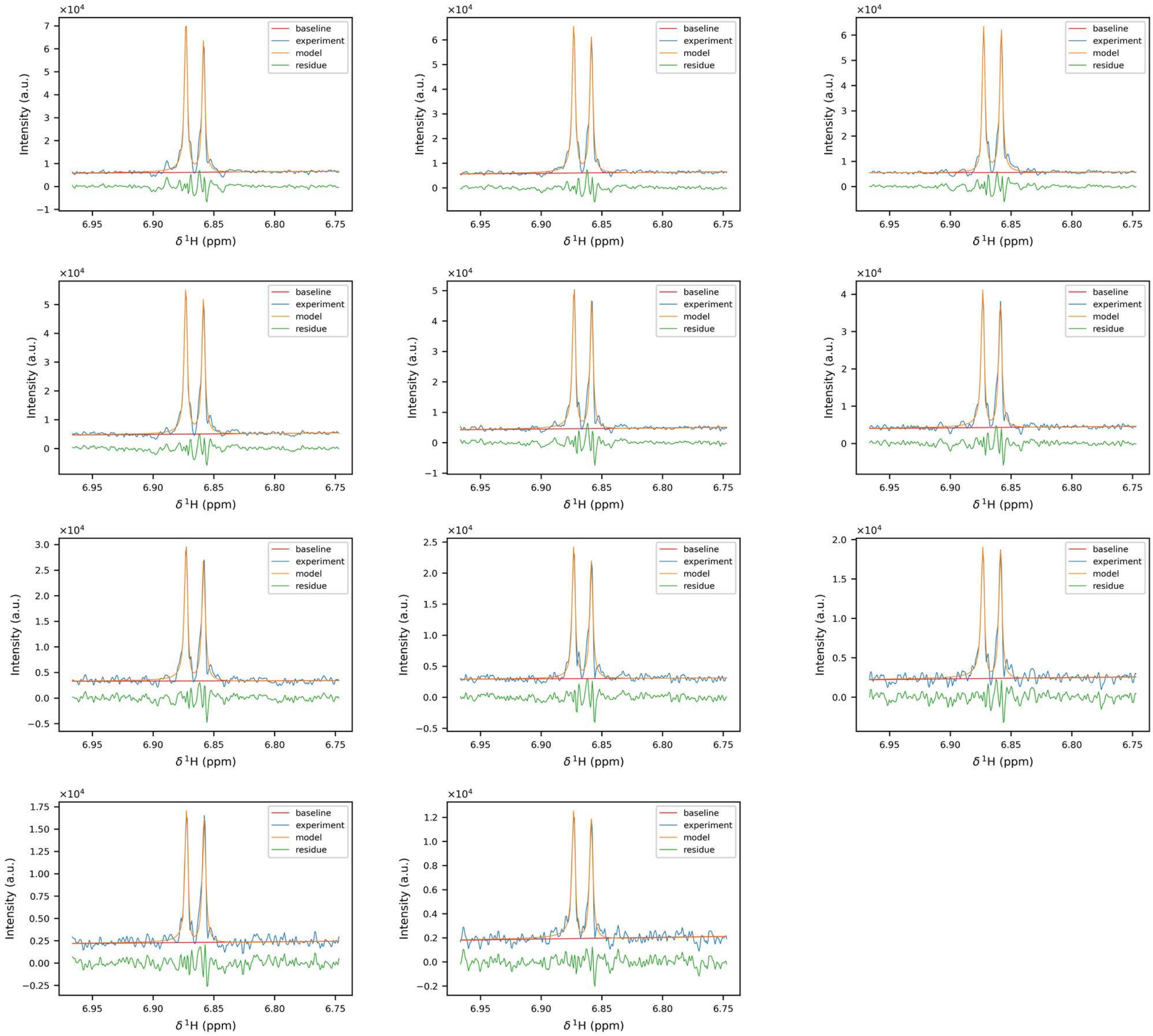
baseline
experiment
model
residue
Intensity (a.u.)
δ ¹H (ppm)

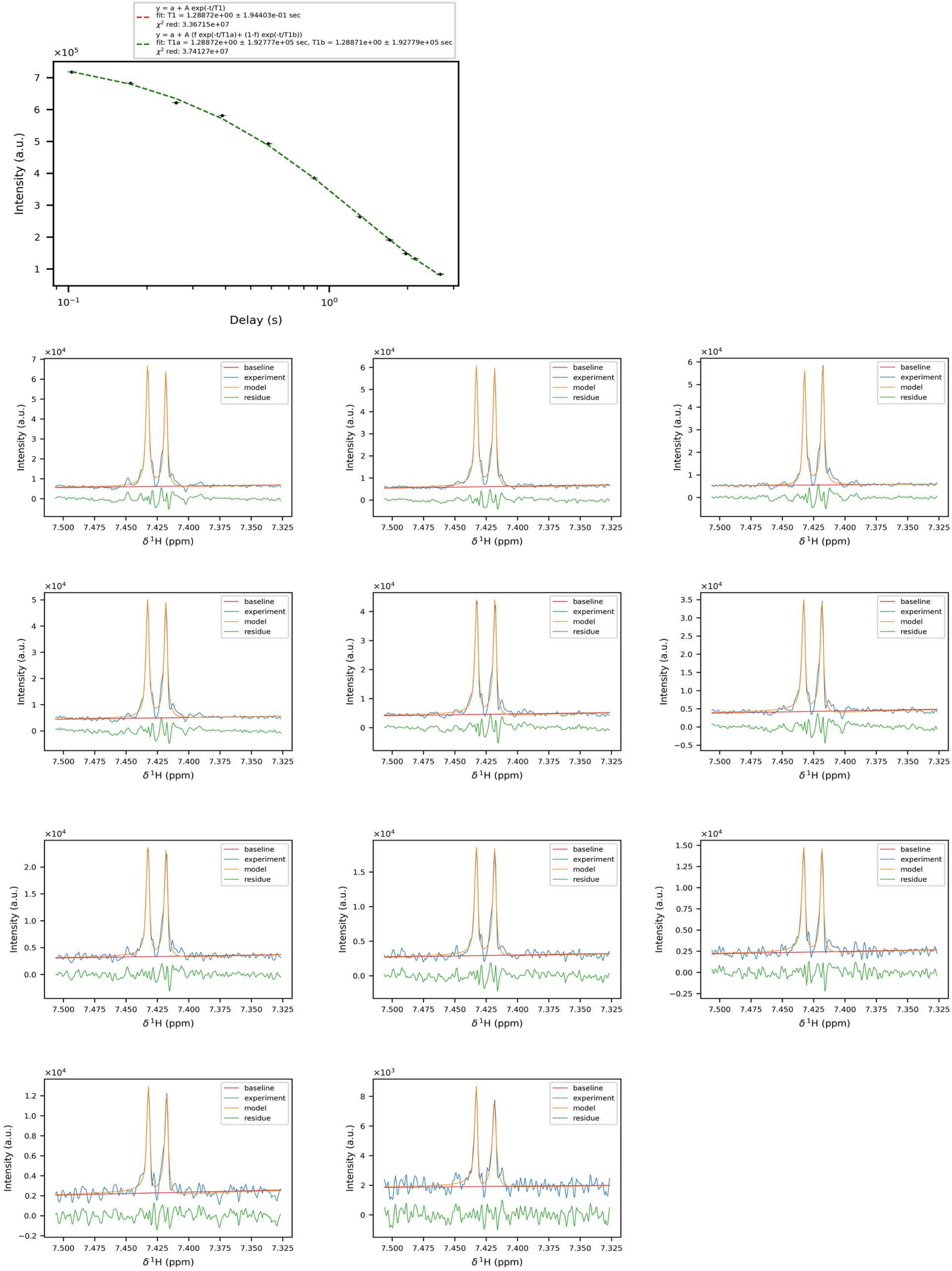

Figure S3: Experimental and fitted NMR doublets of the BPN ligand in the absence of the MMP-12 catalytic domain, acquired at 11 interleaved relaxation delays (0.1, 1.967, 1.311, 0.170, 0.873, 0.256, 0.581, 1.705, 2.131, 0.386, 2.664 s, here displayed from shortest to longest) at 997 mT/42 MHz. The first panels report the derived complete fitted amplitude decays as a function of the relaxation delay.

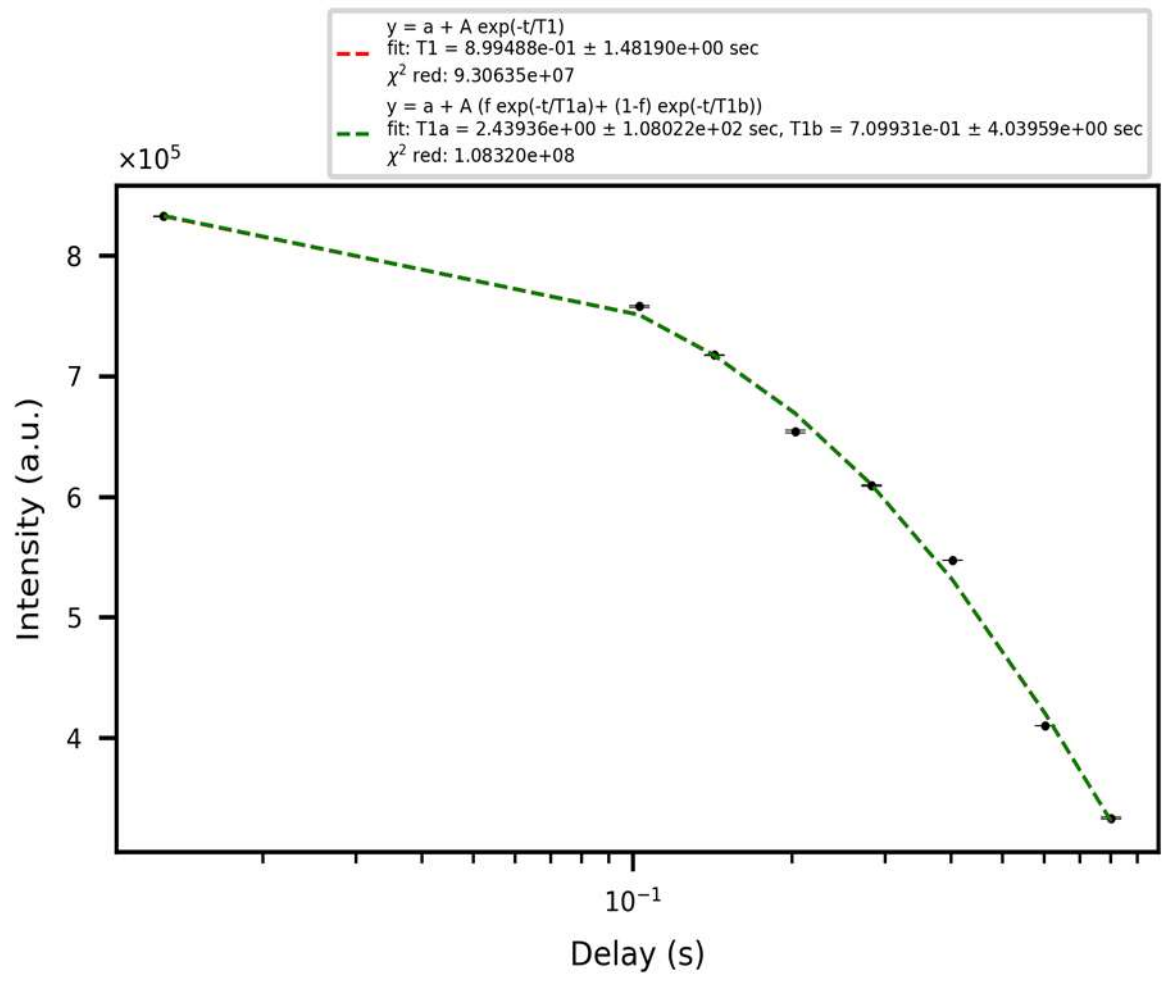
y = a + A exp(-t/T1)
fit: T1 = 8.99488e-01 ± 1.48190e+00 sec
χ2 red: 9.30635e+07
y = a + A (f exp(-t/T1a)+ (1-f) exp(-t/T1b))
fit: T1a = 2.43936e+00 ± 1.08022e+02 sec, T1b = 7.09931e-01 ± 4.03959e+00 sec
χ2 red: 1.08320e+08
Intensity (a.u.)
Delay (s)

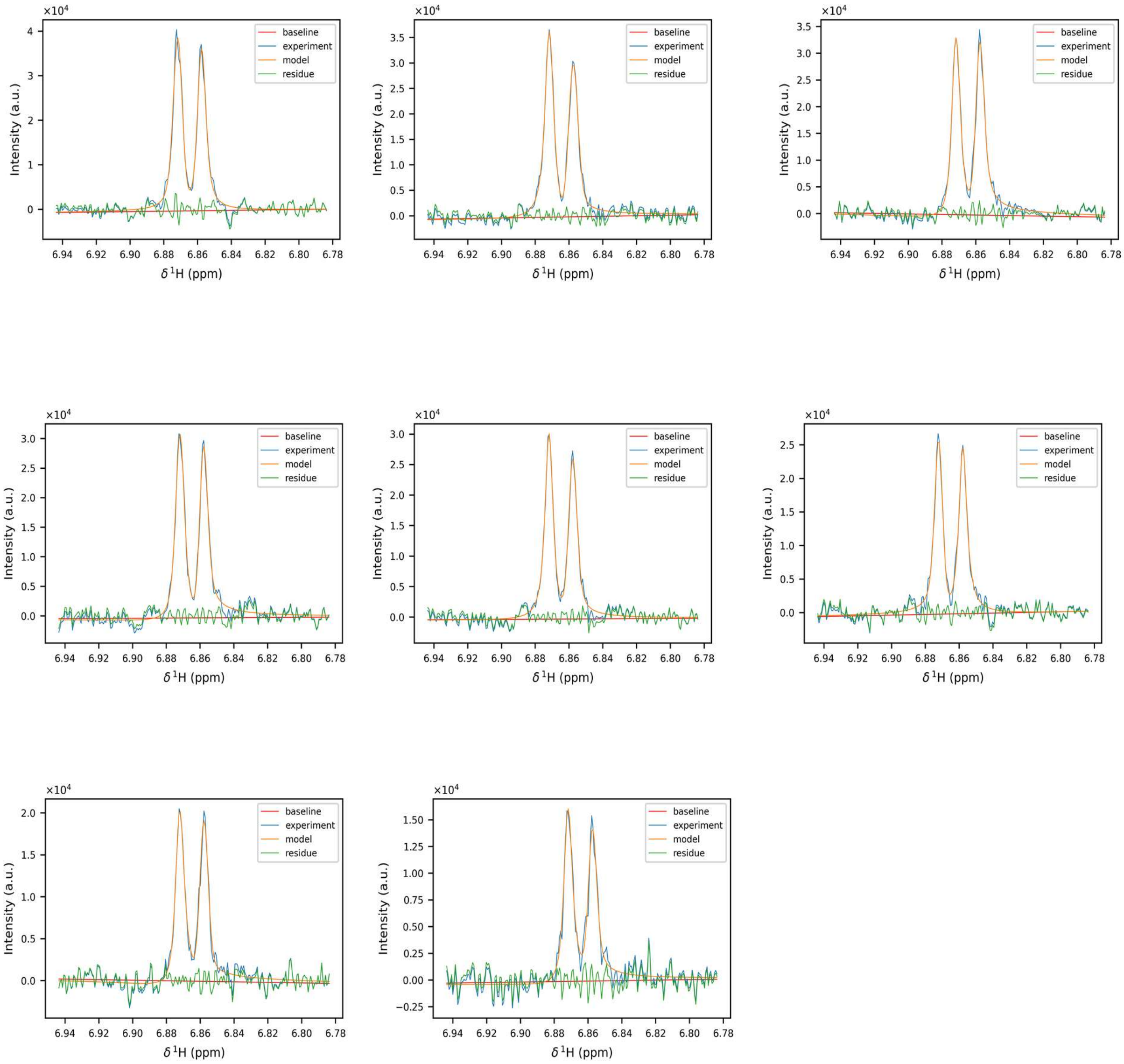
baseline
experiment
model
residue
Intensity (a.u.)
δ 1H (ppm)

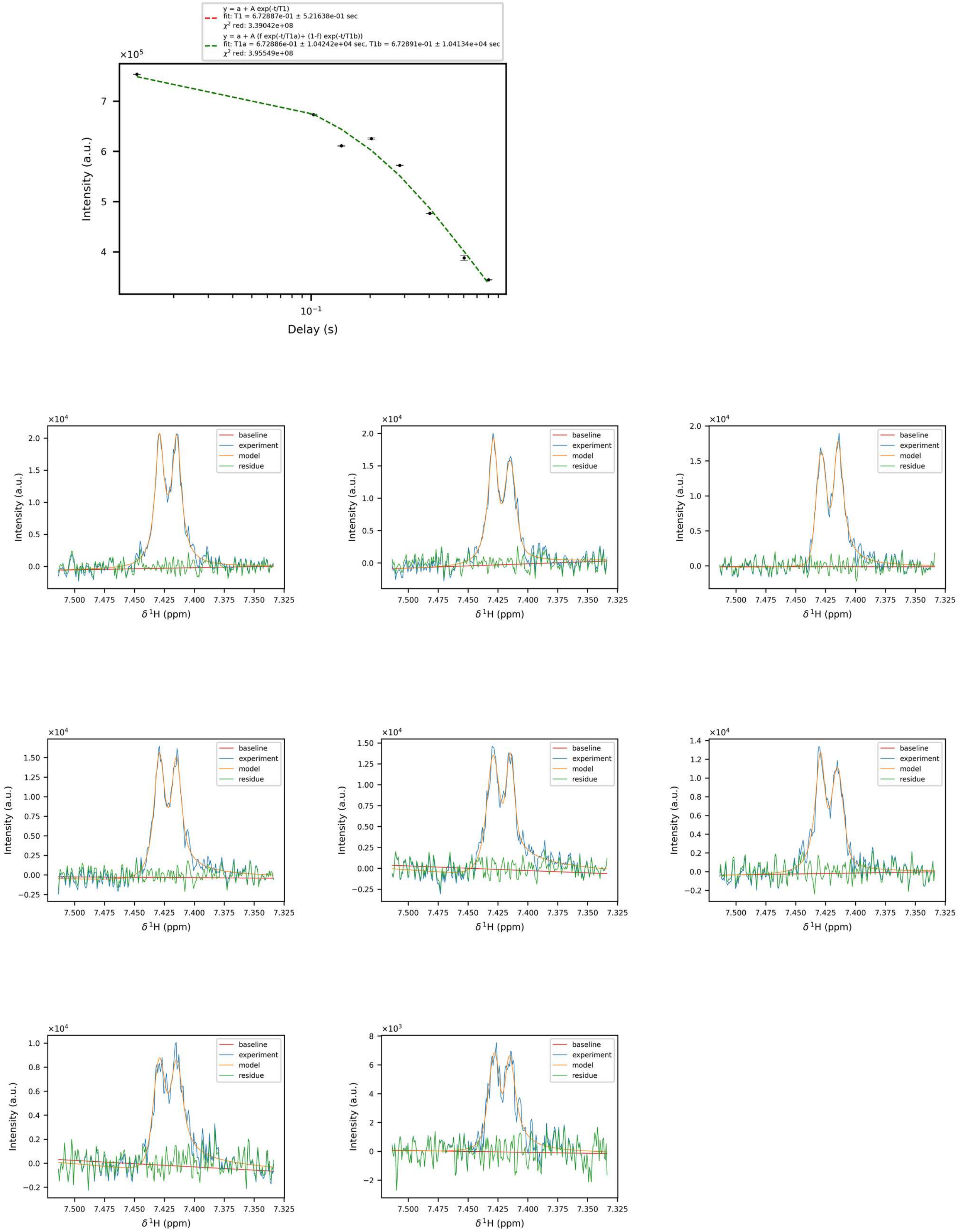

Figure S4: Experimental and fitted NMR doublets of the BPN ligand in the presence of the MMP-12 catalytic domain, acquired at 8 interleaved relaxation delays (0.01, 0.40, 0.28, 0.14, 0.8, 0.20, 0.10, 0.6 s, here displayed from shortest to longest) at 625 mT/26 MHz. The first panels report the derived complete fitted amplitude decays as a function of the relaxation delay.

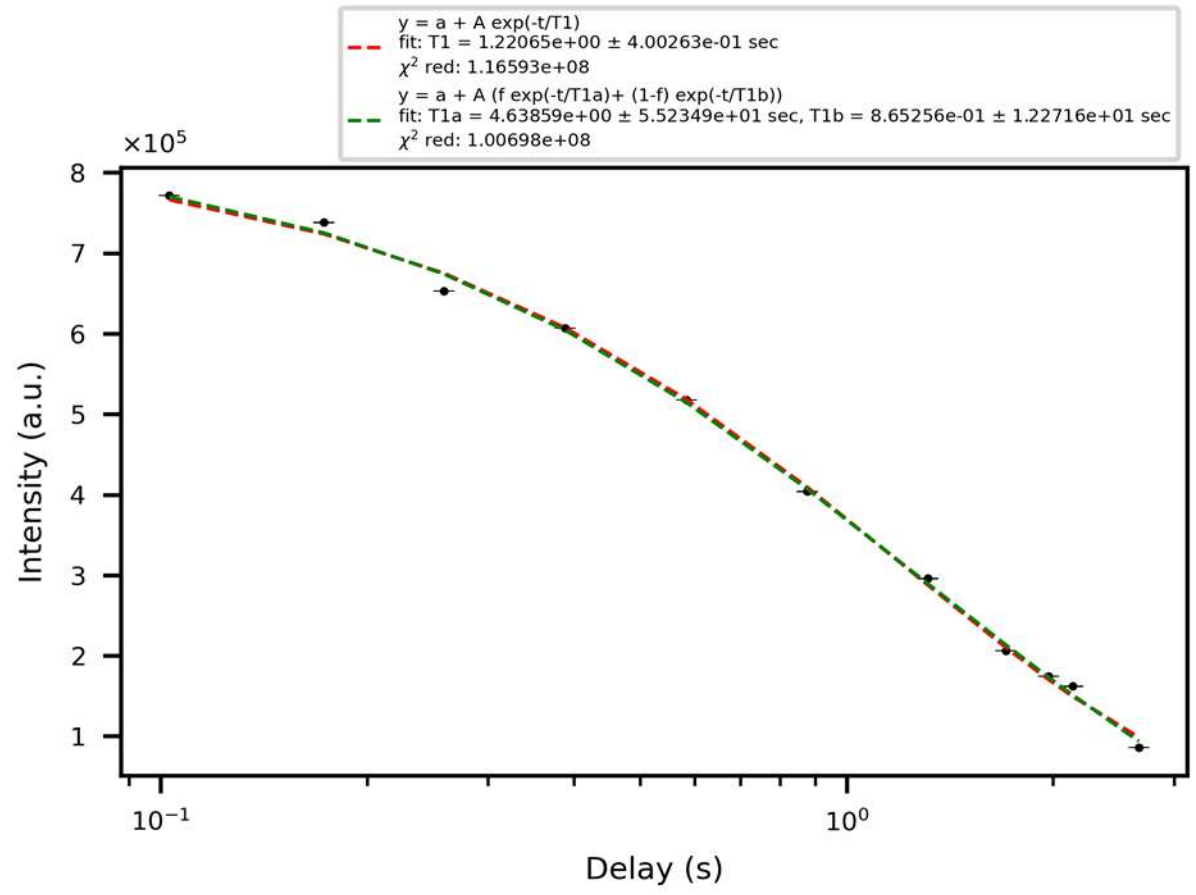
y = a + A exp(-t/T1)
fit: T1 = 1.22065e+00 ± 4.00263e-01 sec
χ² red: 1.16593e+08
y = a + A (f exp(-t/T1a)+ (1-f) exp(-t/T1b))
fit: T1a = 4.63859e+00 ± 5.52349e+01 sec, T1b = 8.65256e-01 ± 1.22716e+01 sec
χ² red: 1.00698e+08
Intensity (a.u.)
Delay (s)

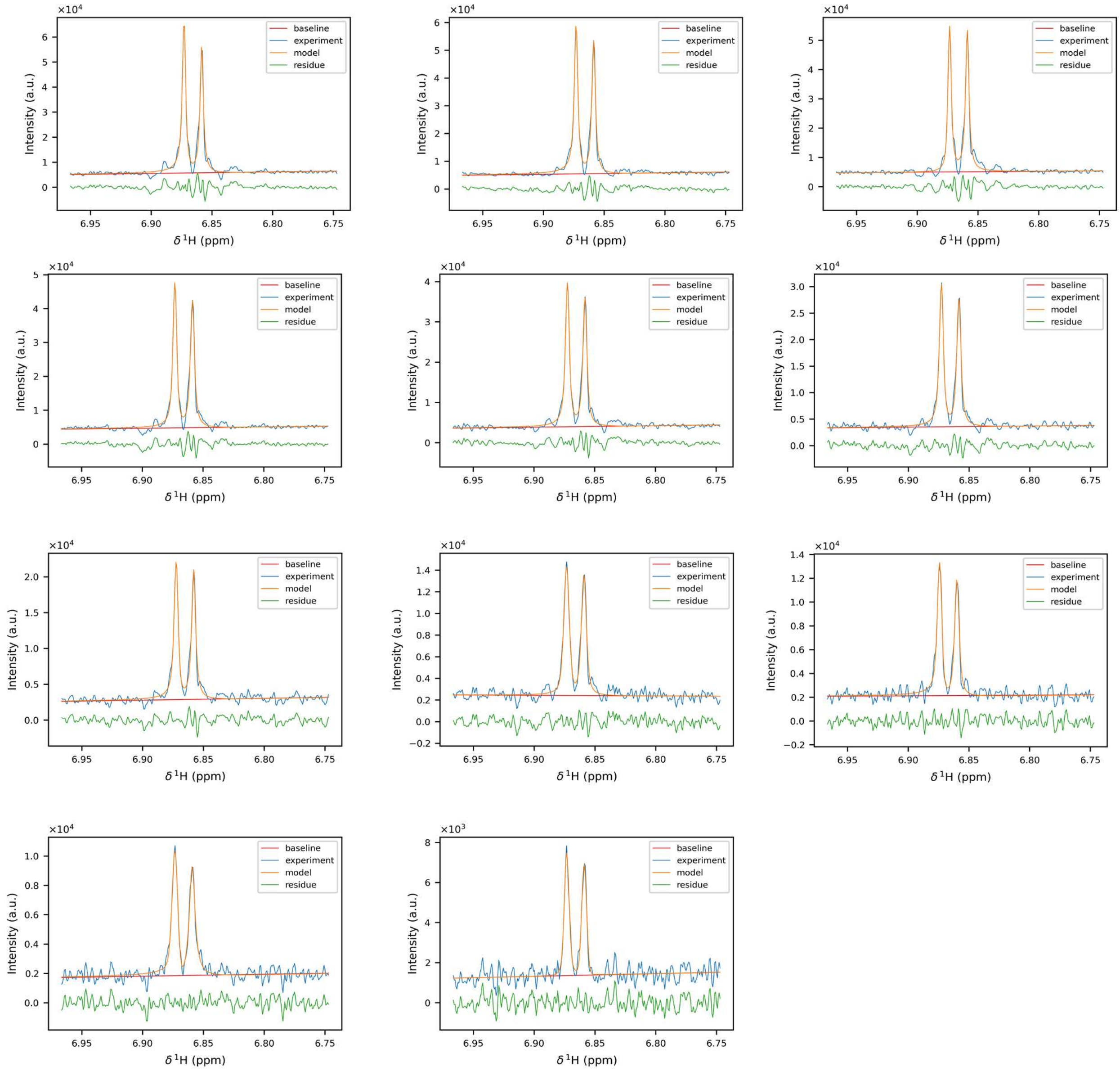
baseline
experiment
model
residue
Intensity (a.u.)
δ ¹H (ppm)

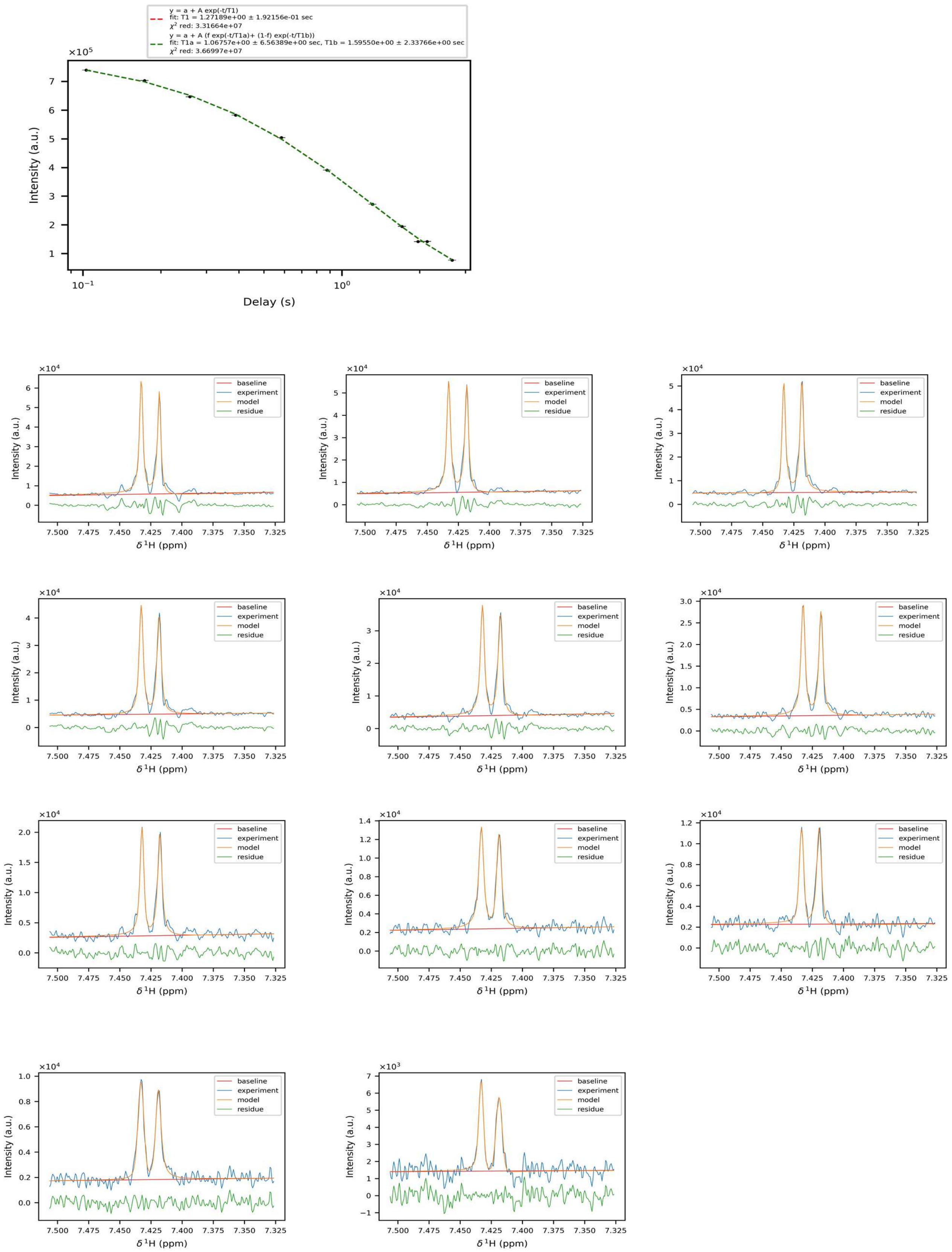

Figure S5: Experimental and fitted NMR doublets of the BPN ligand in the absence of the MMP-12 catalytic domain, acquired at 11 interleaved relaxation delays (0.1, 1.967, 1.311, 0.170, 0.873, 0.256, 0.581, 1.705, 2.131, 0.386, 2.664 s, here displayed from shortest to longest) at 251 mT/10 MHz. The first panels report the derived complete fitted amplitude decays as a function of the relaxation delay.

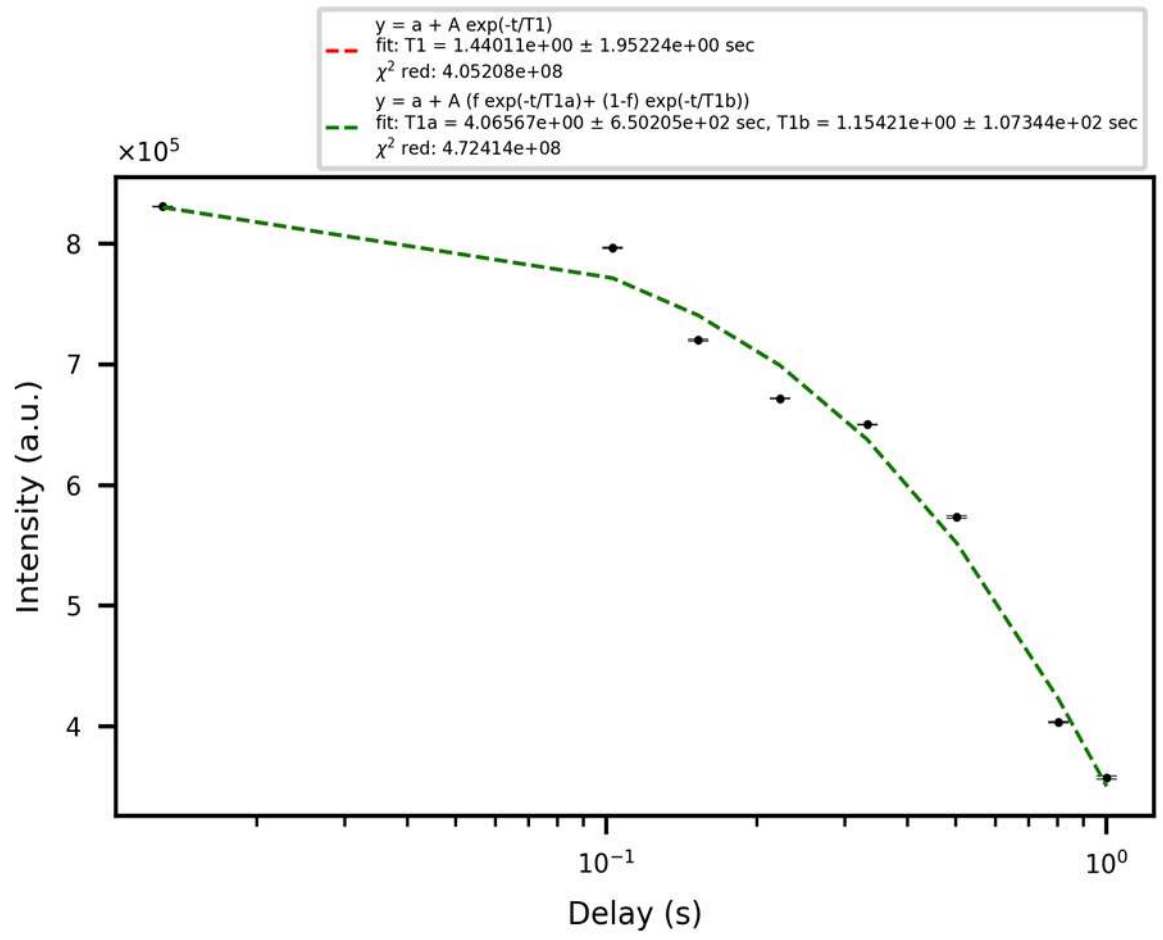
y = a + A exp(-t/T1)
fit: T1 = 1.44011e+00 ± 1.95224e+00 sec
χ2 red: 4.05208e+08
y = a + A (f exp(-t/T1a)+ (1-f) exp(-t/T1b))
fit: T1a = 4.06567e+00 ± 6.50205e+02 sec, T1b = 1.15421e+00 ± 1.07344e+02 sec
χ2 red: 4.72414e+08
×10^5
Intensity (a.u.)
Delay (s)

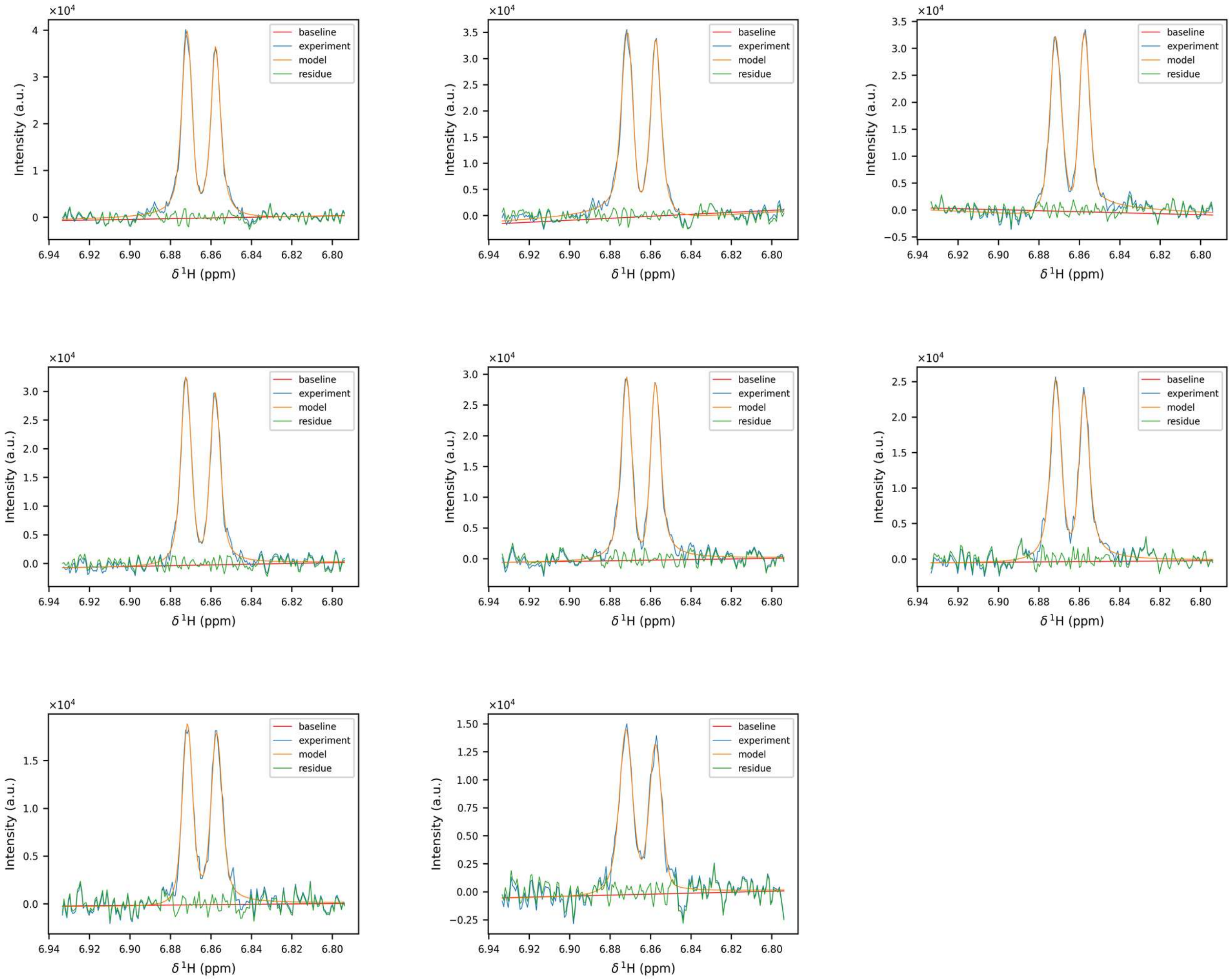
baseline
experiment
model
residue
Intensity (a.u.)
δ 1H (ppm)

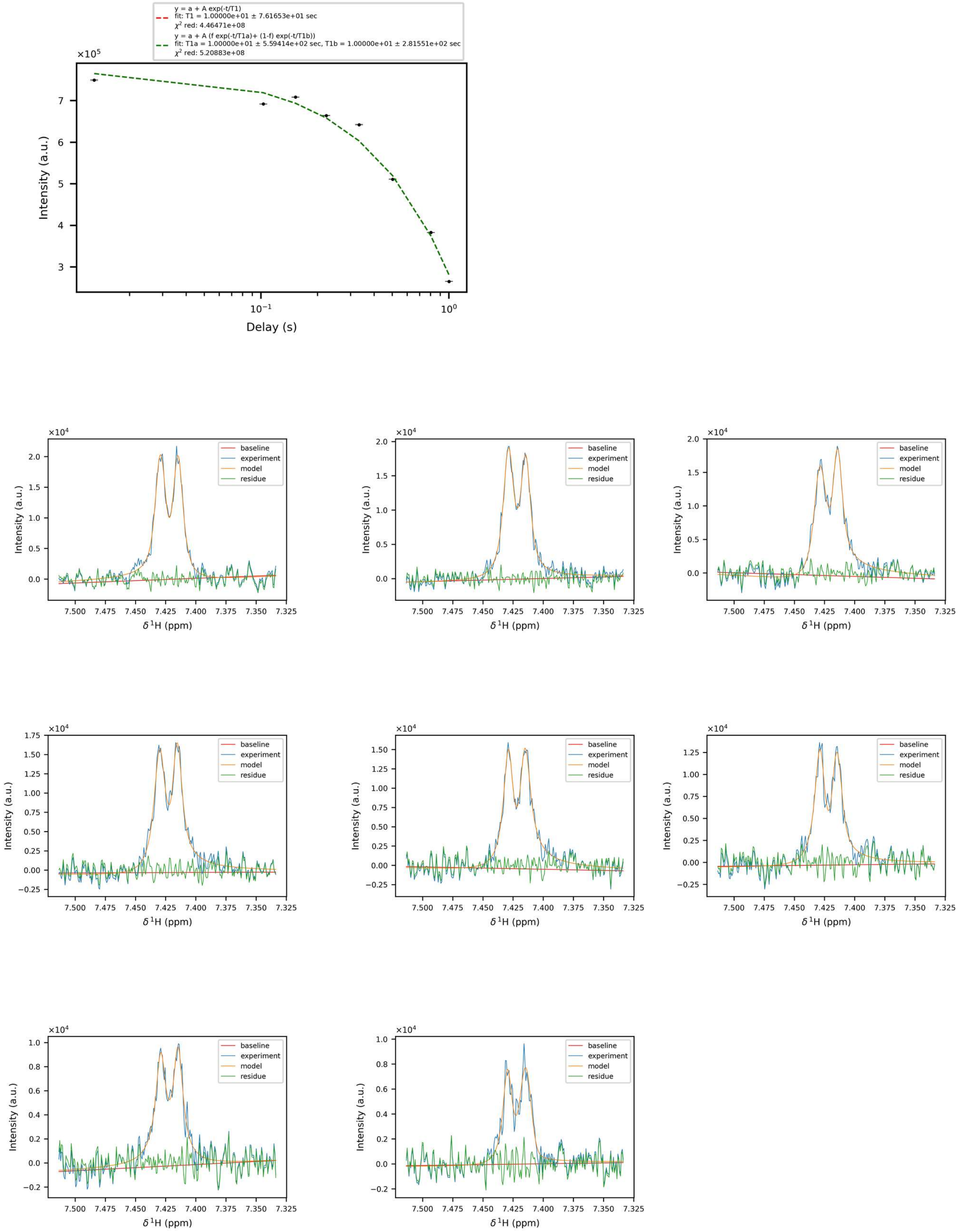

Figure S6: Experimental and fitted NMR doublets of the BPN ligand in the presence of the MMP-12 catalytic domain, acquired at 8 interleaved relaxation delays (0.01, 0.50, 0.22, 0.33, 0.8, 0.10, 1, 0.15 s, here displayed from shortest to longest) at 1496 mT/63 MHz. The first panels report the derived complete fitted amplitude decays as a function of the relaxation delay.

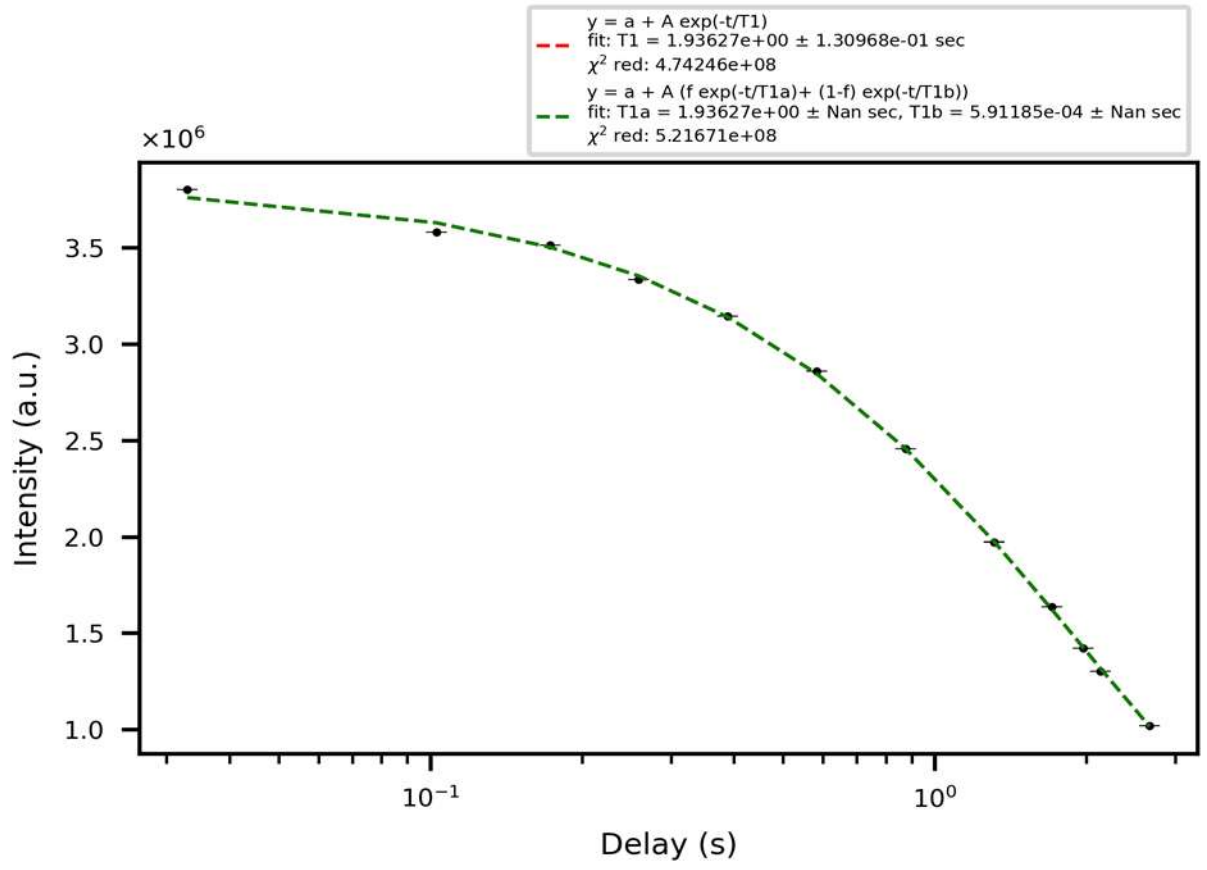
y = a + A exp(-t/T1)
fit: T1 = 1.93627e+00 ± 1.30968e-01 sec
χ² red: 4.74246e+08
y = a + A (f exp(-t/T1a)+ (1-f) exp(-t/T1b))
fit: T1a = 1.93627e+00 ± Nan sec, T1b = 5.91185e-04 ± Nan sec
χ² red: 5.21671e+08
Intensity (a.u.)
Delay (s)

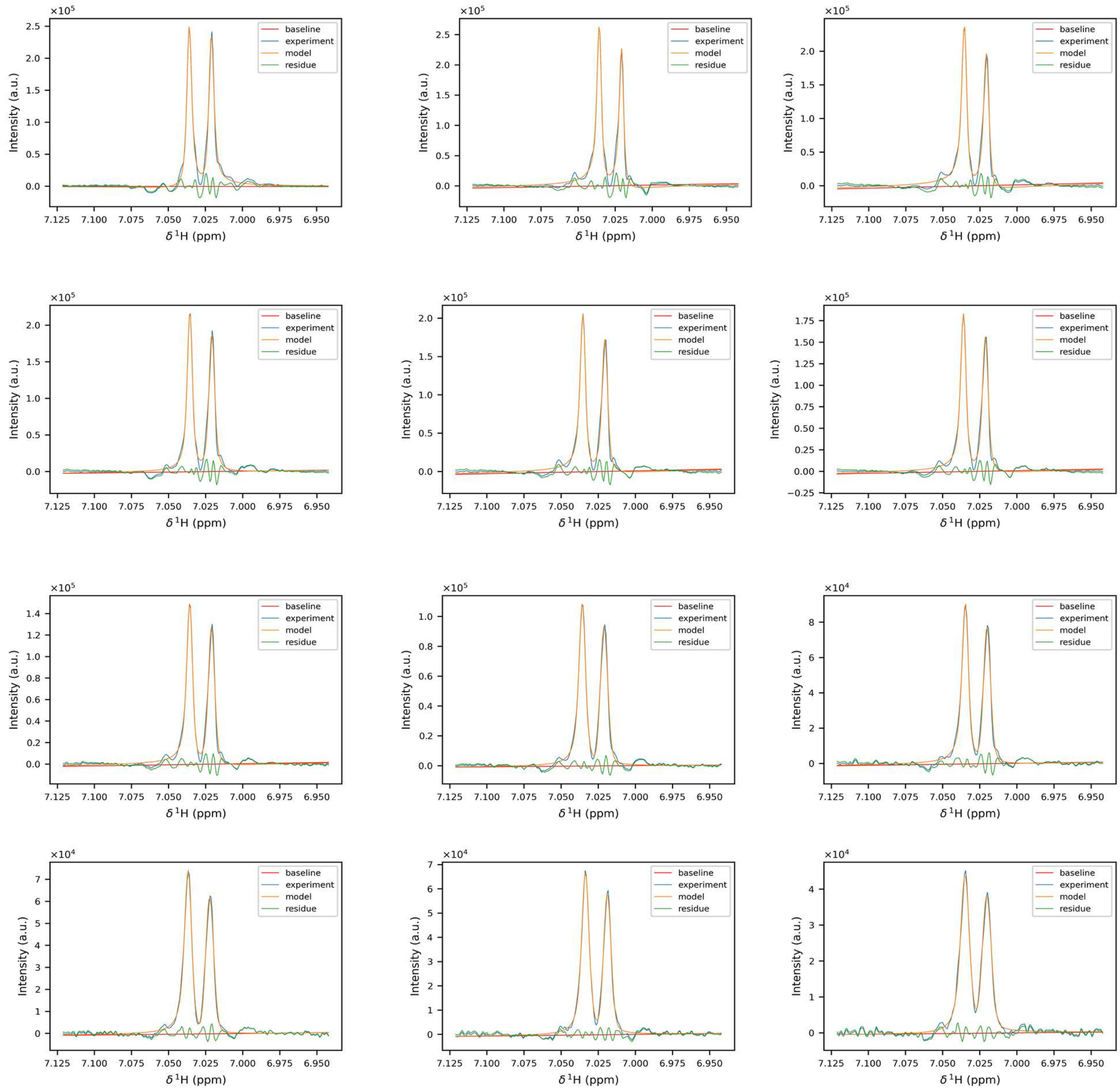
baseline
experiment
model
residue
Intensity (a.u.)
δ ¹H (ppm)

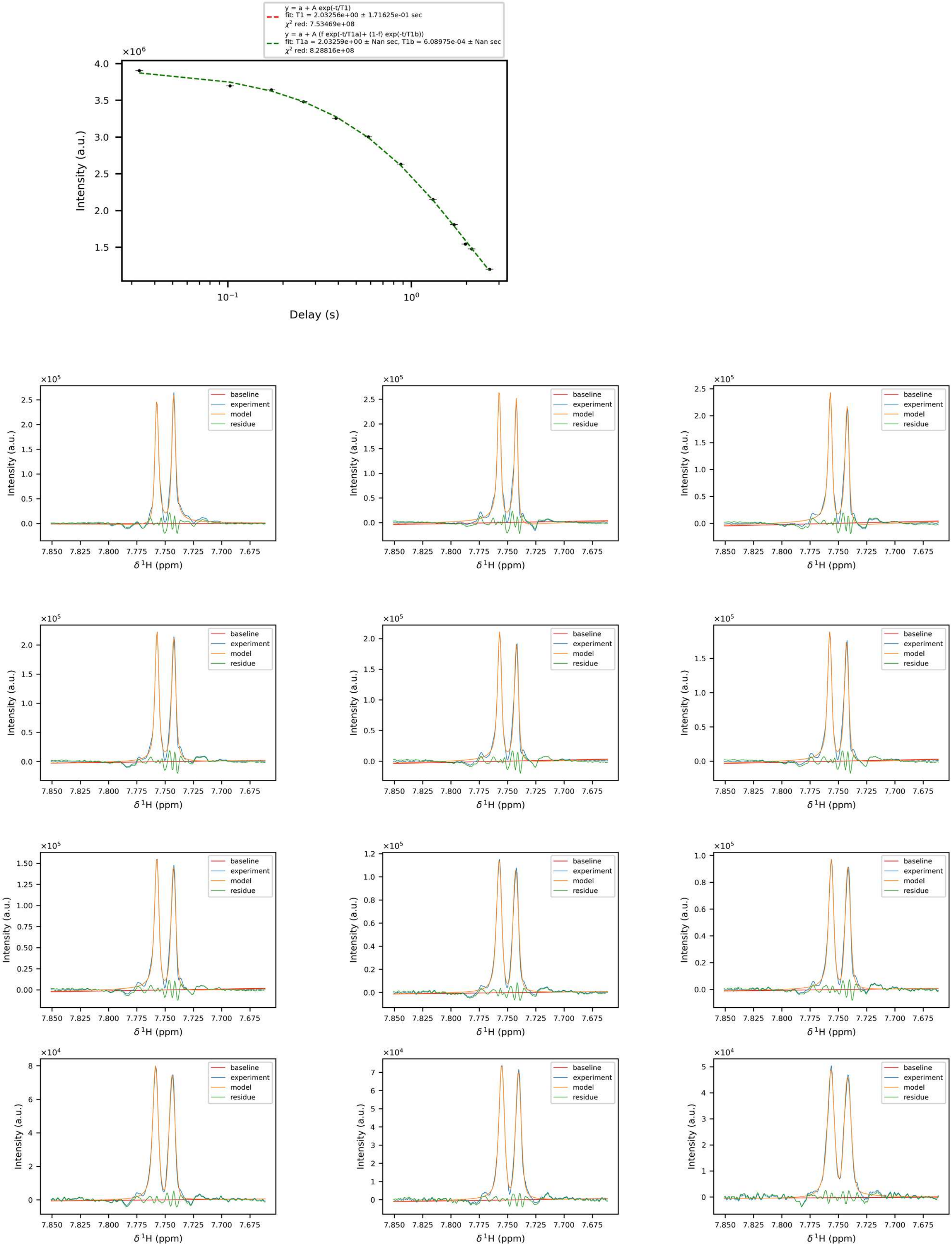

Figure S7: Experimental and fitted NMR doublets of the MBS ligand in the absence of the MMP-12 catalytic domain, acquired at 12 interleaved relaxation delays (0.1, 1.967, 1.311, 0.170, 0.873, 0.256, 0.581, 1.705, 2.131, 0.386, 2.664, 0.03 s, here displayed from shortest to longest) at 250 mT/10 MHz. The first panels report the derived complete fitted amplitude decays as a function of the relaxation delay.

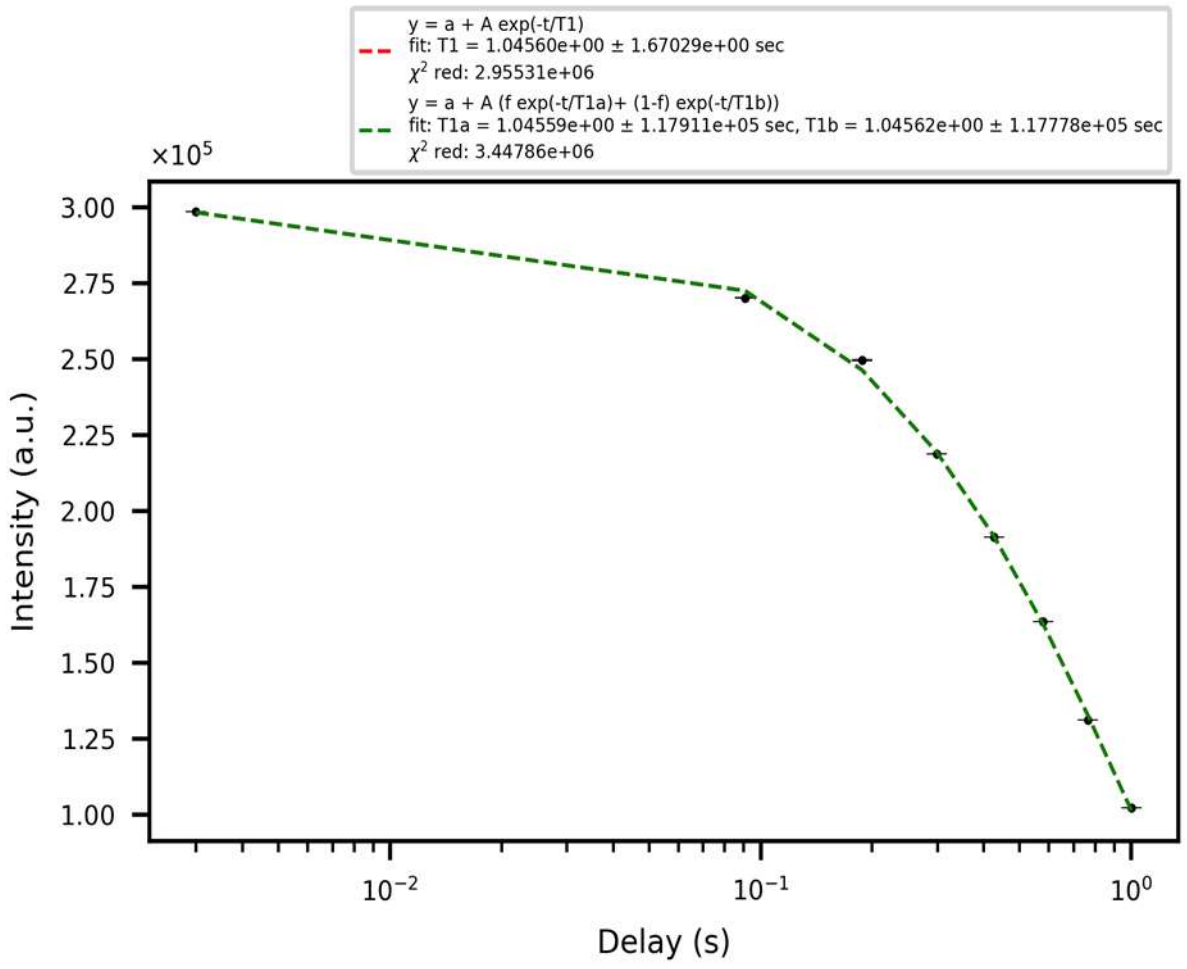
y = a + A exp(-t/T1)
fit: T1 = 1.04560e+00 ± 1.67029e+00 sec
χ² red: 2.95531e+06
y = a + A (f exp(-t/T1a)+ (1-f) exp(-t/T1b))
fit: T1a = 1.04559e+00 ± 1.17911e+05 sec, T1b = 1.04562e+00 ± 1.17778e+05 sec
χ² red: 3.44786e+06
×10⁵
Intensity (a.u.)
Delay (s)

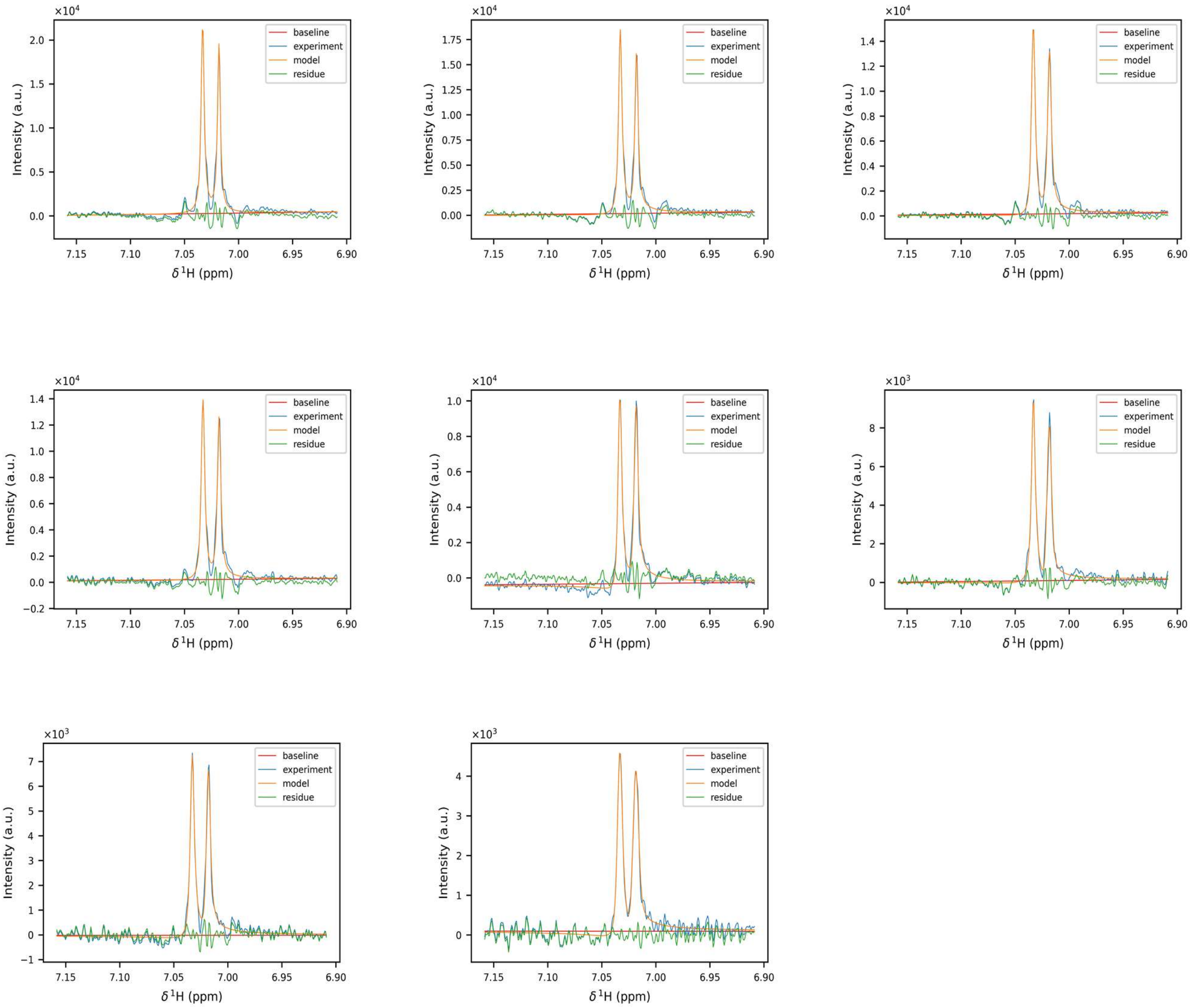
baseline
experiment
model
residue
Intensity (a.u.)
δ ¹H (ppm)

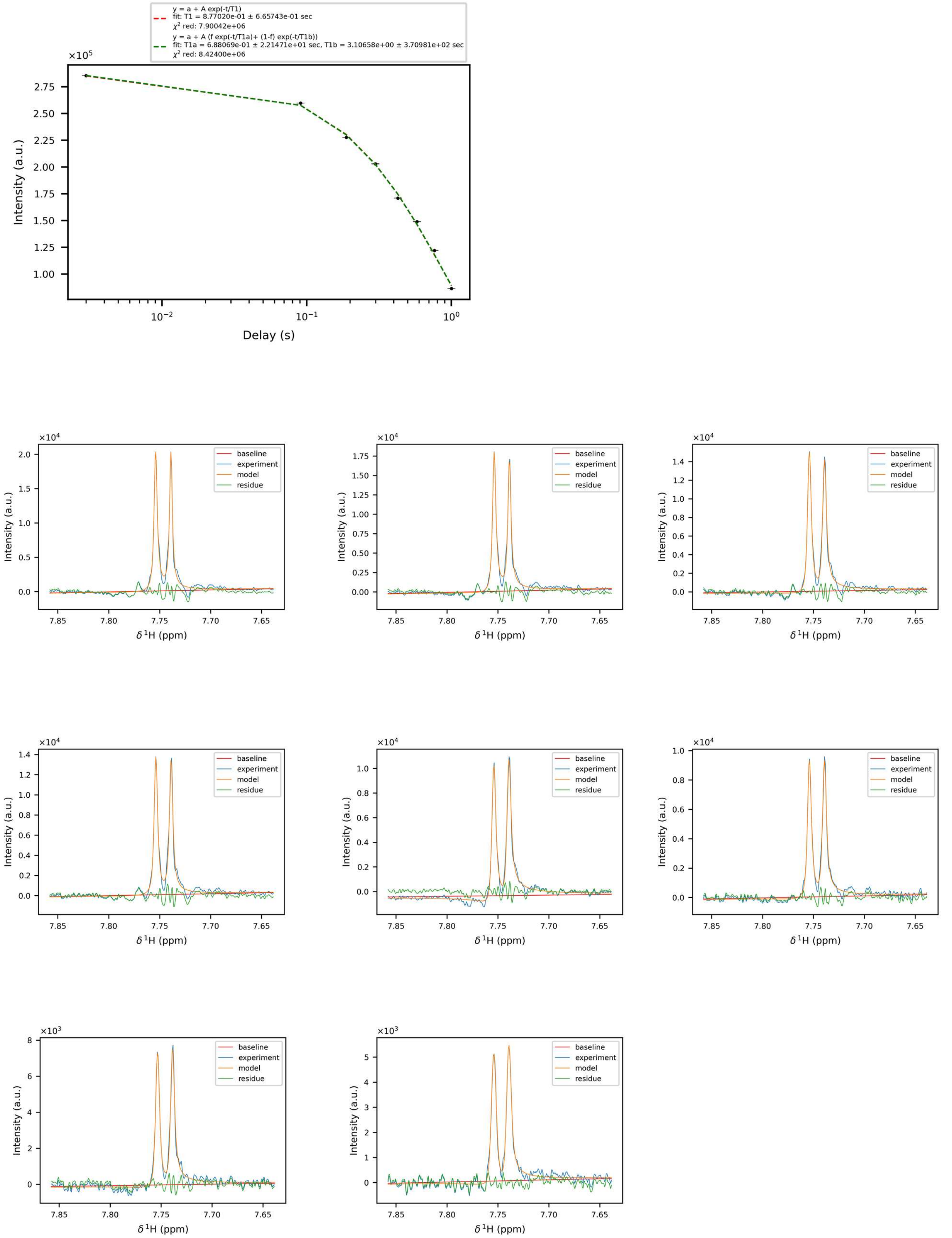

Figure S8: Experimental and fitted NMR doublets of the MBS ligand in the presence of the MMP-12 catalytic domain, acquired at 8 interleaved relaxation delays (0.088, 0.000, 0.185, 1.000 , 0.296, 0.576, 0.424, 0.761 s, here displayed from shortest to longest) at 250 mT/10 MHz. The first panels report the derived complete fitted amplitude decays as a function of the relaxation delay.

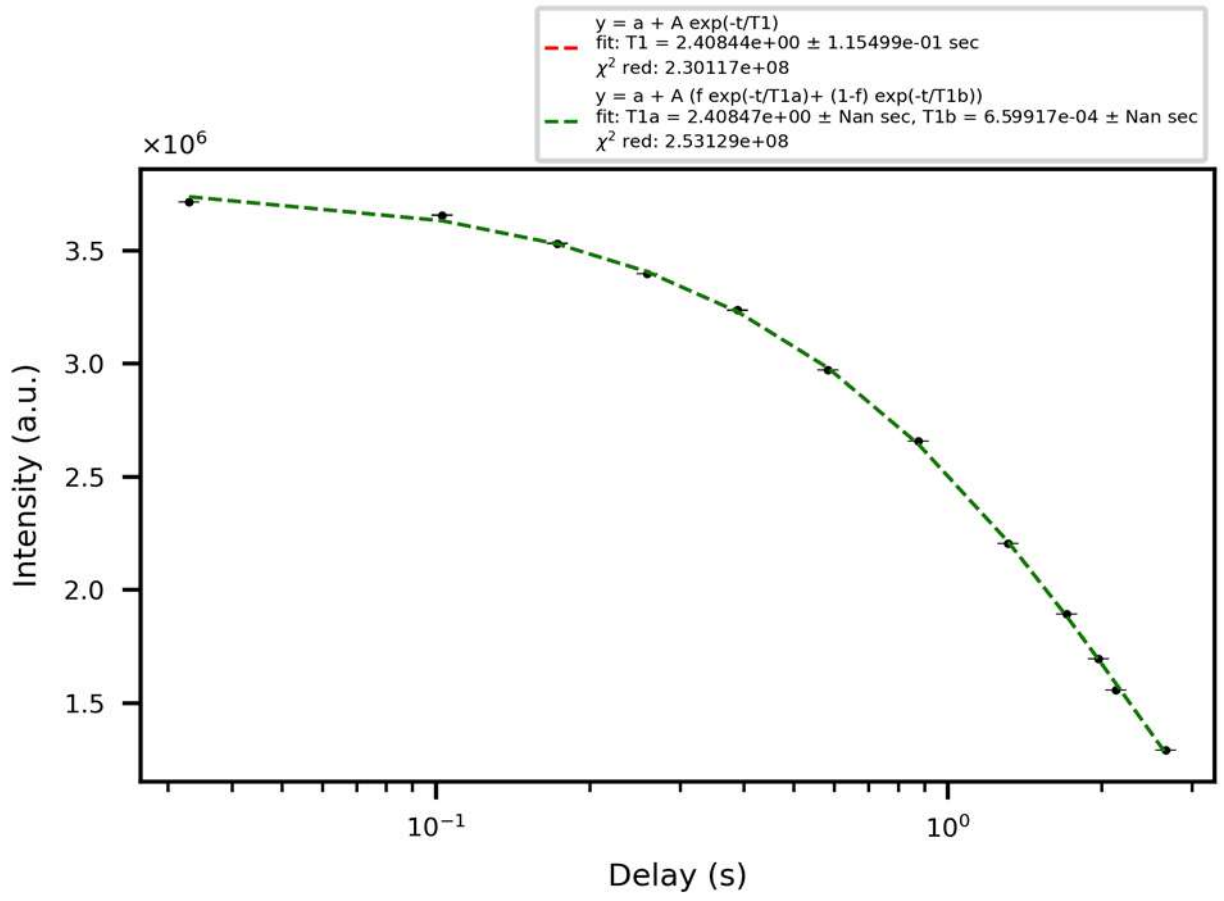
y = a + A exp(-t/T1)
fit: T1 = 2.40844e+00 ± 1.15499e-01 sec
χ² red: 2.30117e+08
y = a + A (f exp(-t/T1a)+ (1-f) exp(-t/T1b))
fit: T1a = 2.40847e+00 ± Nan sec, T1b = 6.59917e-04 ± Nan sec
χ² red: 2.53129e+08
×10⁶
Intensity (a.u.)
Delay (s)

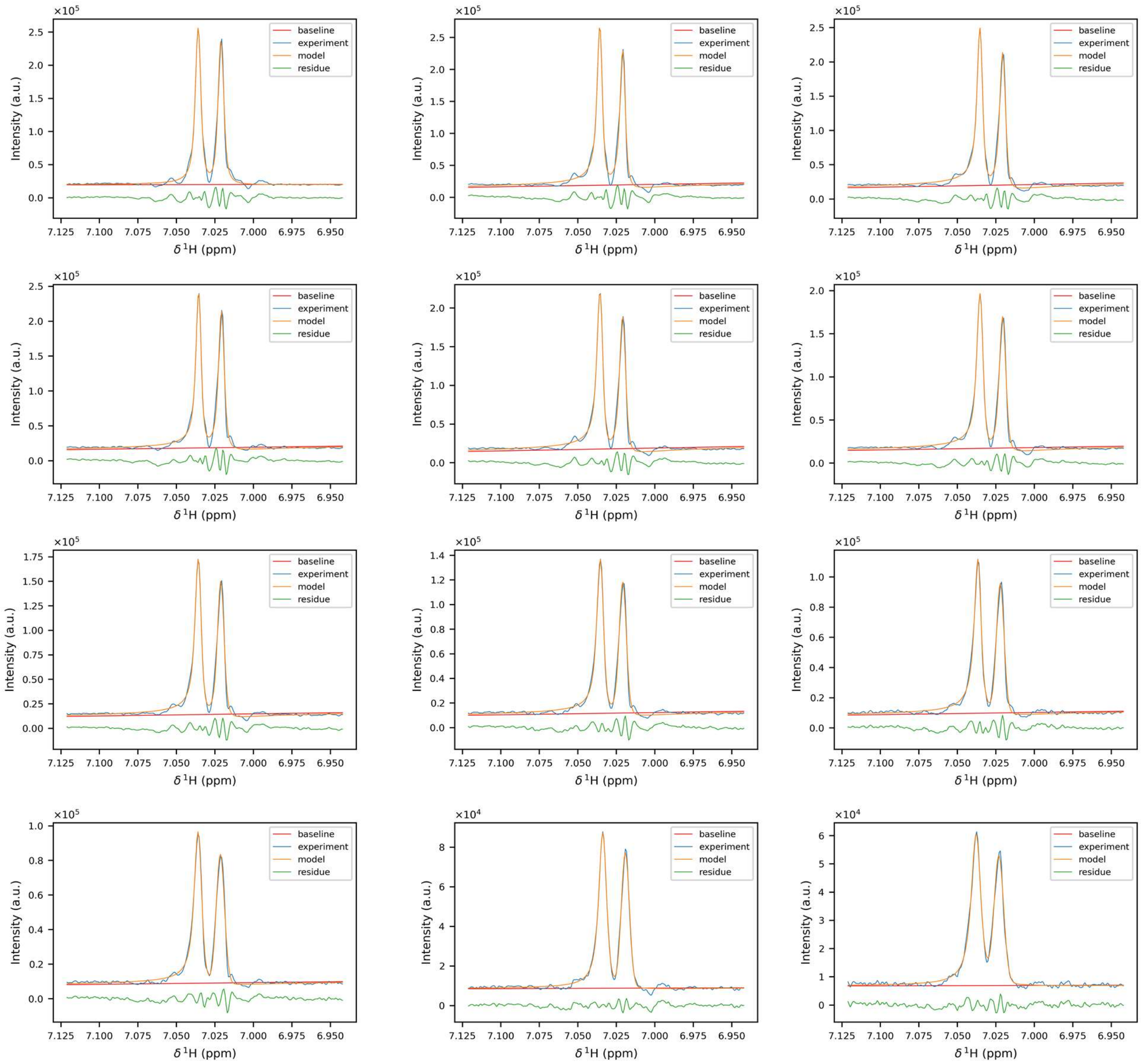
baseline
experiment
model
residue
Intensity (a.u.)
δ ¹H (ppm)

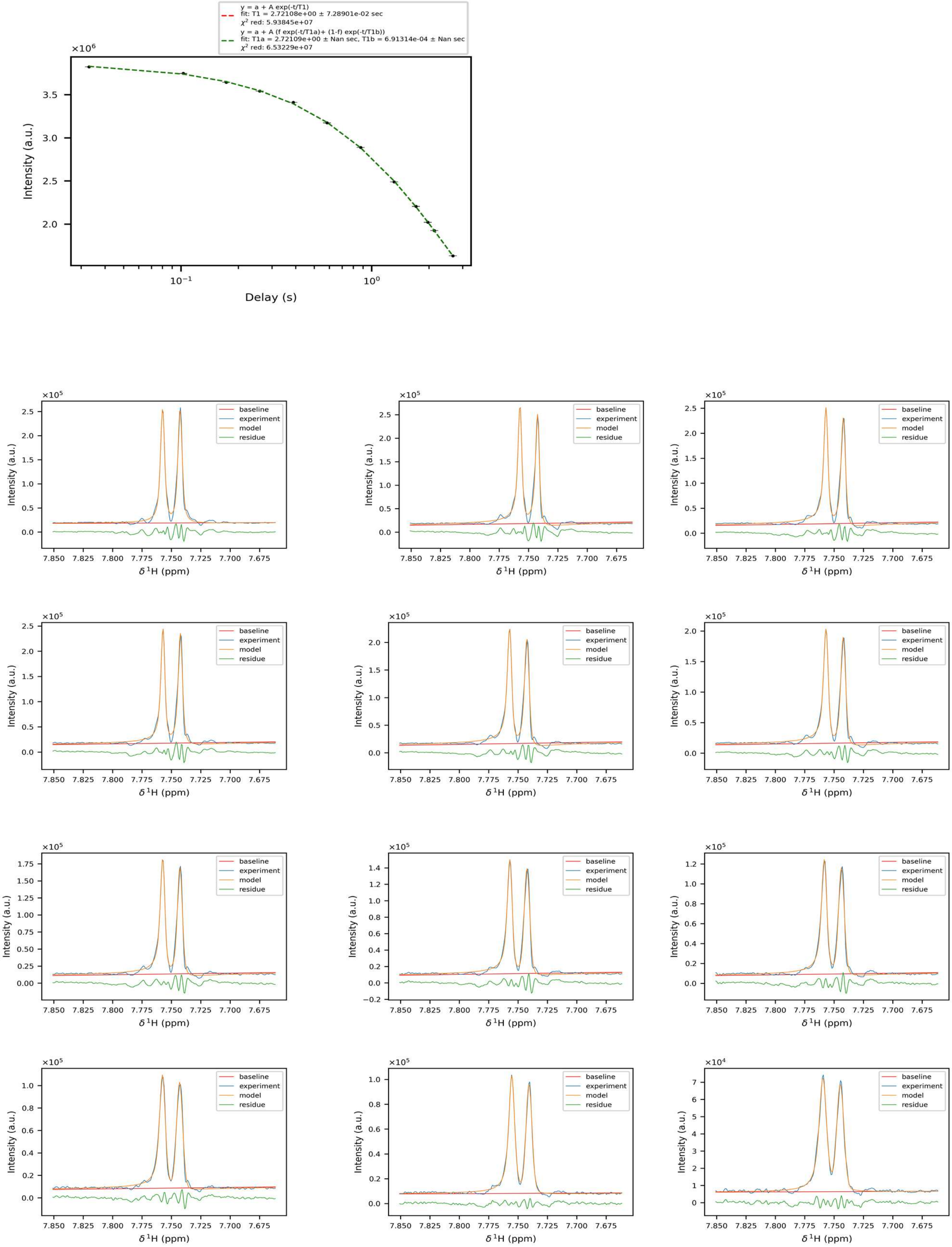

Figure S9: Experimental and fitted NMR doublets of the MBS ligand in the absence of the MMP-12 catalytic domain, acquired at 12 interleaved relaxation delays (0.1, 1.967, 1.311, 0.170, 0.873, 0.256, 0.581, 1.705, 2.131, 0.386, 2.664, 0.03 s, here displayed from shortest to longest) at 4000 mT/168 MHz. The first panels report the derived complete fitted amplitude decays as a function of the relaxation delay.

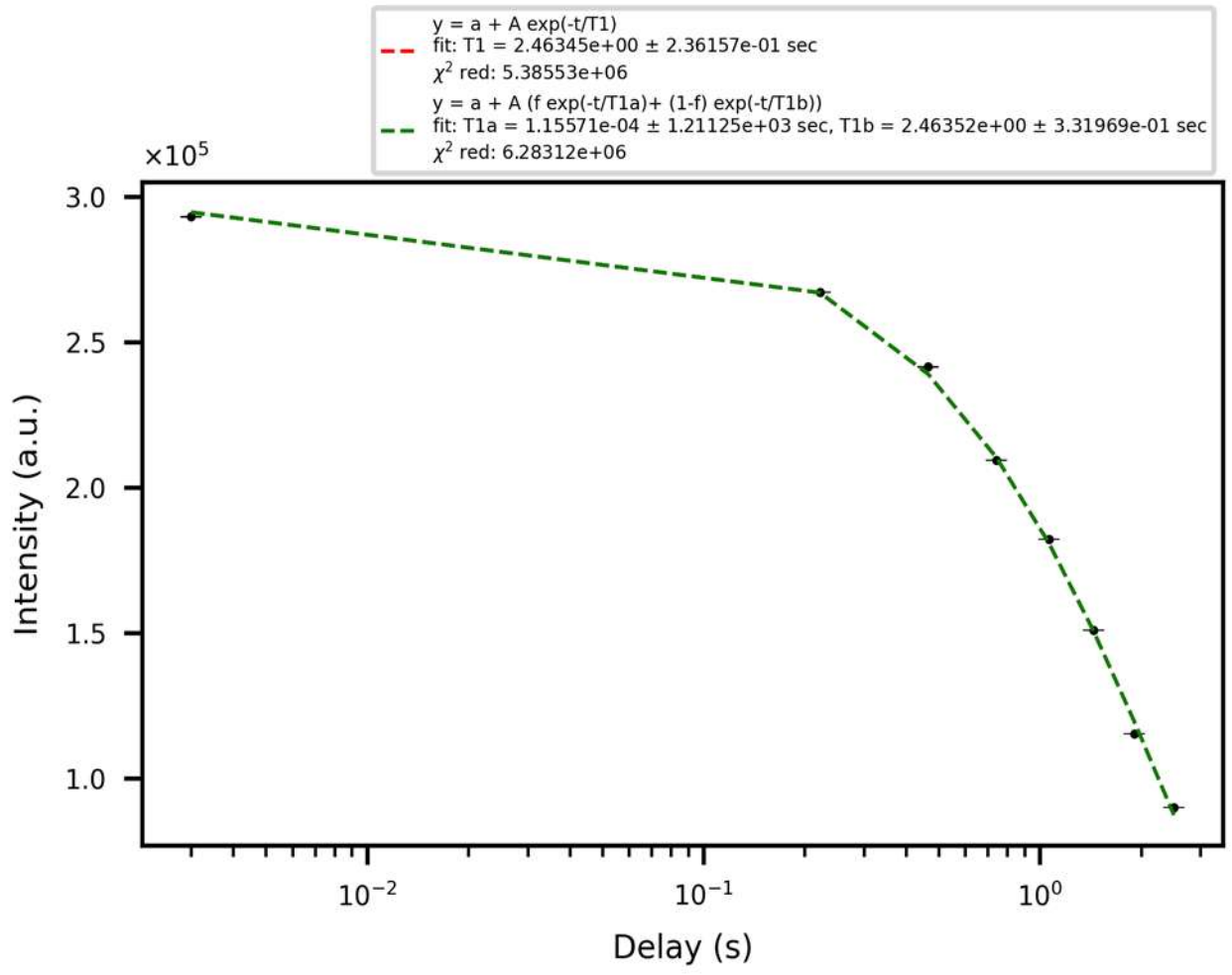
y = a + A exp(-t/T1)
fit: T1 = 2.46345e+00 ± 2.36157e-01 sec
χ² red: 5.38553e+06
y = a + A (f exp(-t/T1a)+ (1-f) exp(-t/T1b))
fit: T1a = 1.15571e-04 ± 1.21125e+03 sec, T1b = 2.46352e+00 ± 3.31969e-01 sec
χ² red: 6.28312e+06
×10⁵
Intensity (a.u.)
Delay (s)

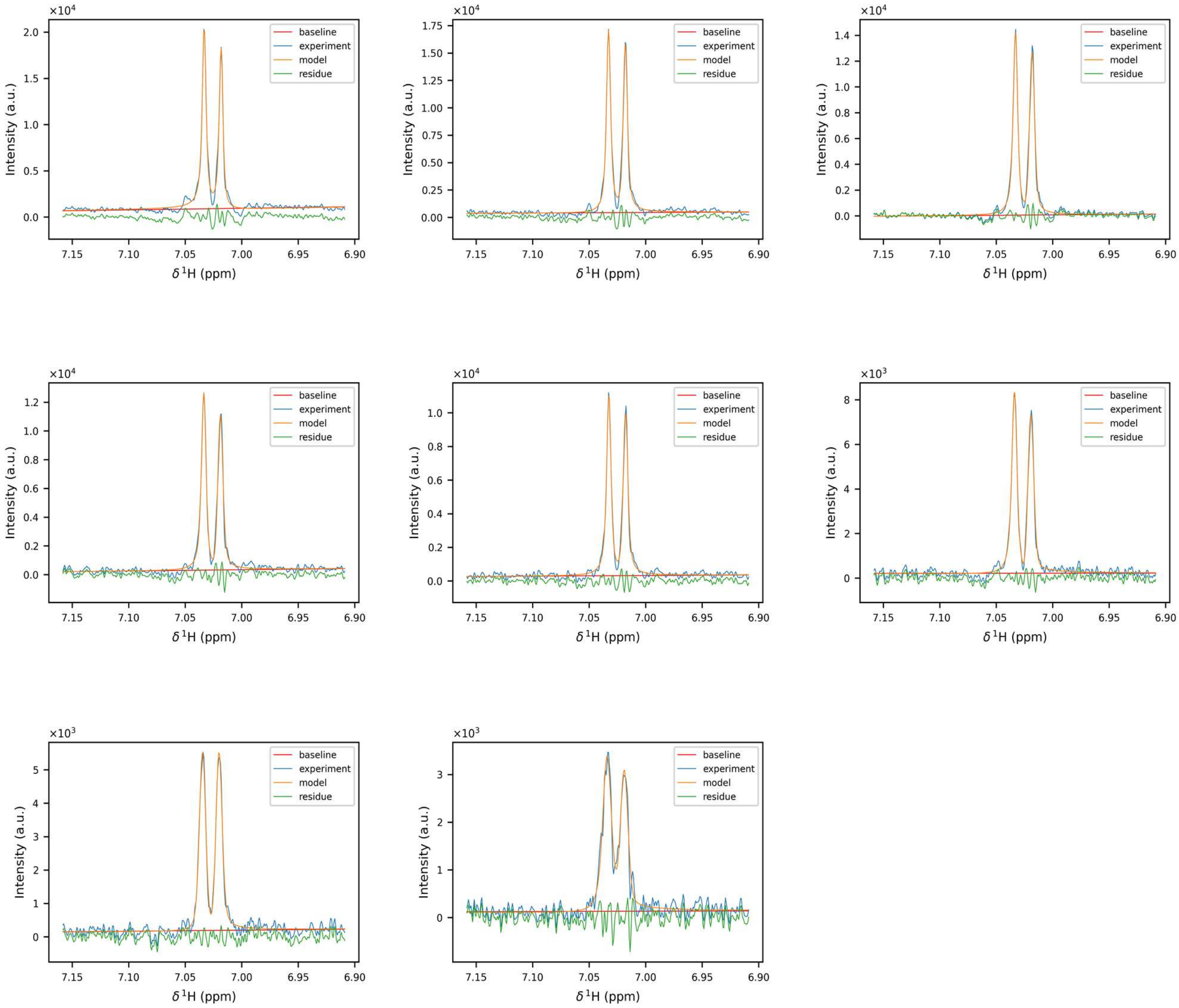
baseline
experiment
model
residue
Intensity (a.u.)
δ ¹H (ppm)

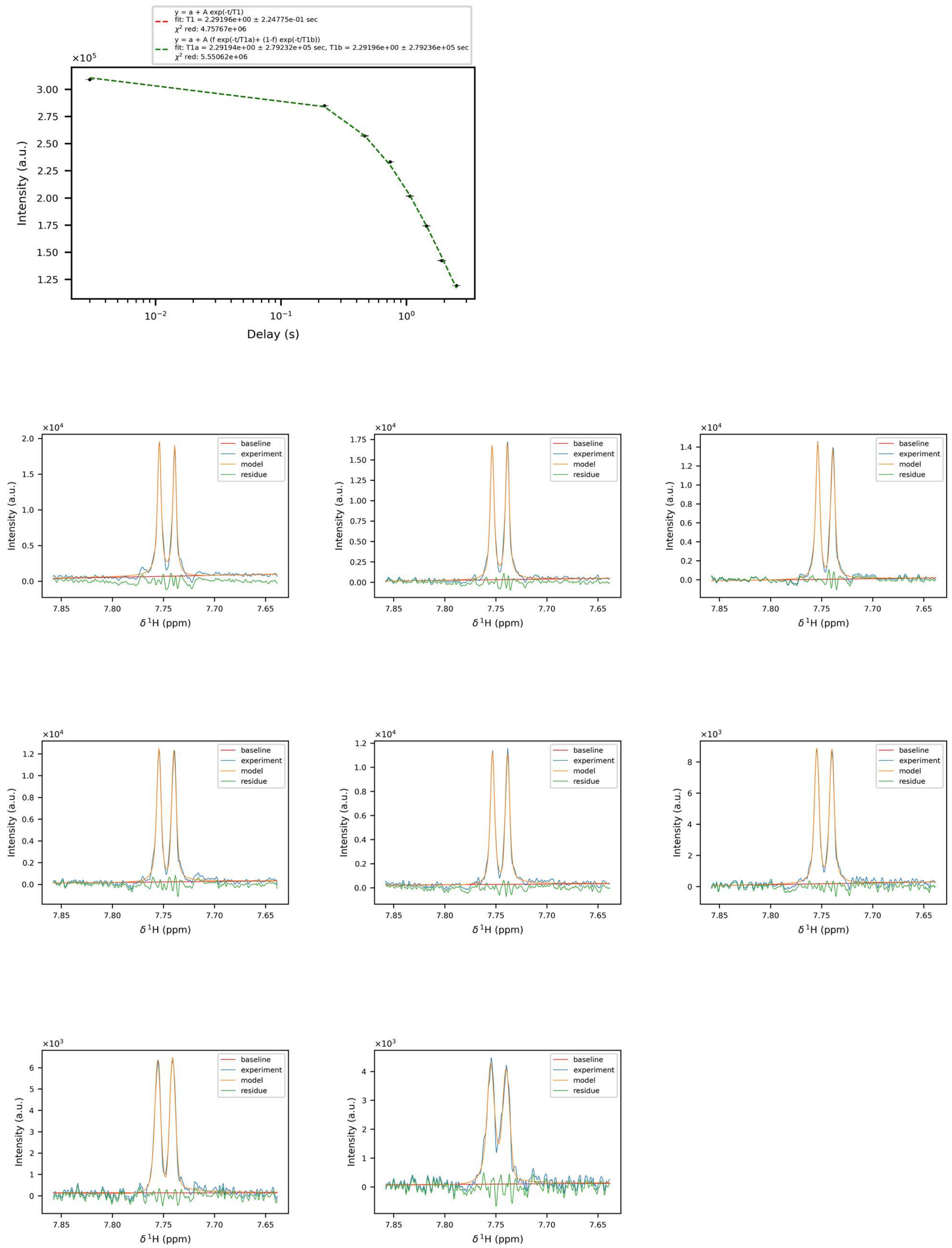

Figure S10: Experimental and fitted NMR doublets of the MBS ligand in the presence of the MMP-12 catalytic domain, acquired at 8 interleaved relaxation delays (1.903, 0.741, 0.219, 1.061, 1.439, 0.000, 0.463, 2.500 s, here displayed from shortest to longest) at 4000 mT/168 MHz. The first panels report the derived complete fitted amplitude decays as a function of the relaxation delay.

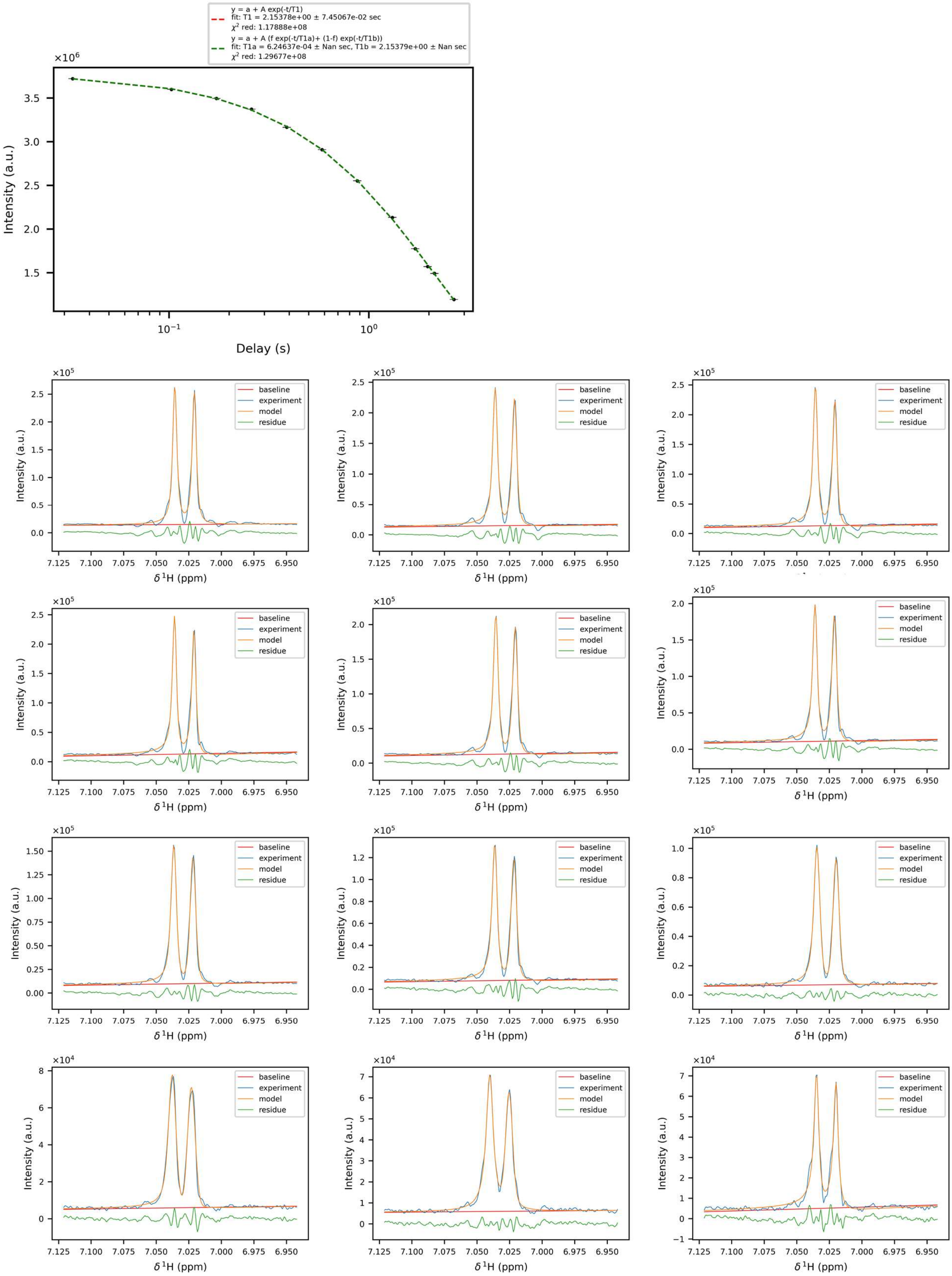
y = a + A exp(-t/T1)
fit: T1 = 2.15378e+00 ± 7.45067e-02 sec
χ² red: 1.17888e+08
y = a + A (f exp(-t/T1a)+ (1-f) exp(-t/T1b))
fit: T1a = 6.24637e-04 ± Nan sec, T1b = 2.15379e+00 ± Nan sec
χ² red: 1.29677e+08
Intensity (a.u.)
Delay (s)
baseline
experiment
model
residue
δ ¹H (ppm)

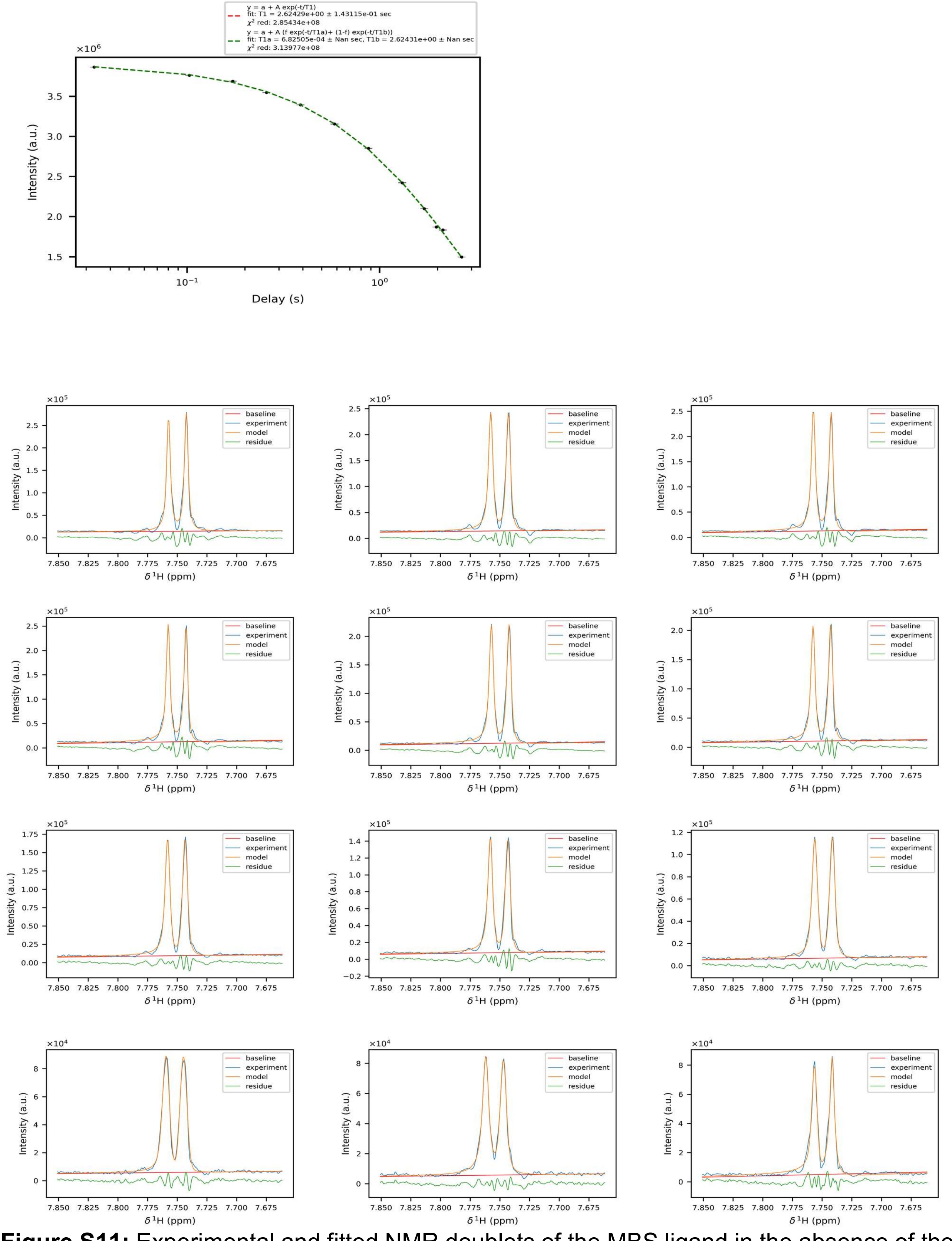

Figure S11: Experimental and fitted NMR doublets of the MBS ligand in the absence of the MMP-12 catalytic domain, acquired at 12 interleaved relaxation delays (0.1, 1.967, 1.311, 0.170, 0.873, 0.256, 0.581, 1.705, 2.131, 0.386, 2.664, 0.03 s, here displayed from shortest to longest) at 10000 mT/420 MHz. The first panels report the derived complete fitted amplitude decays as a function of the relaxation delay.

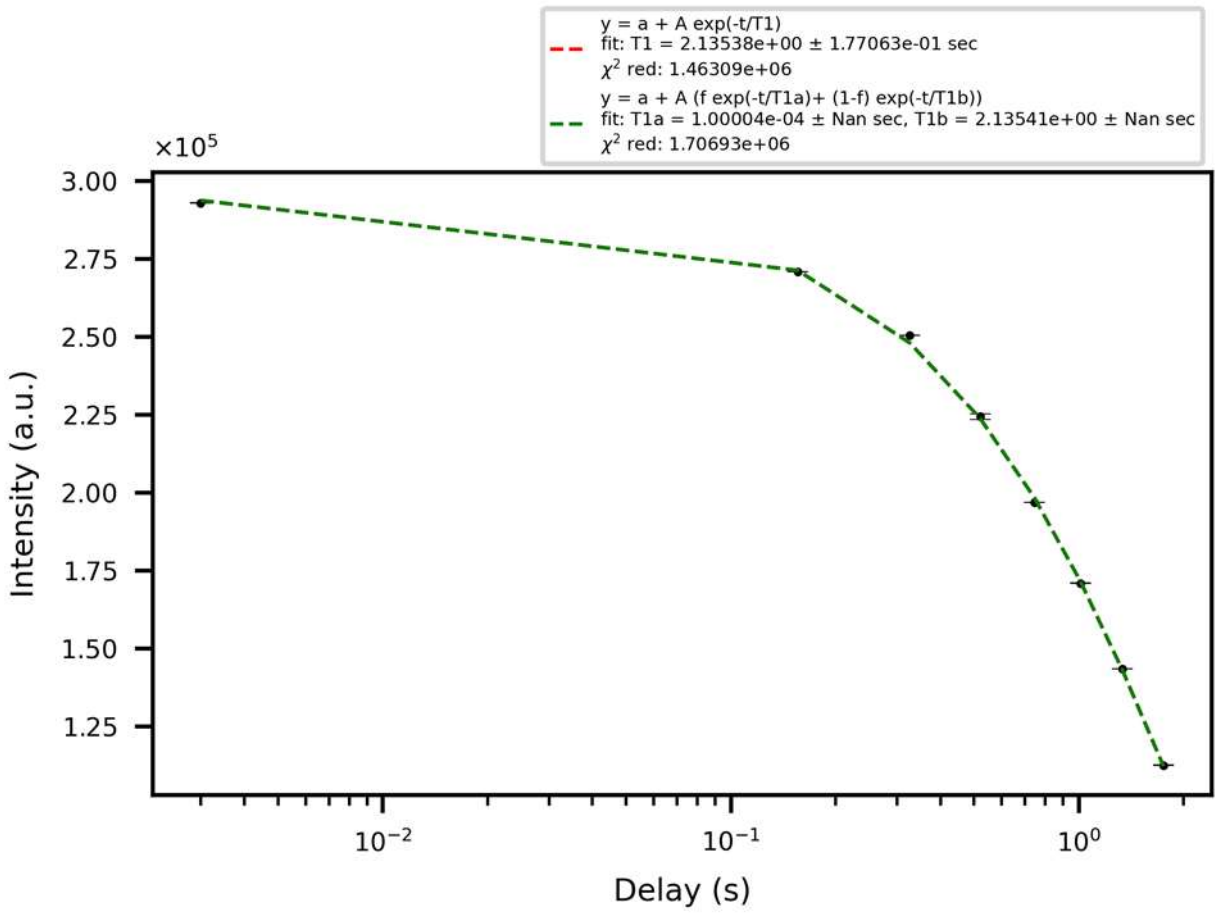
y = a + A exp(-t/T1)
fit: T1 = 2.13538e+00 ± 1.77063e-01 sec
χ² red: 1.46309e+06
y = a + A (f exp(-t/T1a)+ (1-f) exp(-t/T1b))
fit: T1a = 1.00004e-04 ± Nan sec, T1b = 2.13541e+00 ± Nan sec
χ² red: 1.70693e+06
Intensity (a.u.)
Delay (s)

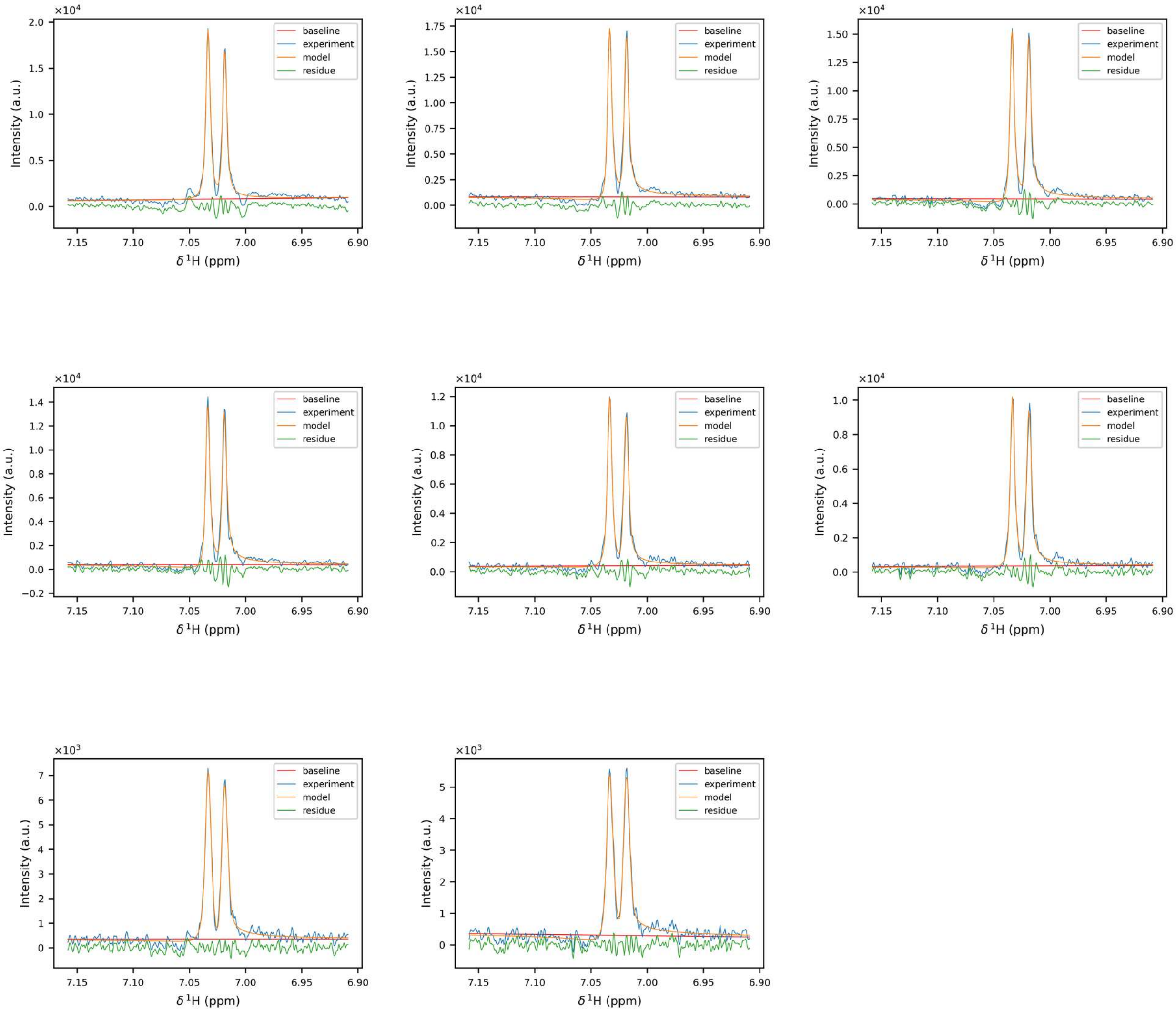
baseline
experiment
model
residue
Intensity (a.u.)
δ ¹H (ppm)

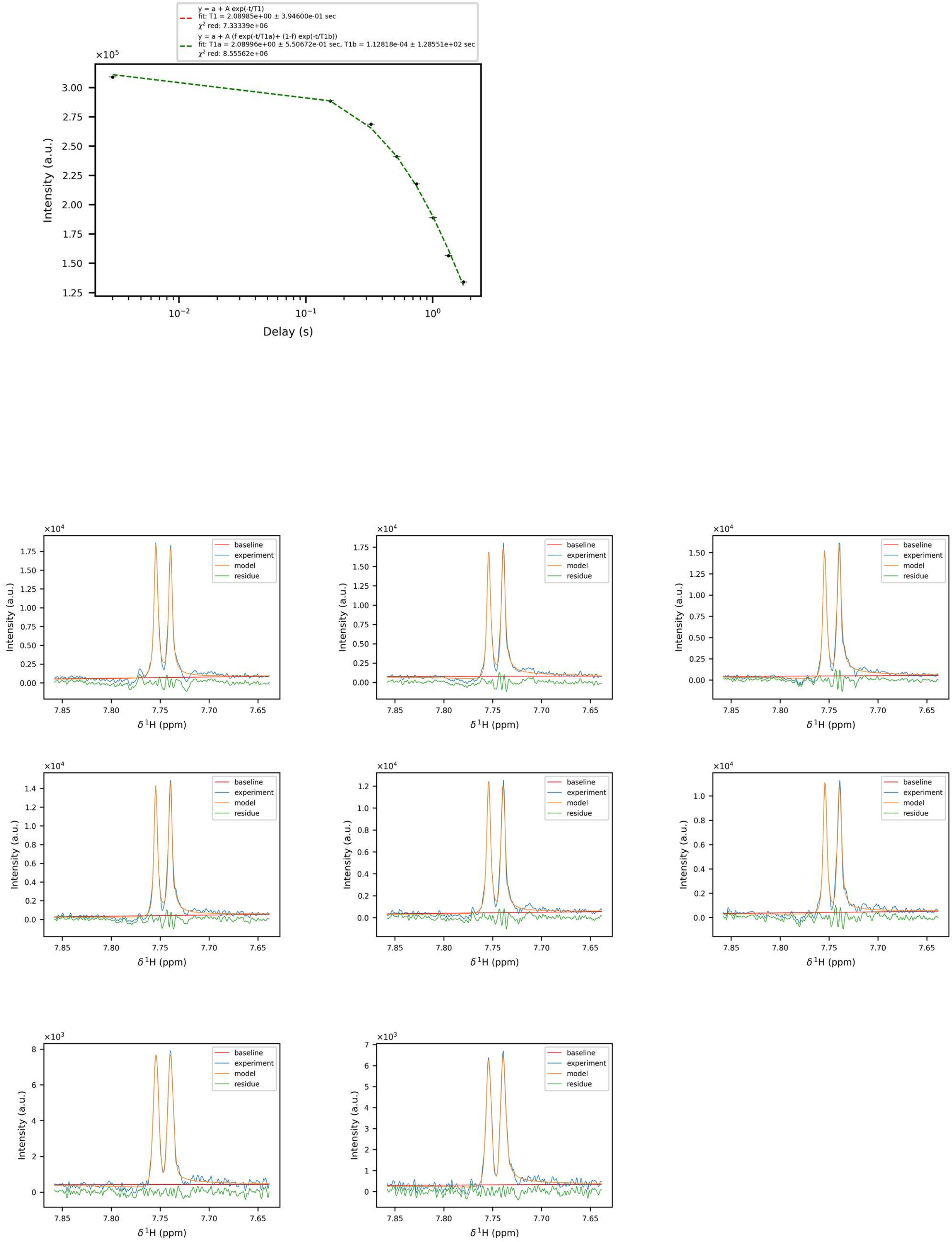

Figure S12: Experimental and fitted NMR doublets of the MBS ligand in the presence of the MMP-12 catalytic domain, acquired at 8 interleaved relaxation delays (0.000, 0.324, 0.153, 1.332, 0.518, 1.750, 0.742, 1.008 s, here displayed from shortest to longest) at 10000 mT/420 MHz. The first panels report the derived complete fitted amplitude decays as a function of the relaxation delay.

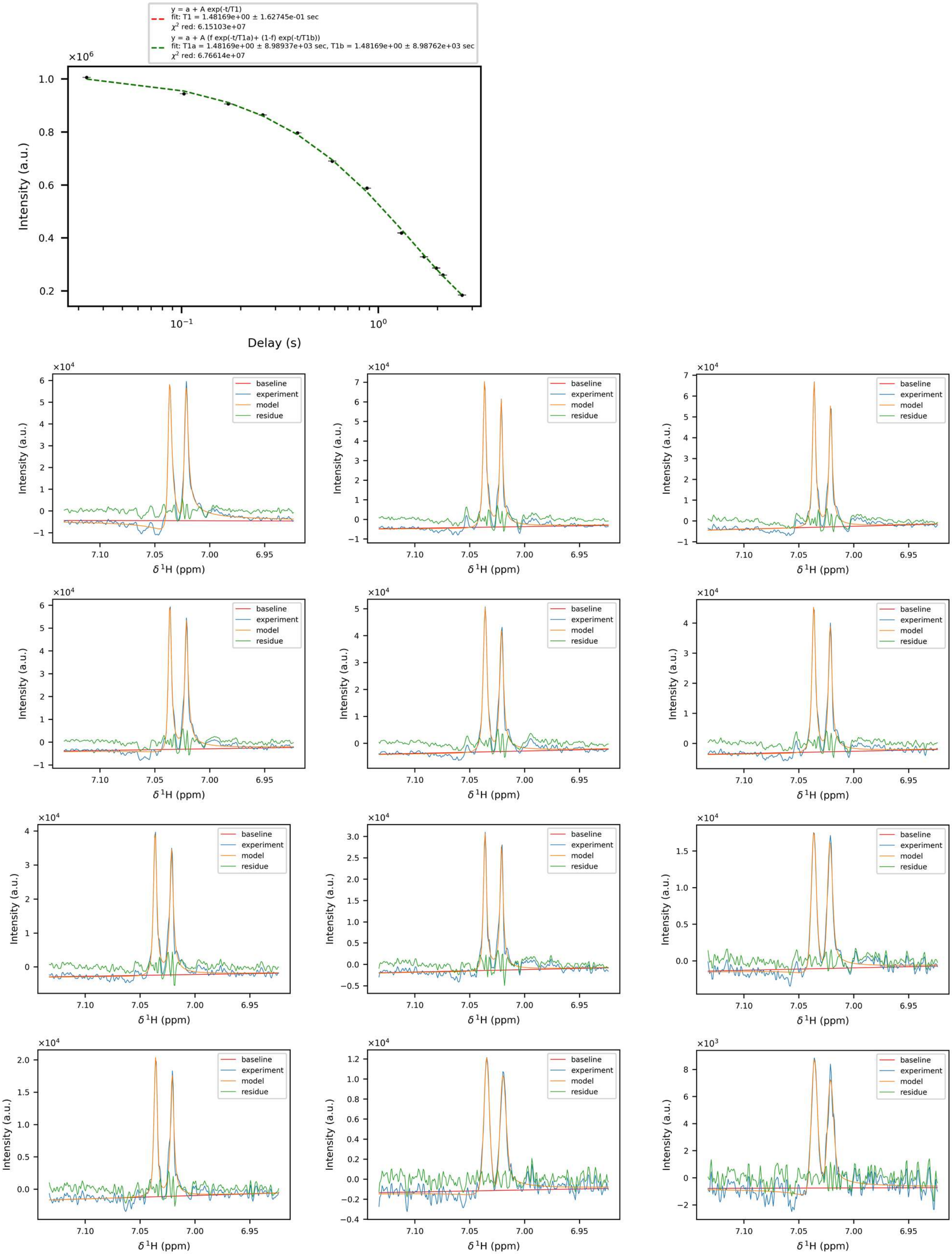
y = a + A exp(-t/T1)
fit: T1 = 1.48169e+00 ± 1.62745e-01 sec
χ2 red: 6.15103e+07
y = a + A (f exp(-t/T1a)+ (1-f) exp(-t/T1b))
fit: T1a = 1.48169e+00 ± 8.98937e+03 sec, T1b = 1.48169e+00 ± 8.98762e+03 sec
χ2 red: 6.76614e+07
Intensity (a.u.)
Delay (s)
baseline
experiment
model
residue
δ 1H (ppm)

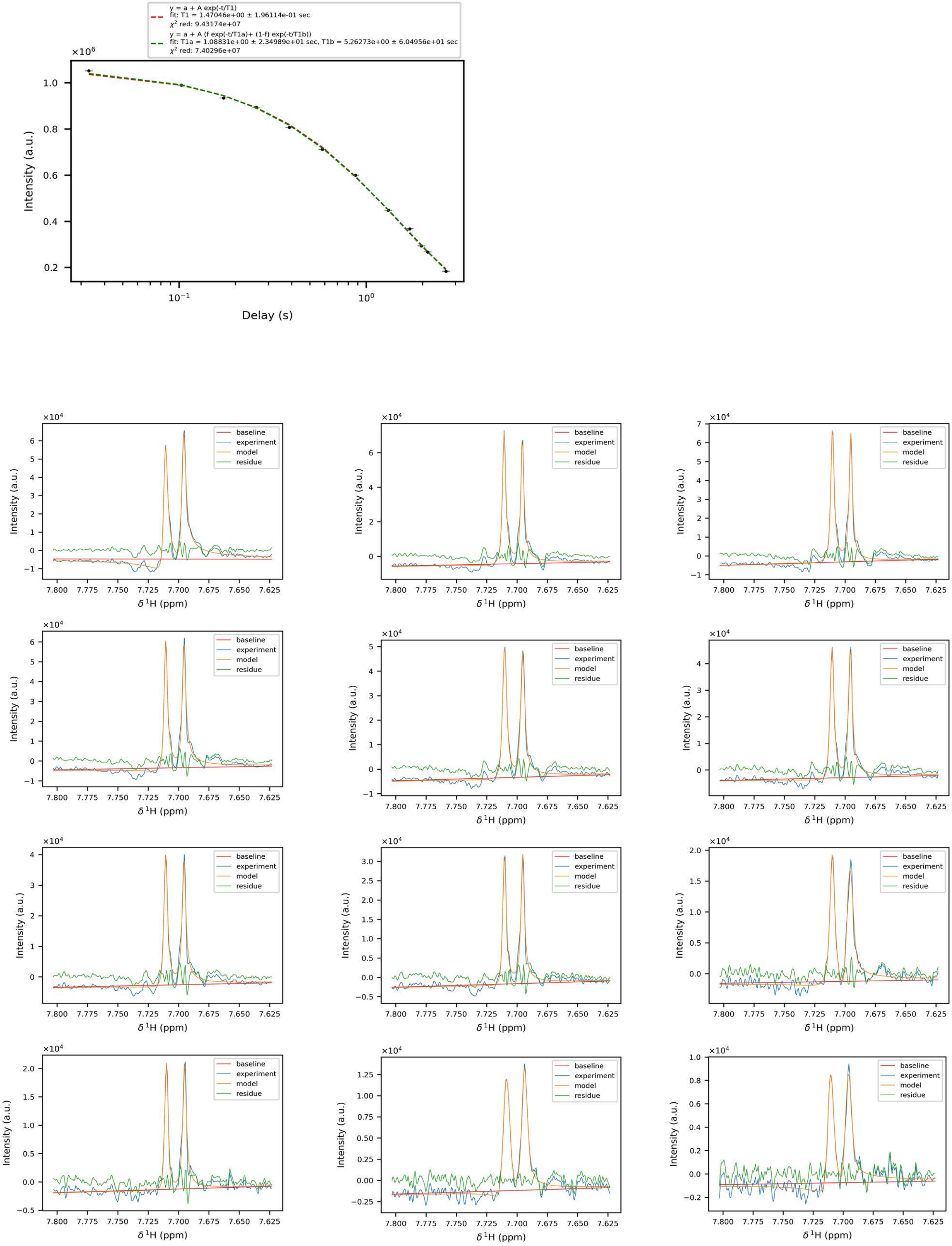

Figure S13: Experimental and fitted NMR doublets of the MCL ligand in the absence of the MMP-12 catalytic domain, acquired at 12 interleaved relaxation delays (0.1, 1.967, 1.311, 0.170, 0.873, 0.256, 0.581, 1.705, 2.131, 0.386, 2.664, 0.03 s, here displayed from shortest to longest) at 40 mT/1.7 MHz. The first panels report the derived complete fitted amplitude decays as a function of the relaxation delay.

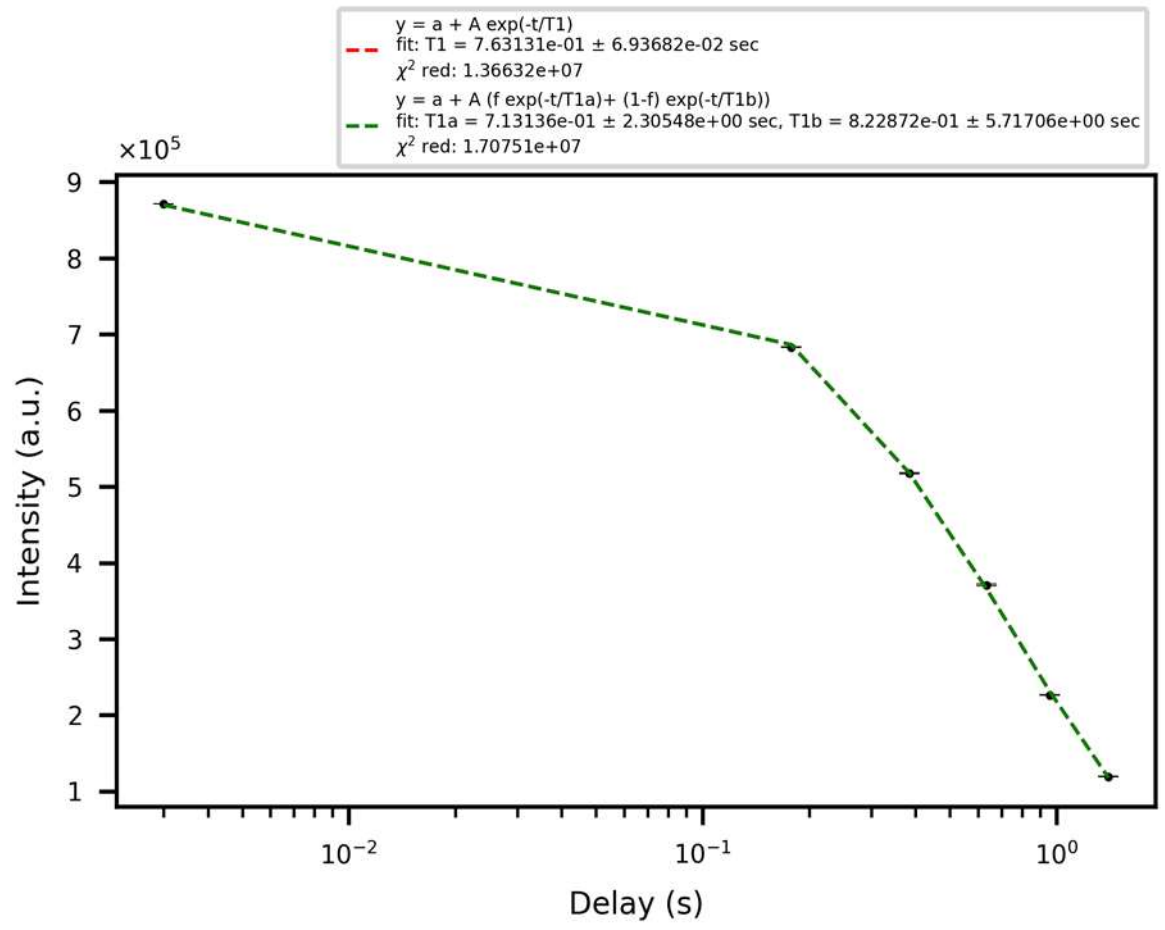
y = a + A exp(-t/T1)
fit: T1 = 7.63131e-01 ± 6.93682e-02 sec
χ² red: 1.36632e+07
y = a + A (f exp(-t/T1a)+ (1-f) exp(-t/T1b))
fit: T1a = 7.13136e-01 ± 2.30548e+00 sec, T1b = 8.22872e-01 ± 5.71706e+00 sec
χ² red: 1.70751e+07
Intensity (a.u.)
Delay (s)

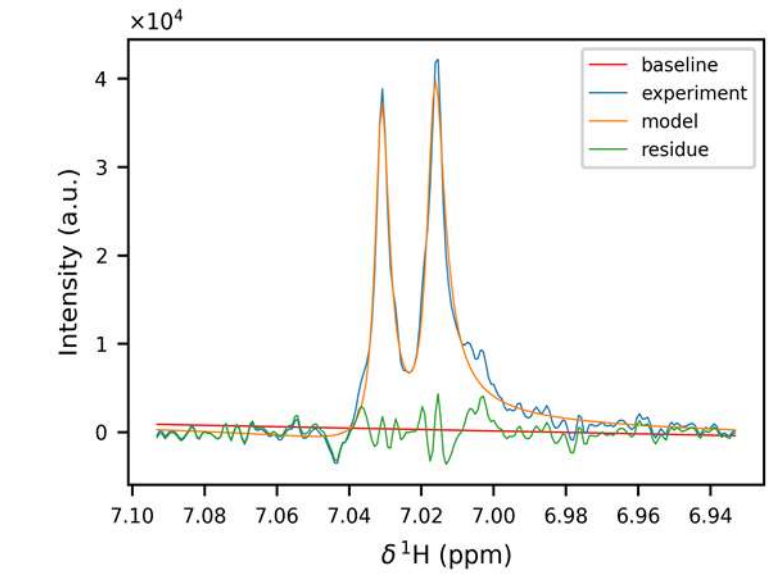
baseline
experiment
model
residue
Intensity (a.u.)
δ ¹H (ppm)

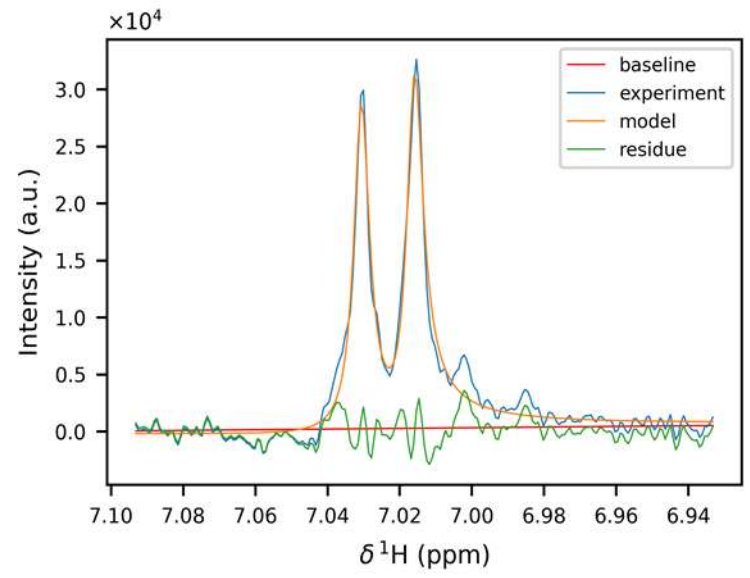
baseline
experiment
model
residue
Intensity (a.u.)
δ ¹H (ppm)

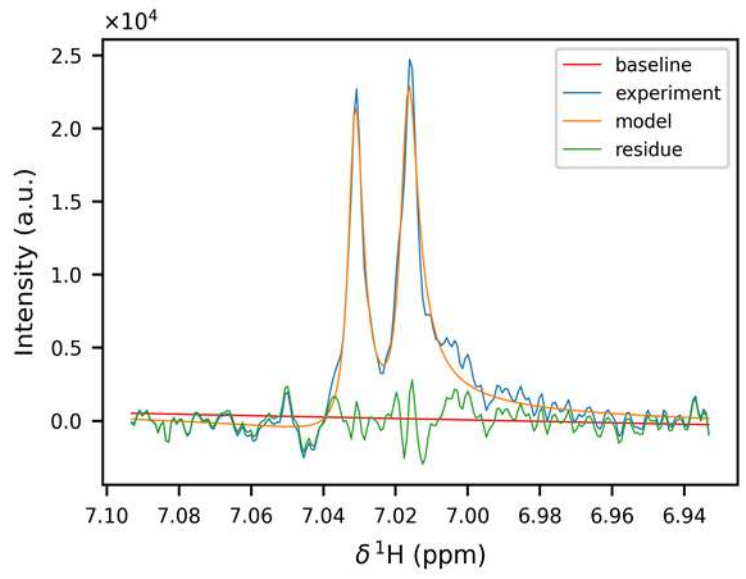
baseline
experiment
model
residue
Intensity (a.u.)
δ ¹H (ppm)

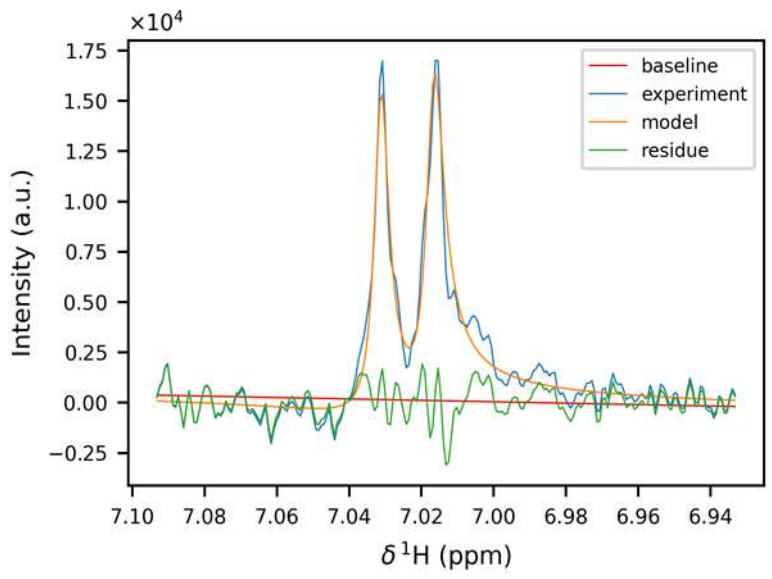
baseline
experiment
model
residue
Intensity (a.u.)
δ ¹H (ppm)

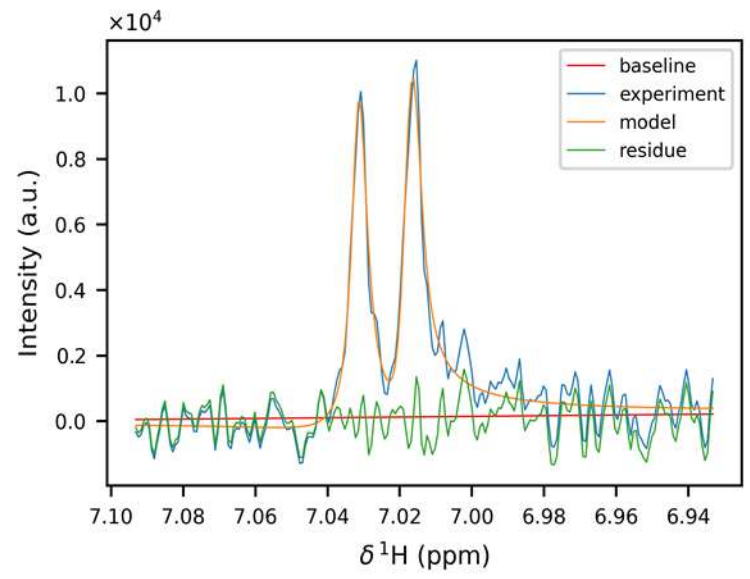
baseline
experiment
model
residue
Intensity (a.u.)
δ ¹H (ppm)

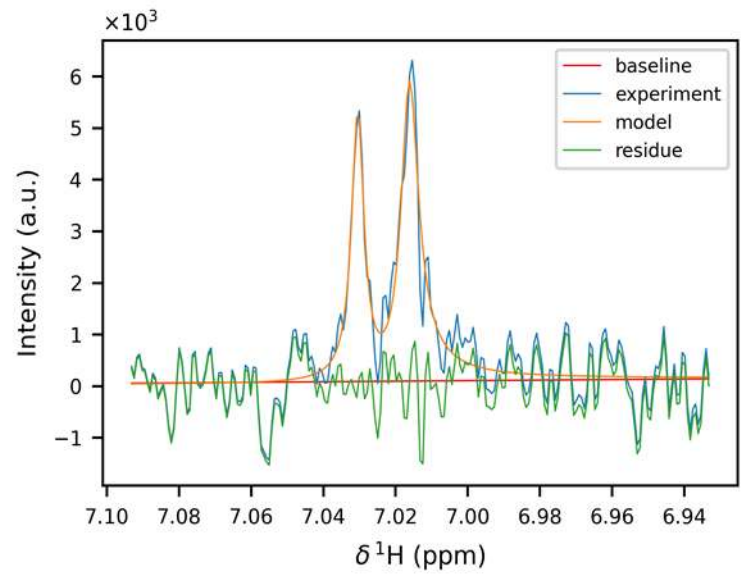
baseline
experiment
model
residue
Intensity (a.u.)
δ ¹H (ppm)

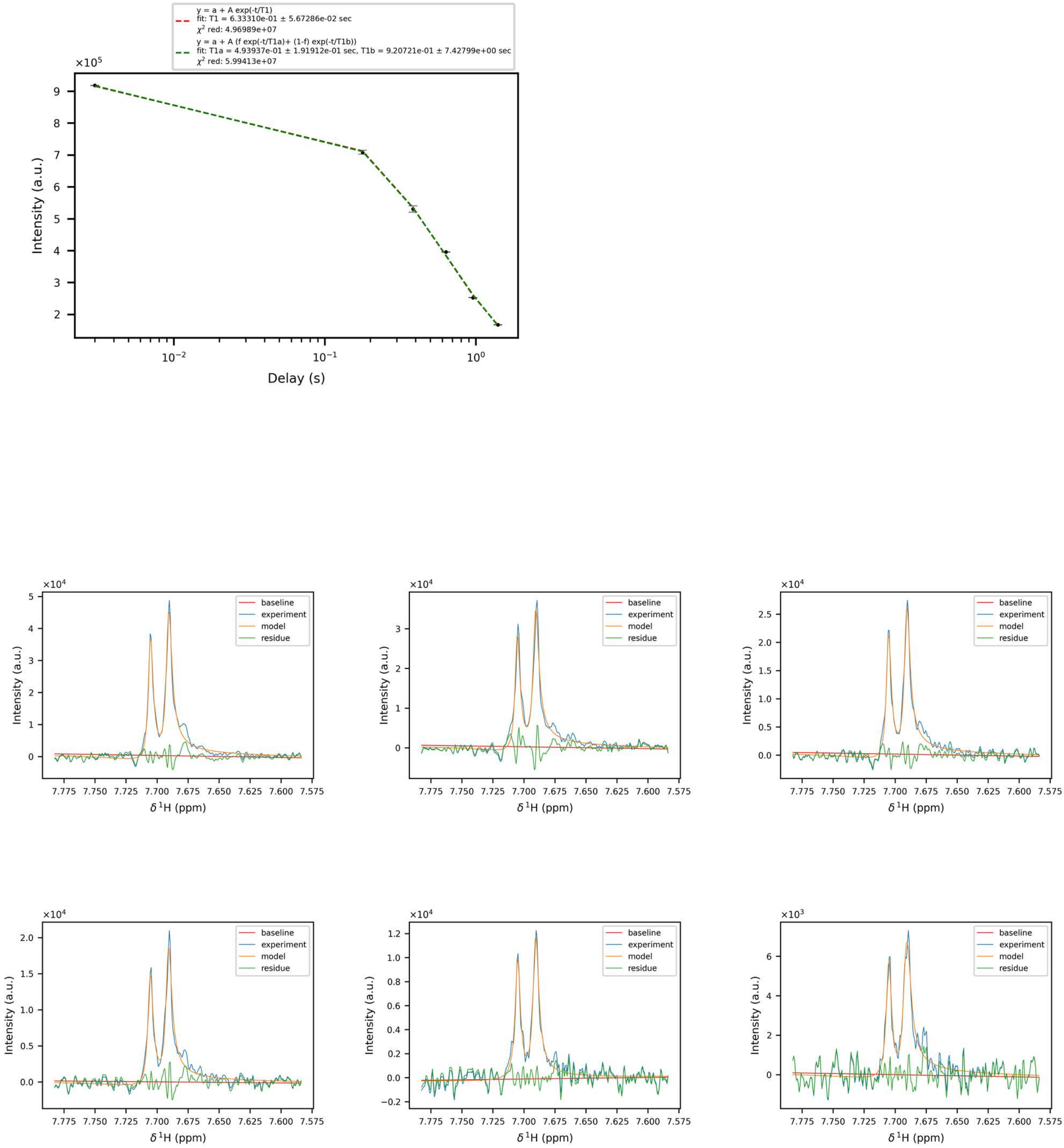

Figure S14: Experimental and fitted NMR doublets of the MCL ligand in the presence of the MMP-12 catalytic domain, acquired at 6 interleaved relaxation delays (0.955, 1.400, 0.175, 0.633, 0.000, 0.382 s, here displayed from shortest to longest) at 40 mT/1.7 MHz. The first panels report the derived complete fitted amplitude decays as a function of the relaxation delay.

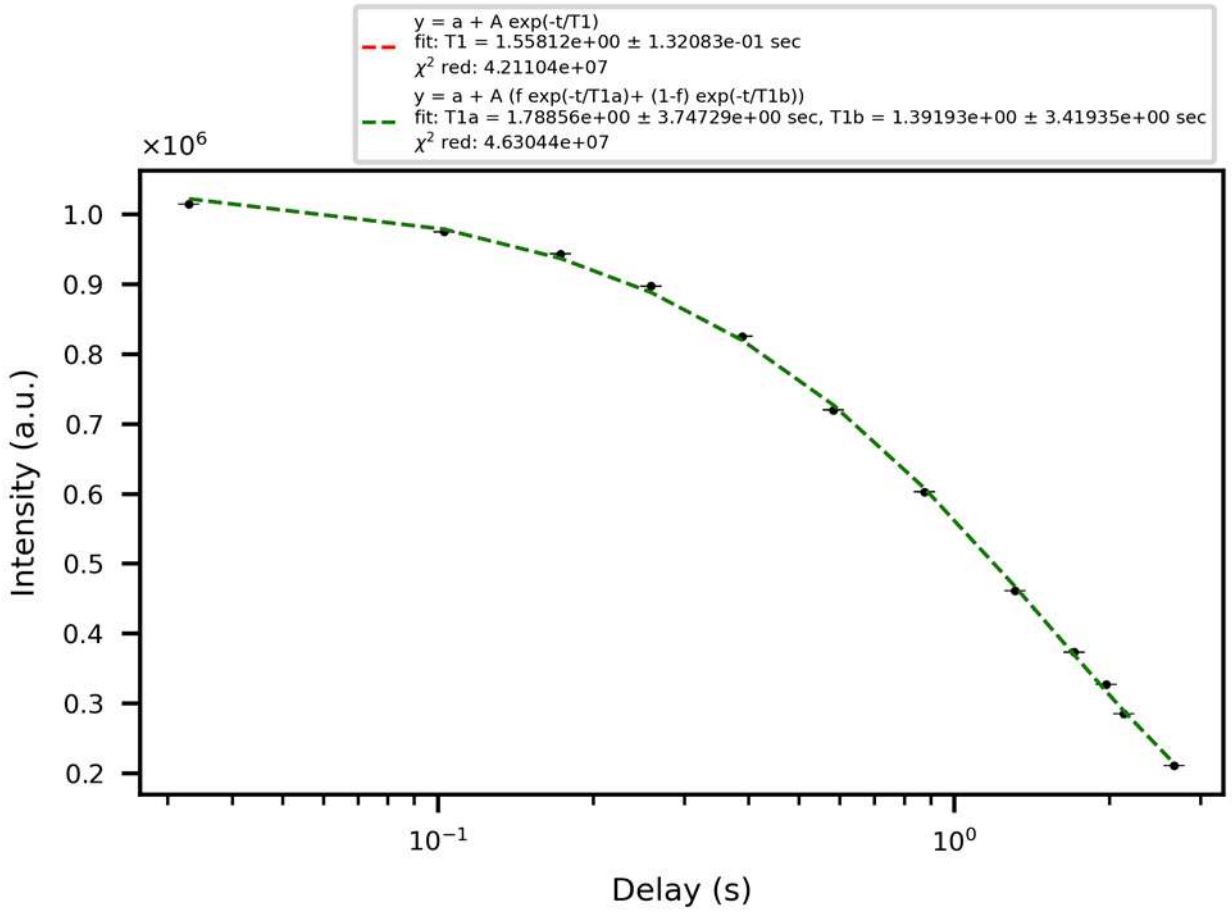
y = a + A exp(-t/T1)
fit: T1 = 1.55812e+00 ± 1.32083e-01 sec
χ² red: 4.21104e+07
y = a + A (f exp(-t/T1a)+ (1-f) exp(-t/T1b))
fit: T1a = 1.78856e+00 ± 3.74729e+00 sec, T1b = 1.39193e+00 ± 3.41935e+00 sec
χ² red: 4.63044e+07
×10⁶
Intensity (a.u.)
Delay (s)

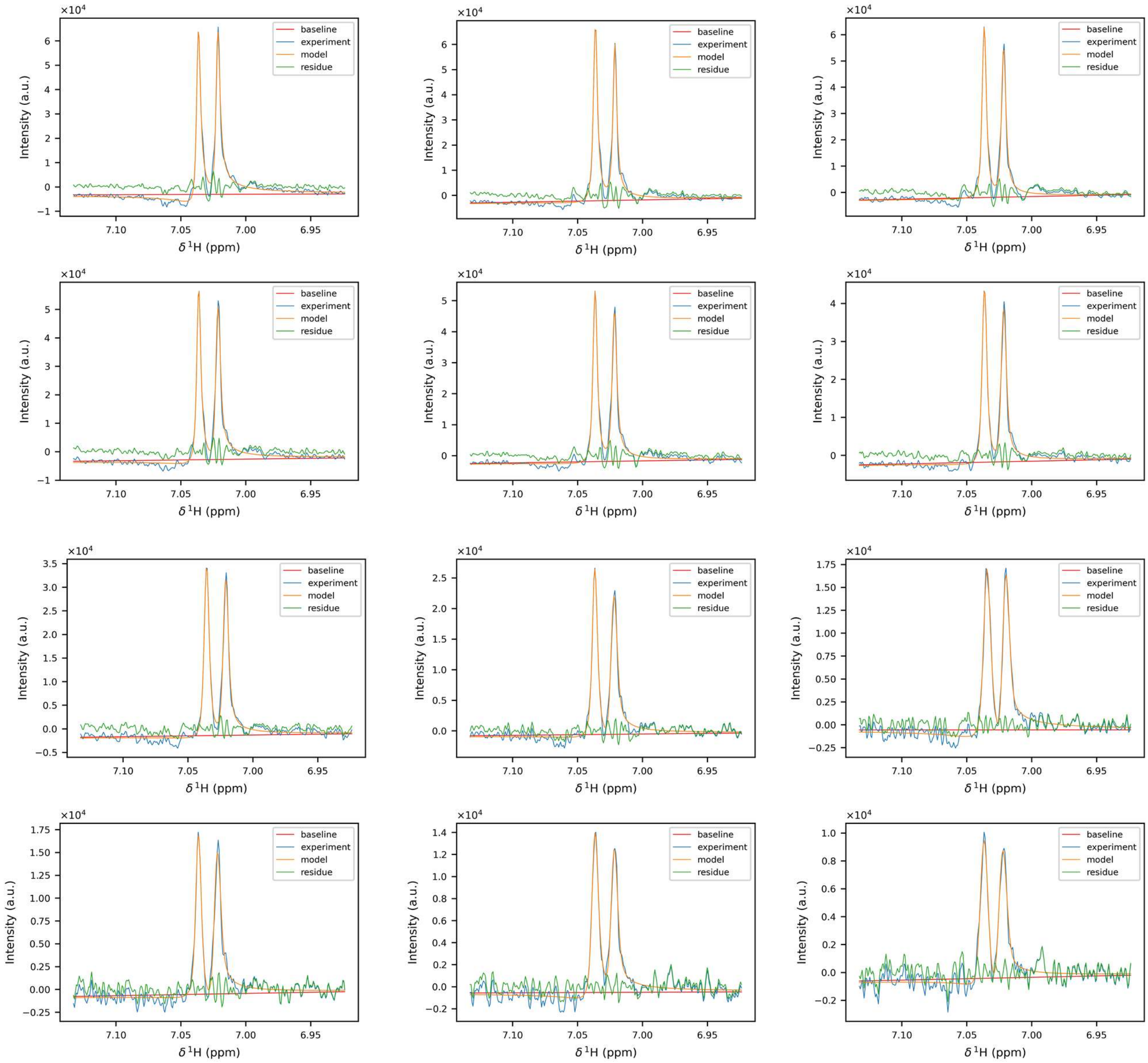
baseline
experiment
model
residue
Intensity (a.u.)
δ ¹H (ppm)

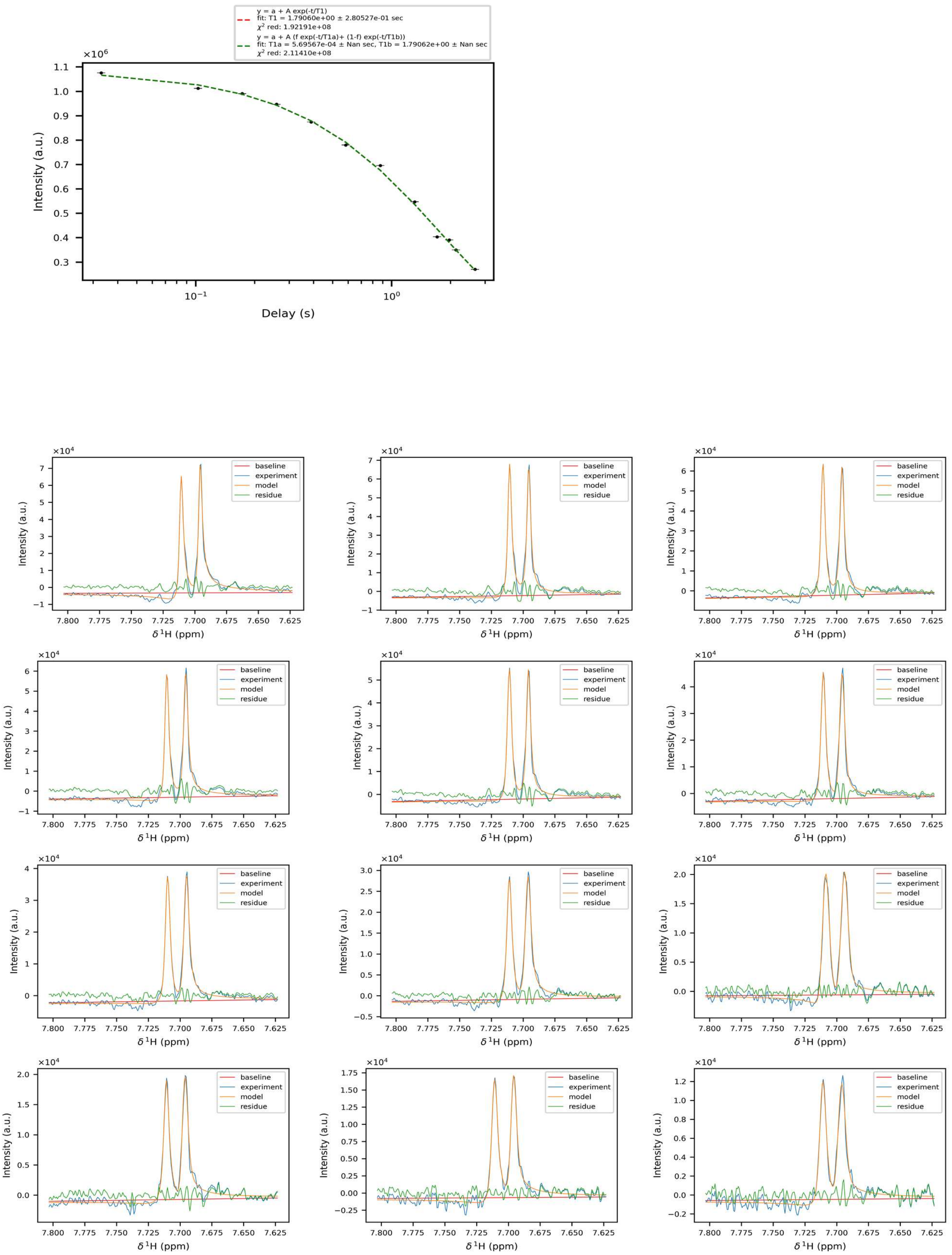

Figure S15: Experimental and fitted NMR doublets of the MCL ligand in the absence of the MMP-12 catalytic domain, acquired at 12 interleaved relaxation delays (0.1, 1.967, 1.311, 0.170, 0.873, 0.256, 0.581, 1.705, 2.131, 0.386, 2.664, 0.03 s, here displayed from shortest to longest) at 400 mT/17 MHz. The first panels report the derived complete fitted amplitude decays as a function of the relaxation delay.

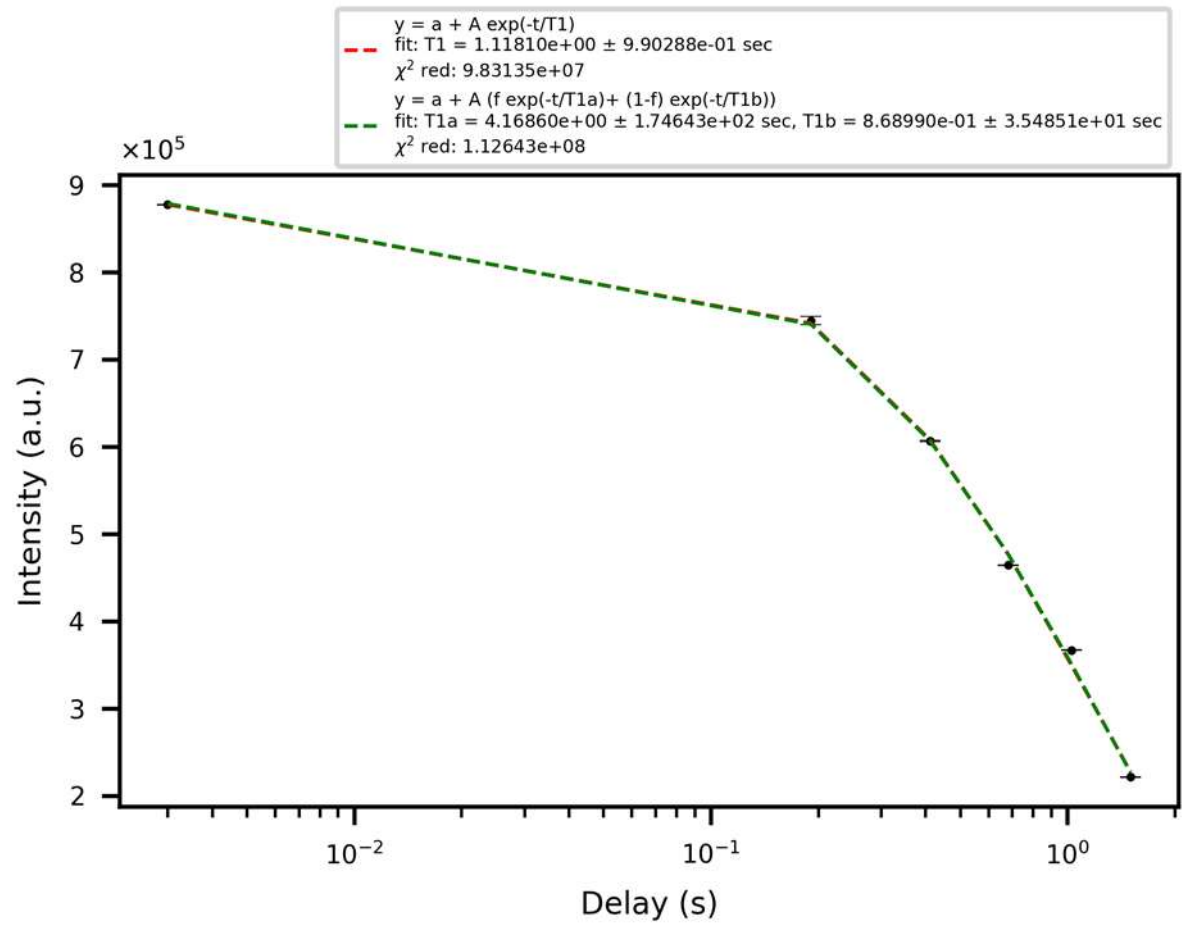
y = a + A exp(-t/T1)
fit: T1 = 1.11810e+00 ± 9.90288e-01 sec
χ² red: 9.83135e+07
y = a + A (f exp(-t/T1a)+ (1-f) exp(-t/T1b))
fit: T1a = 4.16860e+00 ± 1.74643e+02 sec, T1b = 8.68990e-01 ± 3.54851e+01 sec
χ² red: 1.12643e+08
×10⁵
Intensity (a.u.)
Delay (s)

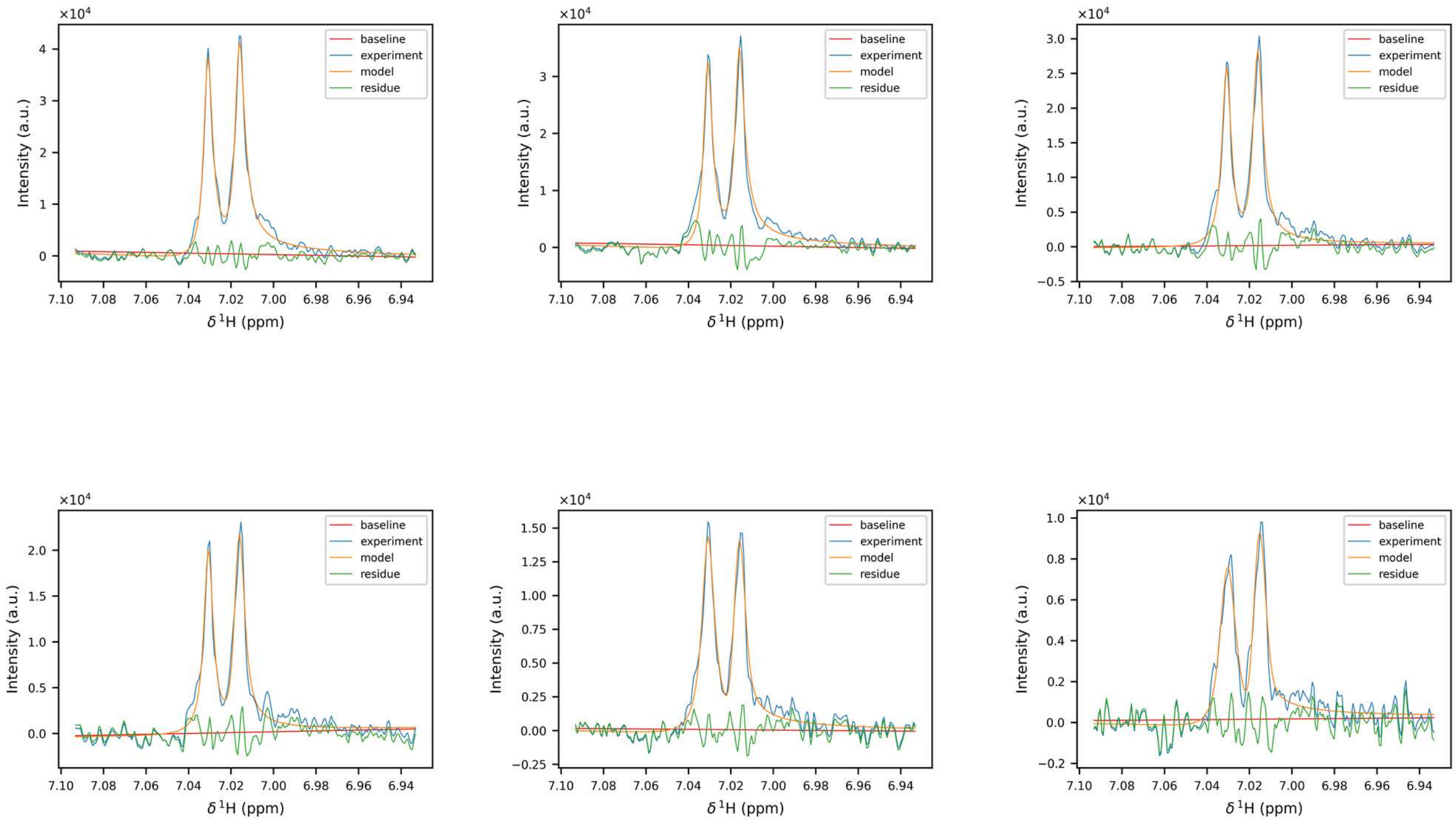
baseline
experiment
model
residue
Intensity (a.u.)
δ ¹H (ppm)

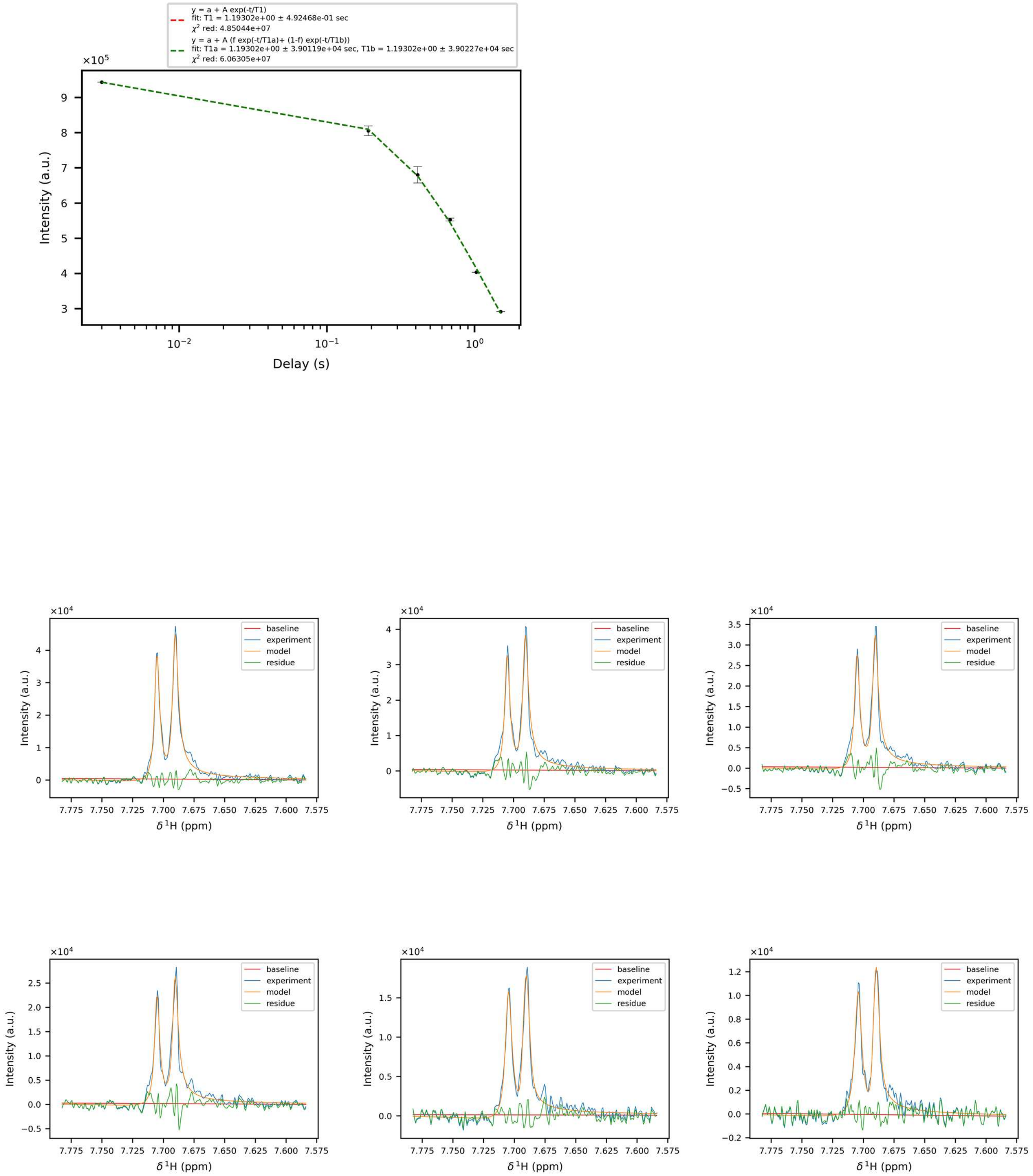

Figure S16: Experimental and fitted NMR doublets of the MCL ligand in the presence of the MMP-12 catalytic domain, acquired at 6 interleaved relaxation delays (1.500, 1.023, 0.000, 0.188, 0.409, 0.679 s, here displayed from shortest to longest) at 400 mT/17 MHz. The first panels report the derived complete fitted amplitude decays as a function of the relaxation delay.

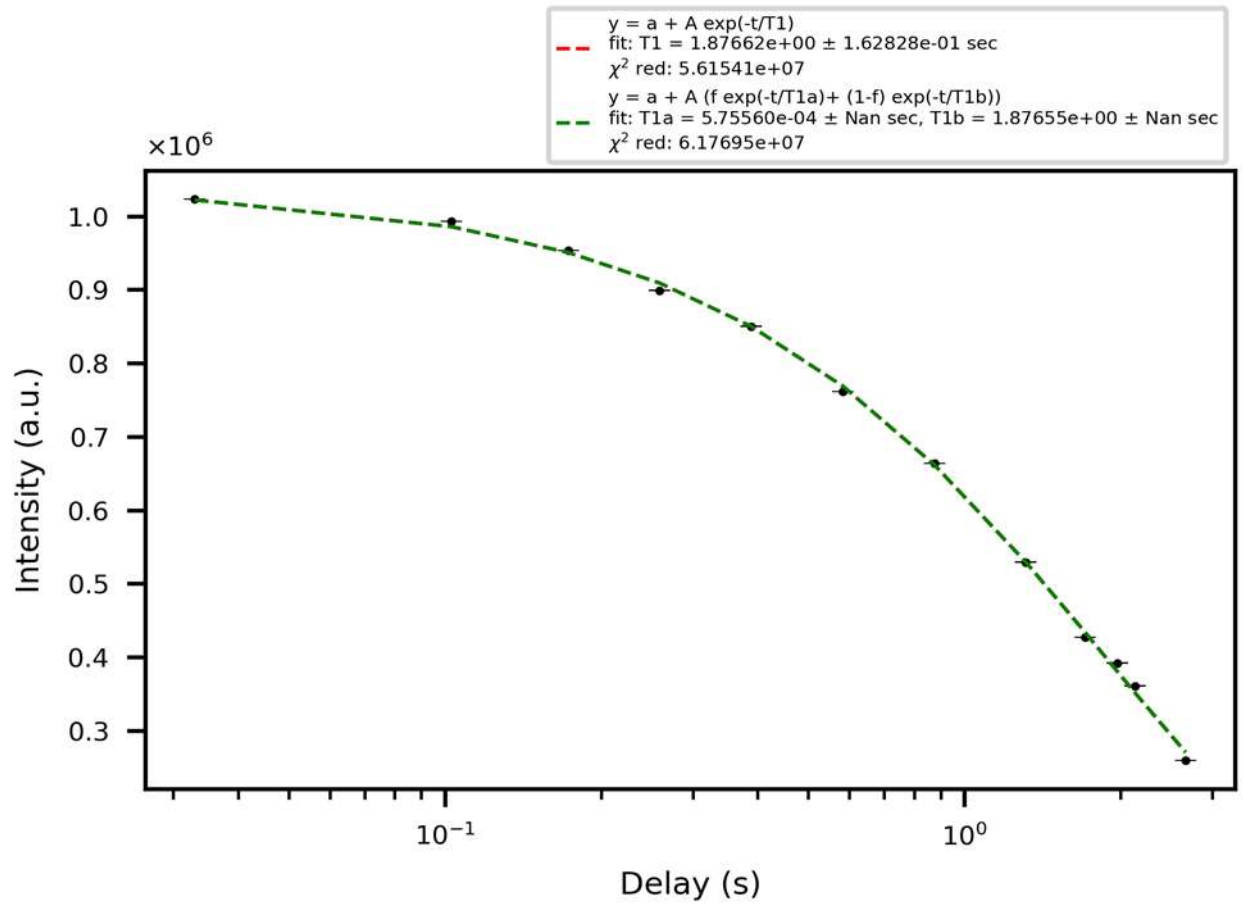
y = a + A exp(-t/T1)
fit: T1 = 1.87662e+00 ± 1.62828e-01 sec
χ² red: 5.61541e+07
y = a + A (f exp(-t/T1a)+ (1-f) exp(-t/T1b))
fit: T1a = 5.75560e-04 ± Nan sec, T1b = 1.87655e+00 ± Nan sec
χ² red: 6.17695e+07
×10⁶
Intensity (a.u.)
Delay (s)

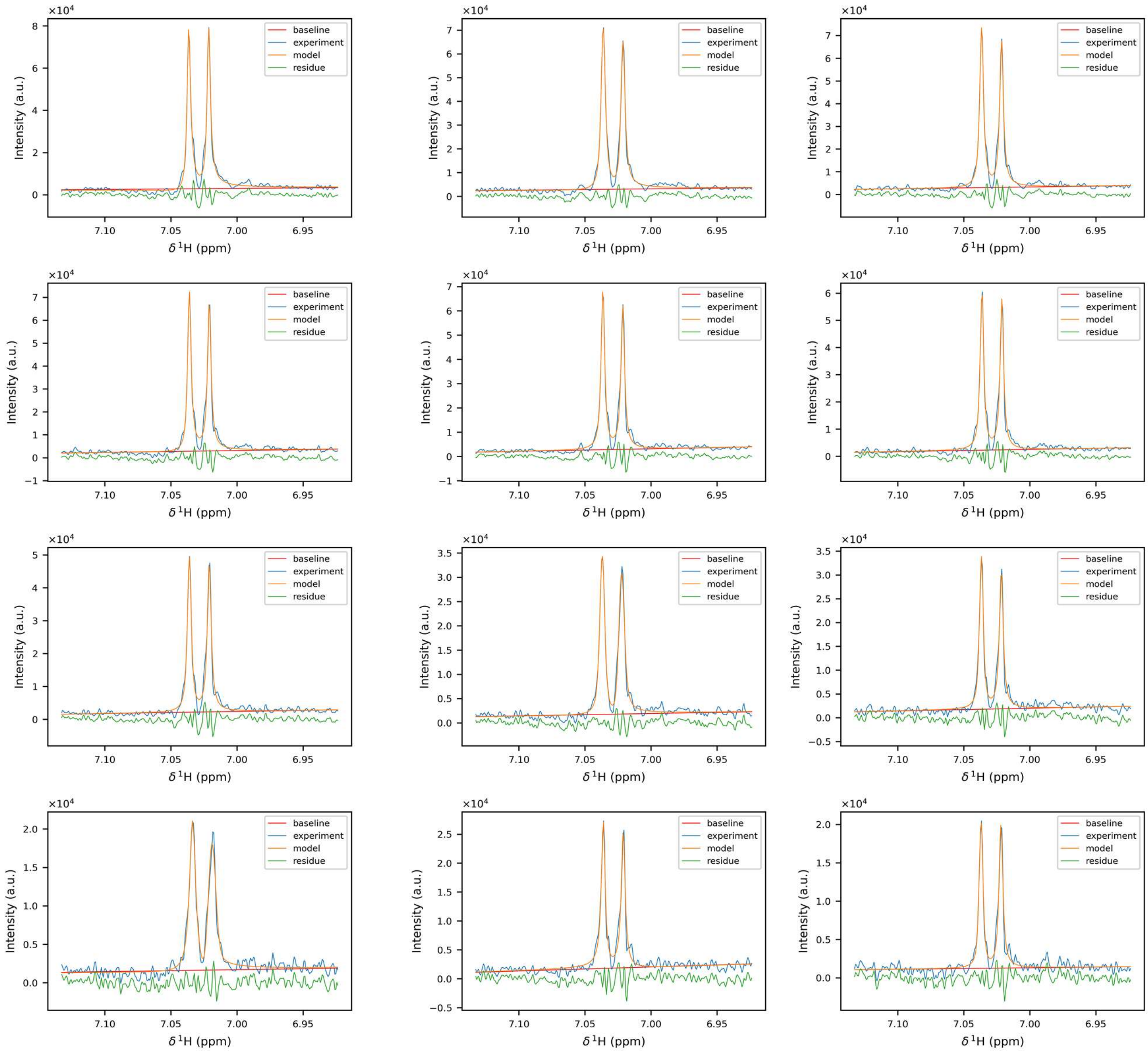
baseline
experiment
model
residue
×10⁴
Intensity (a.u.)
δ ¹H (ppm)

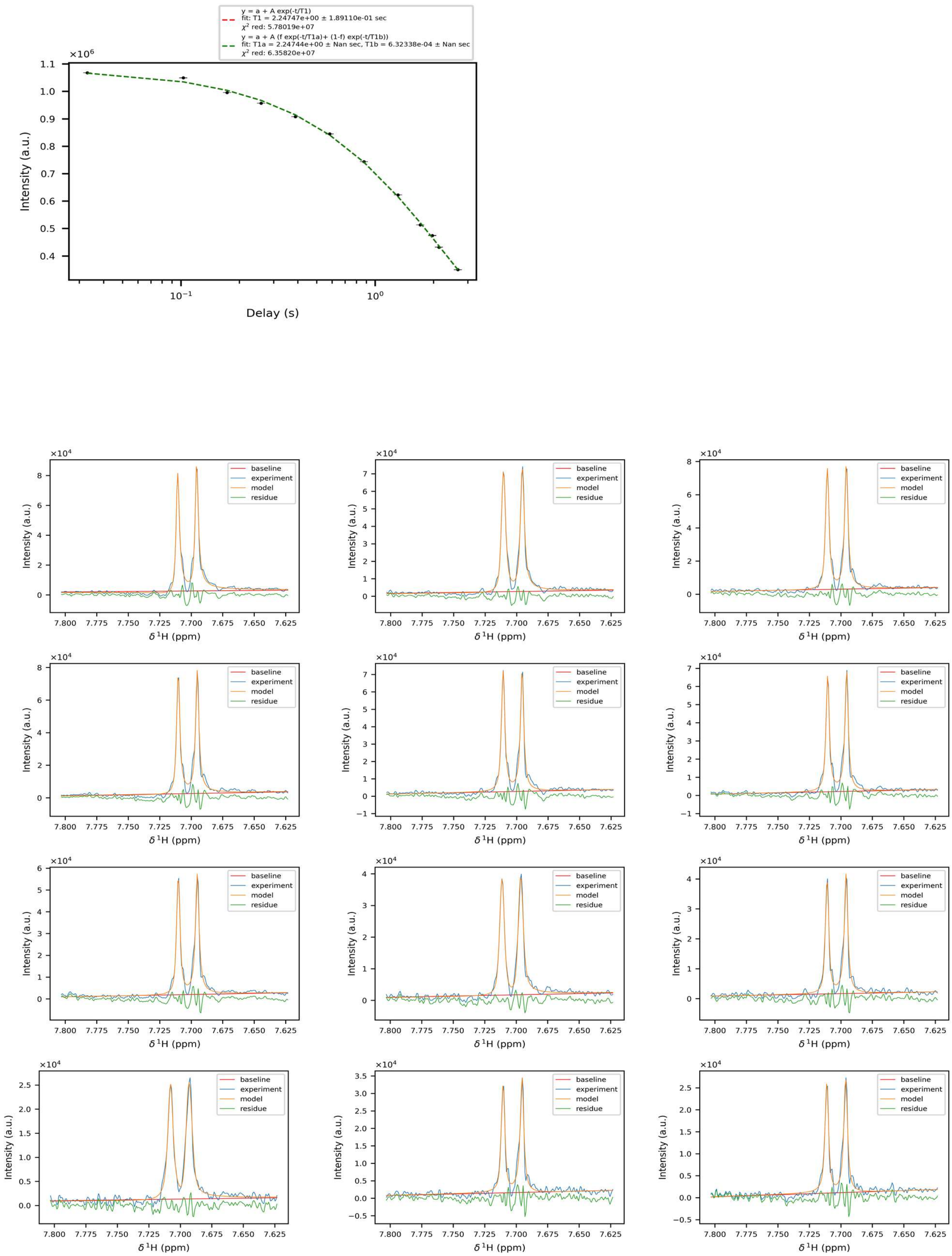

Figure S17: Experimental and fitted NMR doublets of the MCL ligand in the absence of the MMP-12 catalytic domain, acquired at 12 interleaved relaxation delays (0.1, 1.967, 1.311, 0.170, 0.873, 0.256, 0.581, 1.705, 2.131, 0.386, 2.664, 0.03 s, here displayed from shortest to longest) at 8000 mT/336 MHz. The first panels report the derived complete fitted amplitude decays as a function of the relaxation delay.

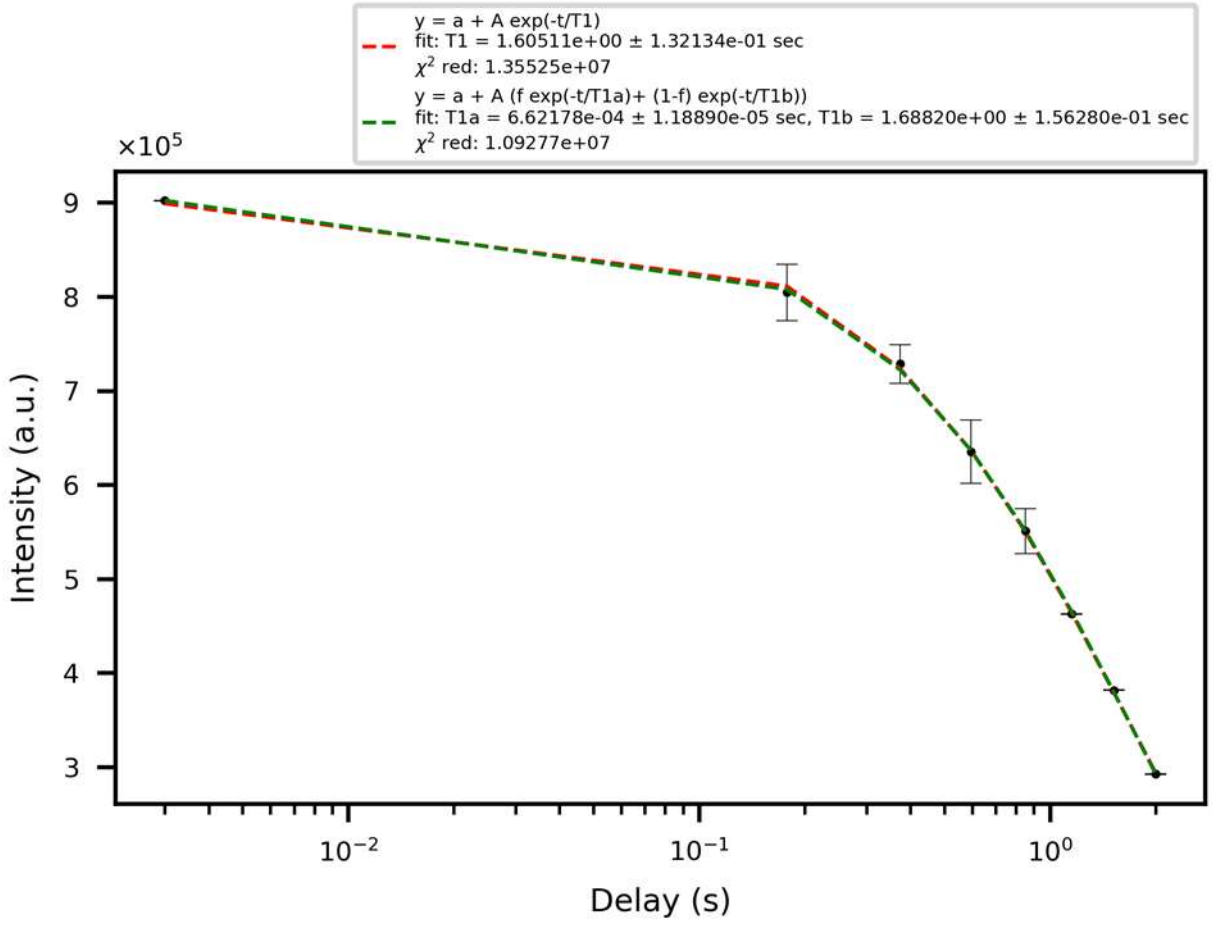
y = a + A exp(-t/T1)
fit: T1 = 1.60511e+00 ± 1.32134e-01 sec
χ² red: 1.35525e+07
y = a + A (f exp(-t/T1a)+ (1-f) exp(-t/T1b))
fit: T1a = 6.62178e-04 ± 1.18890e-05 sec, T1b = 1.68820e+00 ± 1.56280e-01 sec
χ² red: 1.09277e+07
Intensity (a.u.)
Delay (s)

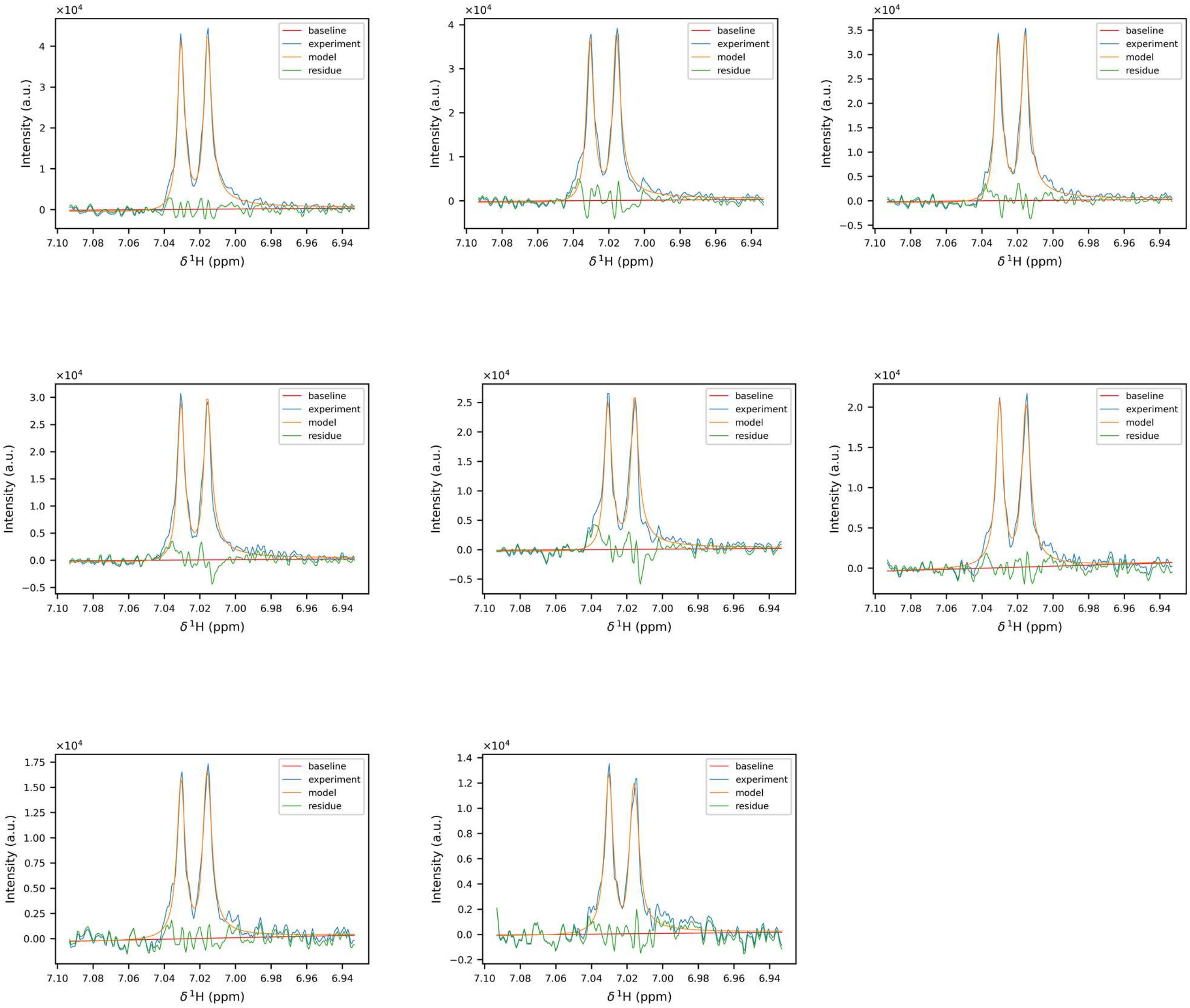
baseline
experiment
model
residue
Intensity (a.u.)
δ ¹H (ppm)

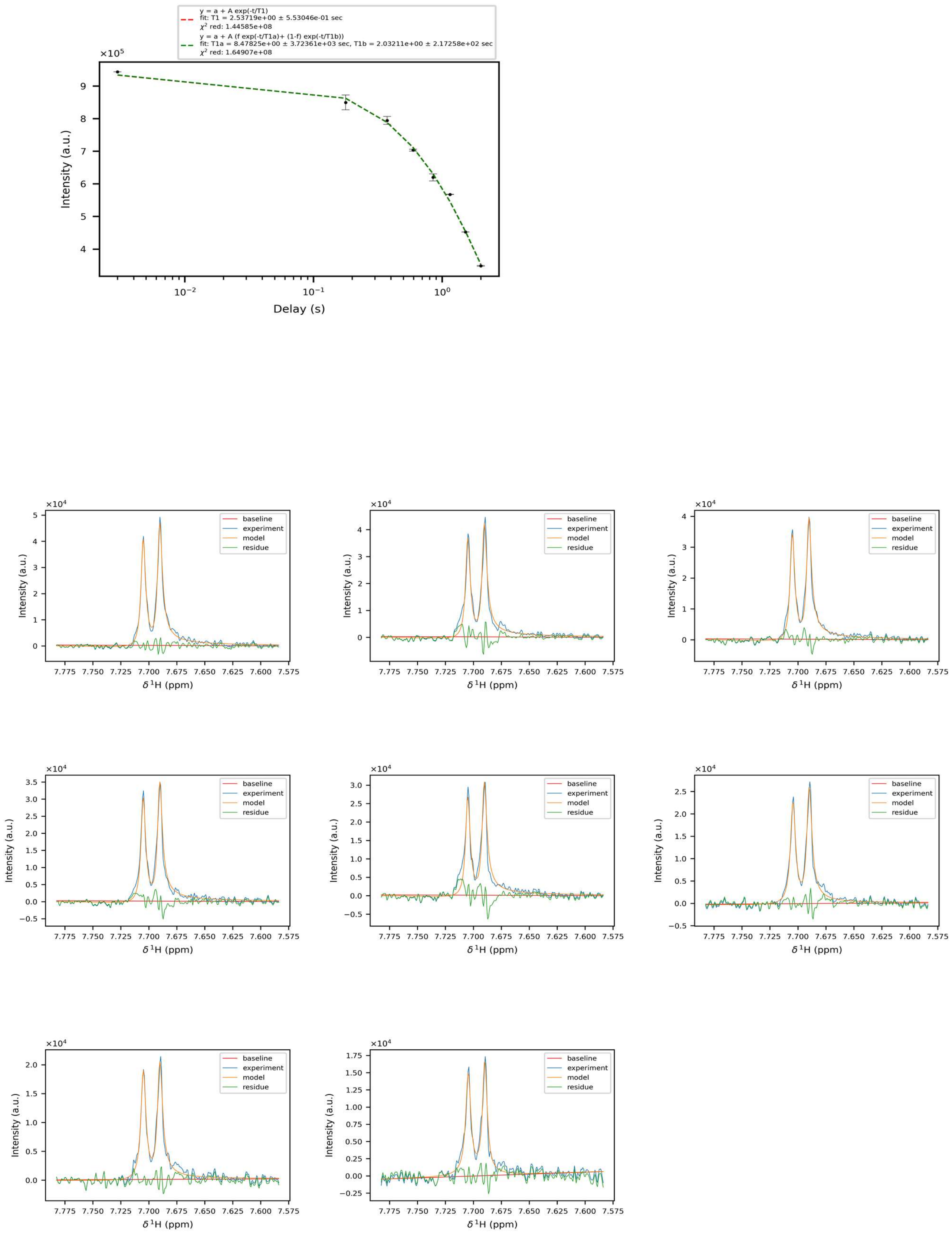

Figure S18: Experimental and fitted NMR doublets of the MCL ligand in the presence of the MMP-12 catalytic domain, acquired at 8 interleaved relaxation delays (0.175, 2.000, 0.849, 0.000, 1.151, 1.522, 0.371, 0.592 s, here displayed from shortest to longest) at 8000 mT/336 MHz. The first panels report the derived complete fitted amplitude decays as a function of the relaxation delay.
